# Propofol provides a significant survival advantage in sepsis-associated encephalopathy: A retrospective cohort study investigating one-year all-cause mortality

**DOI:** 10.1371/journal.pone.0340371

**Published:** 2026-02-05

**Authors:** Yuan Li, Han Huang, Bingxing Shuai

**Affiliations:** 1 Department of Anesthesiology and Key Laboratory of Birth Defects and Related Diseases of Women and Children, Ministry of Education, West China Second University Hospital of Sichuan University, Sichuan University, Chengdu, China; 2 Department of Anaesthesiology and Key Laboratory of Birth Defects and Related Diseases of Women and Children (Sichuan University), Ministry of Education, Children’s Medicine Key Laboratory of Sichuan Province, West China Second University Hospital, Sichuan University, Chengdu, China; 3 Department of Medical Affairs, West China Hospital, Sichuan University, Chengdu, China; University of Palermo, ITALY

## Abstract

**Introduction:**

Considering the high incidence, mortality, and long-term effects of sepsis-associated encephalopathy (SAE), along with the availability of sedation therapy data and the significance of distress management, this study investigated the relationship between sedation therapy and one-year all-cause mortality in patients with SAE.

**Methods:**

This retrospective cohort study utilized the Medical Information Market for Intensive Care (MIMIC-IV) database. We gathered demographic data, vital signs, laboratory test results, microbial findings, comorbidities, scoring systems, treatments administered within the first 24 hours of patient admission to the intensive care unit (ICU), and follow-up data from 24 hours after ICU admission to one year. Cox regression models were employed to evaluate the relationship between sedation therapy and one-year all-cause mortality among patients with SAE. Propensity score matching (PSM) and subgroup analyses were used to assess the robustness of the findings.

**Results:**

Four thousand six hundred eighteen patients with SAE were enrolled, including 3,343 in the sedative group and 1,275 in the non-sedative group; additionally, 511 pairs were matched. A protective correlation was observed between propofol monotherapy and one-year all-cause mortality in patients with SAE, with hazard ratios (HRs) of 0.51 (95% confidence interval (CI), 0.40–0.65), *P* < 0.001. Furthermore, the interaction between propofol monotherapy and ventilation support significantly increased one-year all-cause mortality, yielding an HR of 0.70 (95% CI, 0.49–1.00) with *P* for interaction = 0.041. The results of PSM remained robust.

**Conclusion:**

Our study indicated that compared to patients with SAE who did not receive sedation, administering propofol alone significantly reduced one-year all-cause mortality among patients with SAE. For patients undergoing ventilation support on the first day of ICU admission, the interaction between sedation therapy and ventilation support significantly influenced the one-year all-cause mortality of patients with SAE.

## 1 Introduction

Sepsis-associated encephalopathy (SAE)—an acutely progressive brain dysfunction that occurs alongside systemic inflammatory response syndrome (SIRS) or sepsis-induced systemic inflammation—is characterized by the absence of direct central nervous system (CNS) infection [1,2]. The reported incidence of SAE ranges from 53% to 70% in patients admitted to the intensive care unit (ICU) [3,4]. Furthermore, the mortality rate increases with SAE severity, reaching nearly 50% to 70% in patients with severe SAE [2,4]. Long-term follow-ups indicate that approximately 45% of sepsis survivors experience cognitive dysfunction, such as inattention and memory loss, one year after discharge. Moreover, up to 51% of patients with sepsis are unable to resume full-time employment within one year of their sepsis event [5,6].

The distress caused by pain, fear, anxiety, dyspnea, or delirium is common among critically ill patients, particularly those who are intubated or have difficulty communicating with their caregivers [7]. This distress may clinically manifest as agitation, often linked to ventilator asynchrony and increased sympathetic tone, potentially leading to adverse clinical effects [8]. Pharmacologic agents should be administered for patients whose treatment for the underlying critical illness and nonpharmacologic strategies have failed to sufficiently minimize distress (e.g., if the patient remains calm and cooperative). This approach helps reduce the risk of acute and chronic physical and emotional harm when distress is appropriately addressed. For patients with agitation due to anxiety requiring a continuous intravenous sedative infusion, previous studies recommended propofol or dexmedetomidine over benzodiazepines [9]. A 2018 network meta-analysis of 31 randomized trials involving 4491 patients indicated that the mortality rate was not linked to either propofol or benzodiazepine administration compared to dexmedetomidine upon discharge from the ICU and hospital [10]. However, no large-scale cohort studies have systematically examined whether sedation therapy independently correlates with one-year all-cause mortality following ICU admission, creating a gap that hinders personalized therapeutic strategies.

To address this challenge, we conducted a retrospective study examining the relationship between sedation therapy and one-year all-cause mortality. Our goal was identifying the optimal sedation combinations to help critical care clinicians manage patients with SAE more effectively.

## 2 Methods

### 2.1 Setting

This retrospective cohort study used data from the Medical Information Mart for Intensive Care (MIMIC)-IV v3.1 database [11], which includes a substantial number of deidentified records of patients admitted to the emergency department or ICU at Beth Israel Deaconess Medical Center in Boston, MA. From 2008 to 2022, MIMIC-IV v3.1 contained data from 94,458 ICU patients and 364,627 emergency department patients. The Institutional Review Board reviewed the collection of patient information and the creation of research resources at the Beth Israel Deaconess Medical Center, which granted a waiver of informed consent and approved the data-sharing initiative. One of the authors, Yuan Li, completed the required Collaborative Institutional Training Initiative (CITI PROGRAM) training (certification number: 58966597) and signed the Data Use Agreement to gain access to the database. This study followed the Strengthening the Reporting of Observational Studies in Epidemiology statement [12].

### 2.2 Participants

Patients with sepsis were defined according to the third edition of the sepsis diagnostic criteria (Sepsis 3.0), which incorporates the sequential organ failure assessment score (SOFA≥2) and suspected or confirmed infection [13]. The inclusion criteria were as follows [14]: 1. patients met the diagnostic criteria of Sepsis 3.0; 2. data were from the first ICU stay; 3. ICU stay was ≥ 24 h; 4. age ranged from 18–89 years. The exclusion criteria were as follows [4,15–17]: 1. brain injuries, such as traumatic brain injury, meningitis, encephalitis, intracerebral hemorrhage, cerebral embolism, ischemic stroke, epilepsy, or other cerebrovascular diseases (S1–S5 Tables); 2. mental disorders and neurological diseases (S6 Table); 3. chronic alcohol or drug abuse (S7 Table), minimizing the potential influence of withdrawal-related sedative use; 4. metabolic encephalopathy, hepatic encephalopathy, hypertensive encephalopathy, hypoglycemic coma, and other liver or kidney diseases affecting consciousness (S8 Table). Patients with chronic hepatic or renal disorders known to cause altered consciousness were excluded; 5. severe electrolyte imbalances or glycemic disturbances, including hyponatremia (<120 mmol/L), hypernatremia (>150 mmol/L), hyperglycemia (>180 mg/dL), hypoglycemia (<54 mg/dL); 6. a partial pressure of carbon dioxide (PaCO_2_) ≥80 mmHg; 7. missing GCS values. To minimize sample loss and reduce potential selection bias, SAE was diagnosed according to three previously published criteria [18–20]: (1) a Glasgow Coma Scale (GCS) score < 15 on the first day of ICU admission, (2) a diagnosis of delirium based on ICD-9 codes (2930, 2931) or ICD-10 code (F05) in patients with sepsis, or (3) treatment with haloperidol during hospitalization. Meeting any one of these criteria was considered sufficient for the diagnosis of SAE.

### 2.3 Variables

We extracted variables from the database across several domains: 1. demographic variables, including age, sex, race and recent surgery within 24 hours before ICU admission, which was identified by ICD-9/10 procedure codes; 2. vital signs, such as heart rate, systolic blood pressure, diastolic blood pressure, mean arterial pressure, respiratory rate, body temperature, and peripheral capillary oxygen saturation (SpO2); 3. laboratory test parameters, including blood urea nitrogen (BUN), creatinine, glucose, partial pressure of oxygen (PaO2), partial pressure of arterial carbon dioxide (PaCO2), lactate, sodium, potassium, hemoglobin levels, white blood cell count (WBC), platelet count, international normalized ratio (INR), prothrombin time (PT), and partial thrombin time (PTT); 4. site of infection, pathogenic microorganisms, and comorbidities; 5. disease severity scores, such as the Charlson Comorbidity Index (CCI), SOFA score, and simplified acute physiology score II (SAPS II); and 6. treatments administered within the first 24 hours of ICU admission, including ventilation, vasopressors (with norepinephrine equivalent doses calculated to quantify overall vasoactive exposure and to identify patients receiving ≥2 agents) [21], renal replacement therapy, opioids, insulin use, and sedation therapy (with total 24-hour doses and duration). Ventilation was defined as machine ventilation support, including high-flow nasal cannula (HFNC), non-invasive ventilation (NIV), invasive mechanical ventilation (IMV), or tracheostomy ventilation, initiated within the first 24 hours after ICU admission, representing acute respiratory failure at ICU entry. RRT use in this study reflected acute kidney injury secondary to sepsis, rather than preexisting renal failure. The mean values of vital signs and the worst laboratory parameters within the first 24 hours after ICU admission were analyzed, as the latter reflected the peak severity of the acute physiological response, consistent with their use in ICU severity scoring systems such as SAPS II [22] and APACHE IV [23]. Sedation therapy was defined as administering propofol, midazolam, dexmedetomidine, or any combination thereof during the initial 24-hour ICU stay, with detailed records of drug combinations documented. Sedation was initiated primarily for facilitating mechanical ventilation tolerance or managing severe agitation that could not be controlled by non-pharmacologic interventions, consistent with standard ICU practice. Explicit clinical indications for sedation were not available in the MIMIC-IV database. The primary outcome measure was one-year all-cause mortality, defined as death due to any cause within one year following ICU admission. The second outcome was ICU mortality, defined as death due to any cause during ICU stay. Follow-up time for each participant was calculated from 24 hours after the first ICU admission to the date of death or one-year post-ICU admission.

### 2.4 Statistical analysis

The distribution of continuous baseline variables was examined using histograms and kernel density plots to determine whether data were approximately normally distributed. The participants’ baseline characteristics were presented as means ± standard deviations for normally distributed variables, medians with interquartile ranges (IQR) for continuous variables, and counts with percentages for categorical variables. We analyzed these characteristics across sedative and non-sedative use groups using one-way ANOVA for normally distributed data, the Kruskal-Wallis H test for skewed distributions, and the chi-square or Fisher’s exact test for categorical variables. Univariate and multivariate Cox proportional hazards (PH) regression models examined the relationship between sedation therapy and one-year all-cause mortality across five distinct models. Furthermore, we used the Schoenfeld residual test to verify the PH assumption in the Cox analysis [24]. When the PH assumption was not satisfied, we applied an alternative analytical approach based on restricted mean survival time (RMST) to explore the association between sedation therapy and one-year all-cause mortality. Model 1 was an unadjusted model with no covariates. Model 2 was refined to include confounders identified from prior literature and clinical relevance. All candidate variables were tested for multicollinearity, and those with a variance inflation factor (VIF) < 5 were retained. Model 3 was built upon Model 2 and included multiple imputations for missing values, utilizing five replications. To reduce potential confounding bias, we generated a propensity score to estimate the likelihood of one-year all-cause mortality for patients through Cox PH regression. We applied a 1:1 nearest neighbor matching algorithm with a caliper width of 0.2 and without replacement. The variables selected to generate the propensity score matched those in Model 2. The degree of propensity score matching (PSM) was assessed using a standardized mean difference (SMD), with < 0.1 regarded as an acceptable threshold. When the SMD was still ≥ 0.1, a doubly robust estimation combined a multivariate Cox regression model with a propensity score model to estimate sedation therapy’s association and causal effect on one-year all-cause mortality [25]. Furthermore, we analyzed overlap weight (OW) to address potential confounding. The results from PSM and OW, exploring the relationship between sedation therapy and one-year all-cause mortality, were designated as Model 4 and Model 5, respectively. Subgroup analyses were conducted using a stratified Cox PH regression model. We first converted the continuous variable into a categorical one based on the clinical cutoff point and then performed an interaction test. The effect modification of subgroup indicators was assessed using the likelihood ratio test. Kaplan–Meier survival curves were created to compare one-year all-cause mortality between sedative and non-sedative use groups and among different combinations of sedative therapy groups. Patients who were not allowed to follow up were censored then. We calculated E-values to explore the potential for unmeasured confounding between sedative use and one-year all-cause mortality. A priori statistical power calculations were not conducted because the sample size was based entirely on available data. The R software (version 4.2.2; R Foundation for Statistical Computing; http://www.Rproject.org) and Free Statistics software (version 2.1; Beijing Free Clinical Medical Technology Co., Ltd.) were utilized for the analyses. A two-sided *P*-value < 0.05 indicated statistical significance in all analyses.

## 3 Results

### 3.1 Baseline characteristics

**Fig 1:**
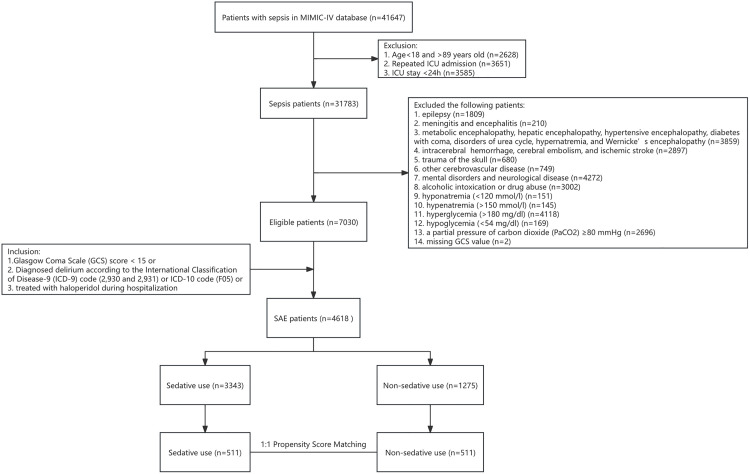
Flowchart illustrating the screening and enrollment process for study participants.

We included 41,647 patients from the MIMIC-IV database who met the SEPSIS-3 diagnostic criteria (Fig 1). The exclusions were as follows: 1. individuals under 18 or over 89 years of age (n = 2,628); 2. repeated ICU admissions (n = 3,651); and 3. ICU stays of less than 24 hours (n = 3,585). This process resulted in 31,783 patients diagnosed with sepsis. Subsequently, using the appropriate ICD-9 or ICD-10 codes, we removed the following conditions: 1. epilepsy (n = 1,809); 2. meningitis and encephalitis (n = 210); 3. metabolic encephalopathy, hepatic encephalopathy, hypertensive encephalopathy, diabetes with coma, urea cycle disorders, hypernatremia, and Wernicke’s encephalopathy (n = 3,859); 4. intracerebral hemorrhage, cerebral embolism, and ischemic stroke (n = 2,897); 5. traumatic brain injury (n = 680); 6. other cerebrovascular diseases (n = 749); 7. mental disorders and neurological diseases (n = 4,272); 8. alcohol intoxication or drug abuse (n = 3,002); 9. hyponatremia (below 120 mmol/L) (n = 151); 10. hypernatremia (>150 mmol/L) (n = 145); 11. hyperglycemia (above 180 mg/dL) (n = 4,118); 12. hypoglycemia (below 54 mg/dL) (n = 169); and 13. a PaCO_2_ pressure of 80 mmHg or greater (n = 2,696). After applying the exclusion criteria, 7,030 patients remained eligible. According to the SAE diagnostic criteria outlined in the methodology section, two patients without a GCS score were excluded for not meeting the criteria. This led to a final analysis of 4,618 SAE patients, including 3,343 in the sedative and 1,275 in the non-sedative use groups. Finally, we performed PSM between sedative and non-sedative patients in a 1:1 ratio, resulting in 511 matched pairs for further analysis.

Relevant variables were collected from various categories, including demographic characteristics, vital signs, laboratory tests, infection sites, pathogenic microorganisms, comorbidities, scores, and treatments within the original cohorts (Table 1). Intergroup comparisons revealed significant differences in baseline characteristics for most variables in the original cohort (*P* < 0.05), except for race, mean blood pressure, potassium, PTT, intestinal infections, skin and soft tissue infections, *E. coli*, *Staphylococcus aureus*, and diabetes. The missing data for the original cohort variables were as follows: heart rate, mean blood pressure, and SpO_2_ (9 cases, 0.2%); systolic and diastolic blood pressure (25 cases, 0.5%); respiratory rate (10 cases, 0.2%); body temperature (420 cases, 9.1%); BUN, hemoglobin, platelets, and WBC (1 case, < 0.1%); lactate (456 cases, 9.9%); potassium (2 cases, < 0.1%); INR and PT (236 cases, 5.1%); and PTT (251 cases, 5.4%). No missing data were identified for the remaining variables. After performing multiple imputations for the missing data, PSM was conducted. Variables included for matching were selected based on a variance inflation factor (VIF) of less than 5. The final set excluded DBP, MBP, INR, and PT due to collinearity. Covariate balance was well achieved between the two groups, with standardized mean differences (SMDs) below 0.1 for all variables except first-day ventilation (S1A Fig). As shown in S11 Table, in the original cohort, the sedative-use group had higher norepinephrine equivalent doses and more frequent use of multiple vasopressors than the non-sedative group (all P < 0.001). After propensity score matching, vasopressor use and norepinephrine equivalent doses were comparable between groups (P = 0.753 and P = 0.917, respectively). The sedative-use group received higher total doses of propofol, midazolam, and dexmedetomidine and had longer sedation durations than the non-sedative group (all P < 0.05), confirming consistent exposure classification. Additional stratified summaries are provided in S12–S13 Tables. In S12 Table, patients with day-1 ventilation (HFNC/NIV/IMV/tracheostomy) showed higher illness severity and treatment intensity in the original cohort (e.g., SOFA and SAPS II, vasopressor use, lactate/PaCO₂ ↑ , PaO₂↓), whereas these differences were broadly balanced between sedative and non-sedative groups after matching. In S13 Table, patients receiving RRT within 24 h had markedly worse renal indices and higher severity pre-match (e.g., creatinine, SOFA, SAPS II), with most variables remaining comparable between sedative and non-sedative groups in the matched cohort (small residual differences noted). As shown in S15 Table, in the original cohort, the median ICU stay was 3.4 days (IQR 1.9–6.9), and ICU mortality was 10.4%, being lower among sedated than non-sedated patients (8.5% vs. 15.3%, P < 0.001). In matching cohort, ICU length of stay was slightly longer in the sedative group (4.0 vs. 3.1 days, P = 0.001), whereas ICU mortality was comparable between groups (12.3% vs. 13.7%, P = 0.515).

**Table 1:** Characteristics of patients with SAE.

Variables	Original cohort				Matched cohort			
	Total (n = 4618)	Sedative use (n = 3343)	Non-sedative use (n = 1275)	*P*-value	Total (n = 1022)	Sedative use (n = 511)	Non-sedative use (n = 511)	*P*-value
**Demographic characteristics**								
Male	2762 (59.8)	2083 (62.3)	679 (53.3)	< 0.001	573 (56.1)	284 (55.6)	289 (56.6)	0.753
Age, years	66.8 ± 14.7	66.3 ± 14.8	68.0 ± 14.6	< 0.001	66.9 ± 14.9	67.0 ± 14.9	66.7 ± 14.9	0.711
White	3276 (70.9)	2367 (70.8)	909 (71.3)	0.743	720 (70.5)	359 (70.3)	361 (70.6)	0.891
Recent surgery before ICU admission, n (%)	3875 (83.9)	3126 (93.5)	749 (58.7)	< 0.001	825 (80.7)	411 (80.4)	414 (81)	0.812
**Vital signs, Mean ± SD**								
Heart rate, beats per minute	86.8 ± 15.2	85.4 ± 14.3	90.2 ± 16.8	< 0.001	87.8 ± 16.1	88.0 ± 16.0	87.6 ± 16.2	0.702
Systolic blood pressure, mmHg	113.5 ± 13.0	113.1 ± 11.3	114.5 ± 16.6	< 0.001	114.4 ± 14.6	114.3 ± 13.4	114.5 ± 15.7	0.819
Diastolic blood pressure, mmHg	59.4 ± 9.3	58.7 ± 8.5	61.1 ± 10.9	< 0.001	60.1 ± 9.8	60.3 ± 9.3	59.8 ± 10.3	0.412
Mean blood pressure, mmHg	75.3 ± 8.9	75.2 ± 7.9	75.7 ± 11.0	0.068	75.8 ± 9.8	75.9 ± 9.0	75.7 ± 10.6	0.709
Respiratory rate, breaths per minute	19.2 ± 4.1	18.6 ± 3.6	20.8 ± 4.8	< 0.001	19.6 ± 4.1	19.7 ± 4.0	19.5 ± 4.1	0.417
Body temperature, °C	36.9 ± 0.6	36.9 ± 0.6	36.8 ± 0.5	< 0.001	36.9 ± 0.6	36.9 ± 0.6	36.9 ± 0.5	0.873
SpO_2_, %	97.3 ± 1.9	97.7 ± 1.7	96.3 ± 2.1	< 0.001	96.9 ± 2.0	96.9 ± 2.2	97.0 ± 1.8	0.73
**Laboratory tests**								
BUN, mg/dL	21.0 (15.0, 34.0)	19.0 (14.0, 29.0)	27.0 (17.0, 47.0)	< 0.001	23.0 (15.0, 40.0)	22.0 (16.0, 37.0)	23.0 (15.0, 41.0)	0.497
Creatinine, mg/dL	1.0 (0.8, 1.6)	1.0 (0.8, 1.5)	1.2 (0.8, 2.1)	< 0.001	1.1 (0.8, 1.8)	1.0 (0.8, 1.8)	1.1 (0.8, 1.8)	0.449
Glucose, mg/dL	129.6 ± 25.6	128.7 ± 25.5	132.0 ± 25.8	< 0.001	133.7 ± 25.4	133.7 ± 25.9	133.7 ± 25.0	0.956
PaO_2_, mmHg	102.6 ± 53.8	105.2 ± 52.0	95.9 ± 57.7	< 0.001	107.5 ± 67.1	106.9 ± 67.9	108.1 ± 66.4	0.761
PaCO_2_, mmHg	46.7 ± 9.8	48.2 ± 8.7	42.8 ± 11.3	< 0.001	45.4 ± 10.7	45.4 ± 9.5	45.4 ± 11.7	0.988
Lactate, mmol/L	2.2 (1.5, 3.3)	2.4 (1.7, 3.5)	1.6 (1.1, 2.5)	< 0.001	1.8 (1.2, 2.6)	1.8 (1.2, 2.6)	1.8 (1.2, 2.6)	0.813
Sodium, mmol/L	136.9 ± 4.0	137.2 ± 3.6	136.1 ± 4.9	< 0.001	136.7 ± 4.3	136.8 ± 4.3	136.6 ± 4.4	0.357
Potassium, mmol/L	4.6 ± 0.8	4.6 ± 0.8	4.6 ± 0.9	0.219	4.5 ± 0.7	4.5 ± 0.7	4.5 ± 0.7	0.756
Hemoglobin, g/dL	9.5 ± 1.9	9.4 ± 1.8	9.7 ± 2.0	< 0.001	9.5 ± 2.0	9.5 ± 1.9	9.5 ± 2.0	0.992
Platelets, x10^9^/L	176.7 ± 104.7	165.4 ± 96.0	206.6 ± 119.5	< 0.001	198.2 ± 118.4	198.4 ± 122.2	197.9 ± 114.7	0.945
WBC, x10^9^/L	15.5 ± 8.8	15.9 ± 8.7	14.6 ± 9.1	< 0.001	13.6 (9.7, 18.3)	13.6 (9.9, 17.8)	13.3 (9.4, 18.8)	0.831
INR	1.6 ± 1.0	1.6 ± 0.9	1.8 ± 1.2	< 0.001	1.3 (1.2, 1.6)	1.3 (1.2, 1.6)	1.3 (1.2, 1.7)	0.538
PT, seconds	17.8 ± 10.2	17.3 ± 9.1	19.3 ± 12.7	< 0.001	14.9 (13.3, 17.9)	15.0 (13.6, 17.8)	14.7 (13.2, 18.2)	0.45
PTT, seconds	44.2 ± 28.1	43.7 ± 27.2	45.4 ± 30.5	0.074	33.1 (28.5, 43.7)	32.9 (28.4, 42.6)	33.3 (28.8, 44.4)	0.637
**Site of infection, n (%)**								
Intestinal infection	69 (1.5)	52 (1.6)	17 (1.3)	0.578	15 (1.5)	7 (1.4)	8 (1.6)	0.795
Catheter infection	75 (1.6)	42 (1.3)	33 (2.6)	0.001	18 (1.8)	8 (1.6)	10 (2)	0.634
Skin and soft tissue infection	4 (0.1)	1 (0)	3 (0.2)	0.067	2 (0.2)	1 (0.2)	1 (0.2)	1
Urinary infection	490 (10.6)	307 (9.2)	183 (14.4)	< 0.001	117 (11.4)	62 (12.1)	55 (10.8)	0.492
Pulmonary infection	890 (19.3)	589 (17.6)	301 (23.6)	< 0.001	226 (22.1)	120 (23.5)	106 (20.7)	0.291
**Pathogenic microorganisms, n (%)**								
Acinetobacter baumannii	6 (0.1)	1 (0)	5 (0.4)	0.007	1 (0.1)	0 (0)	1 (0.2)	1
E. coli	153 (3.3)	103 (3.1)	50 (3.9)	0.154	37 (3.6)	20 (3.9)	17 (3.3)	0.615
Staphylococcus aureus	435 (9.4)	320 (9.6)	115 (9)	0.565	102 (10.0)	54 (10.6)	48 (9.4)	0.531
Klebsiella pneumoniae	73 (1.6)	37 (1.1)	36 (2.8)	< 0.001	24 (2.3)	14 (2.7)	10 (2)	0.409
Pseudomonas aeruginosa	99 (2.1)	48 (1.4)	51 (4)	< 0.001	40 (3.9)	21 (4.1)	19 (3.7)	0.747
**Comorbidity, n (%)**								
Hypertension	3151 (68.2)	2310 (69.1)	841 (66)	0.041	664 (65.0)	335 (65.6)	329 (64.4)	0.694
Congestive heart failure	1400 (30.3)	927 (27.7)	473 (37.1)	< 0.001	316 (30.9)	163 (31.9)	153 (29.9)	0.499
Chronic pulmonary disease	1205 (26.1)	803 (24)	402 (31.5)	< 0.001	299 (29.3)	155 (30.3)	144 (28.2)	0.449
Diabetes	1173 (25.4)	846 (25.3)	327 (25.6)	0.864	235 (23.0)	115 (22.5)	120 (23.5)	0.74
Renal disease	1003 (21.7)	628 (18.8)	375 (29.4)	< 0.001	239 (23.4)	124 (24.3)	115 (22.5)	0.506
**Scores**								
Charlson comorbidity index	5.6 ± 2.8	5.3 ± 2.6	6.5 ± 3.0	< 0.001	5.9 ± 2.9	6.0 ± 2.9	5.9 ± 2.9	0.622
SOFA	3.9 ± 2.1	4.0 ± 2.1	3.4 ± 1.8	< 0.001	3.6 ± 1.9	3.6 ± 1.9	3.6 ± 2.0	0.832
SAPSII	41.3 ± 14.1	42.4 ± 14.4	38.6 ± 13.0	< 0.001	39.4 ± 14.1	40.1 ± 14.7	38.8 ± 13.6	0.145
**Treatments, n (%)**								
First day insulin use	2928 (63.4)	2468 (73.8)	460 (36.1)	< 0.001	487 (47.7)	237 (46.4)	250 (48.9)	0.416
First day ventilation	2627 (56.9)	2354 (70.4)	273 (21.4)	< 0.001	373 (36.5)	204 (39.9)	169 (33.1)	0.023
First day vasopressor	2587 (56.0)	2241 (67)	346 (27.1)	< 0.001	400 (39.1)	202 (39.5)	198 (38.7)	0.798
First day renal replacement therapy	245 (5.3)	147 (4.4)	98 (7.7)	< 0.001	76 (7.4)	36 (7)	40 (7.8)	0.633
First day opioids use	3861 (83.6)	3179 (95.1)	682 (53.5)	< 0.001	813 (79.5)	402 (78.7)	411 (80.4)	0.485

Data are presented as mean±SD, median (Q1–Q3), or N (%).

**Fig 2:**
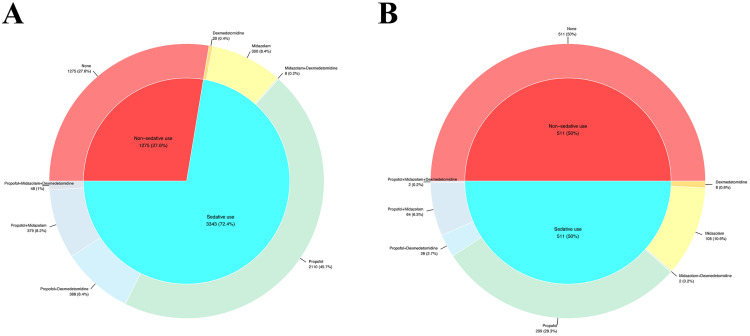
Sedatives. (A) Original cohort; (B) Matched cohort.

Fig 2 illustrates the specific combinations of sedatives utilized. The most frequently administered single sedative was propofol, followed by midazolam. Among the combinations, propofol combined with dexmedetomidine was the most used, while the combination of propofol with midazolam ranked second.

### 3.2 Relationship between sedative use and the one-year all-cause mortality of patients with SAE

The Schoenfeld residual test indicated that, except for the dexmedetomidine monotherapy group, all other groups satisfied the PH assumption required for Cox regression analysis (S9 Table). As shown in Table 2, the univariate Cox regression analysis (Model 1) indicated that the use of any type of sedatives, including propofol, midazolam, or dexmedetomidine, was associated with a 63% lower odds of one-year all-cause mortality in patients with SAE compared to non-sedative use, with an HR of 0.37 (95% CI 0.33–0.42). In the multivariate Cox regression analysis, variables showing collinearity (DBP, MBP, INR, and PT) were excluded, the HR was 0.62 (95% CI 0.52–0.76) in Model 2. After imputing missing values in Model 3, the HR remained at 0.67 (95% CI 0.57–0.79). After applying PSM combined with multivariable adjustment (Model 4) and weighted Overlap (Model 5), the HR was 0.69 (95% CI 0.57–0.85) and 0.72 (95% CI 0.57–0.92), respectively. Across the five aforementioned models, compared with SAE patients receiving no sedation, administration of propofol alone was significantly associated with reduced 1-year all-cause mortality in SAE patients (*P* < 0.05). For the dexmedetomidine group, which did not satisfy the PH assumption, the difference in RMST over the one-year follow-up period, compared to patients without sedation, was 68.678 (4.692 ~ 132.664) days (S10 Table).

**Table 2:** Association between sedative use and the one-year all-cause mortality of patients with SAE.

Sedatives	One-year all-cause mortality									
	Model 1		Model 2		Model 3		Model 4		Model 5	
	HR (95%CI)	*P*-value	HR (95%CI)	*P*-value	HR (95%CI)	*P*-value	HR (95%CI)	*P*-value	HR (95%CI)	*P*-value
Non-sedatives	1	Ref	1	Ref	1	Ref	1	Ref	1	Ref
Sedative use	0.37 (0.33 ~ 0.42)	**<0.001**	0.62 (0.52 ~ 0.76)	**<0.001**	0.67 (0.57 ~ 0.79)	**<0.001**	0.69 (0.57 ~ 0.85)	**<0.001**	0.72 (0.57 ~ 0.92)	**<0.001**
Propofol	0.26 (0.23 ~ 0.3)	**<0.001**	0.51 (0.4 ~ 0.65)	**<0.001**	0.54 (0.44 ~ 0.67)	**<0.001**	0.68 (0.53 ~ 0.86)	0.002	0.67 (0.5 ~ 0.9)	**<0.001**
Midazolam	1.14 (0.97 ~ 1.34)	0.103	0.97 (0.75 ~ 1.25)	0.811	0.98 (0.79 ~ 1.22)	0.885	0.87 (0.67 ~ 1.15)	0.336	0.94 (0.68 ~ 1.31)	0.598
Propofol+Midazolam	0.65 (0.54 ~ 0.79)	<0.001	0.79 (0.58 ~ 1.06)	0.114	0.69 (0.53 ~ 0.9)	**0.006**	0.65 (0.45 ~ 0.93)	**0.018**	0.75 (0.49 ~ 1.15)	0.061
Propofol+Dexmedetomidine	0.16 (0.12 ~ 0.23)	<0.001	0.29 (0.18 ~ 0.47)	**<0.001**	0.26 (0.17 ~ 0.39)	**<0.001**	0.62 (0.36 ~ 1.07)	0.086	0.64 (0.33 ~ 1.24)	0.07
Propofol+Midazolam+Dexmedetomidine	0.38 (0.2 ~ 0.71)	0.002	0.56 (0.26 ~ 1.2)	0.139	0.53 (0.27 ~ 1.05)	0.068	0.98 (0.23 ~ 4.27)	0.98	0.86 (0.24 ~ 3.07)	0.755

Model 1: Unadjusted model with no covariates.

Model 2: Covariate screening+Multivariable adjusted. All candidate covariates shown in Table 1 were tested for multicollinearity. Variables with a variance inflation factor (VIF) < 5 were considered acceptable and entered into the final model, whereas DBP, MBP, INR, and PT were removed owing to excessive collinearity.

Model 3: Multiple imputations+Model 2.

Model 4: Propensity Score Matched. A doubly robust method incorporating unbalanced covariates, specifically propensity score matching combined with multivariable adjustment, was employed when necessary. Sedative use was adjusted for first-day ventilation. Propofol was adjusted for SOFA.

Model 5: Overlap weight.

### 3.3 Prognostic analyses of patients with SAE

**Fig 3:**
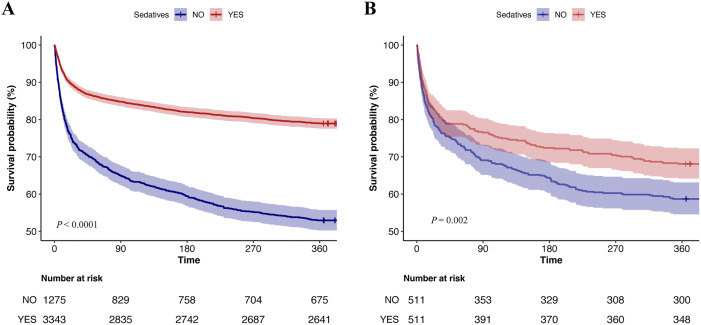
Kaplan-Meier survival curves between the sedative use and non-sedative use groups. (A) Original cohort; (B) Matched cohort.

**Fig 4:**
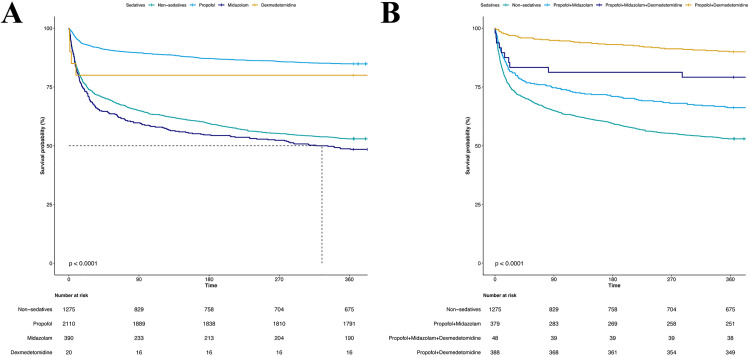
Kaplan-Meier survival curves between the different sedatives and non-sedative groups. (A) monotherapy; (B) combinations.

Compared to patients with SAE who did not receive sedatives, the one-year all-cause mortality rate was lower in those treated with sedatives, regardless of whether they were in the original or matched cohort (log-rank test *P* < 0.05, Fig 3). Univariate analysis indicated propofol, whether used alone or combined with dexmedetomidine, was strongly associated with long-term survival, as demonstrated by the Kaplan–Meier curves (log-rank test *P* < 0.0001, Fig 4).

### 3.4 Subgroup analysis

**Fig 5:**
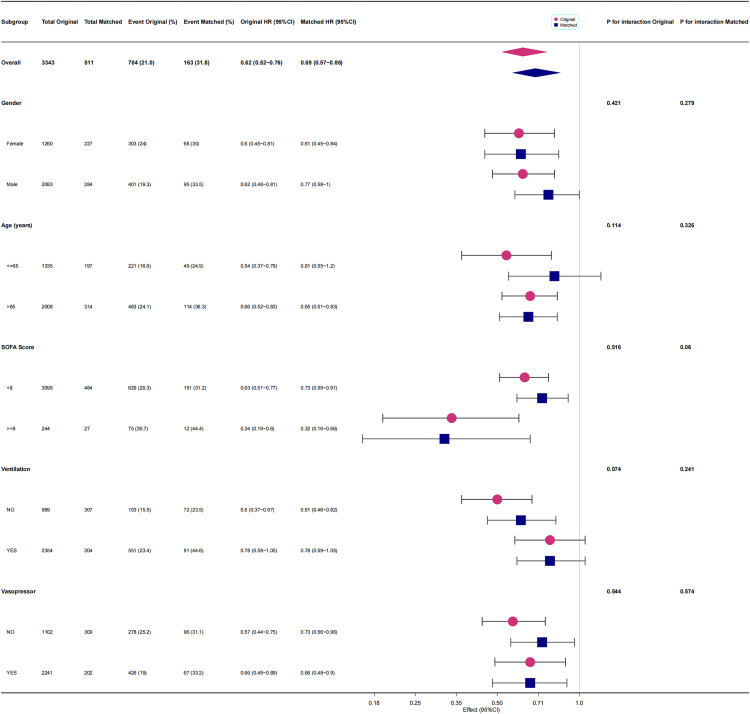
The effect size of sedative use on one-year all-cause mortality of patients with SAE in each subgroup.

**Fig 6:**
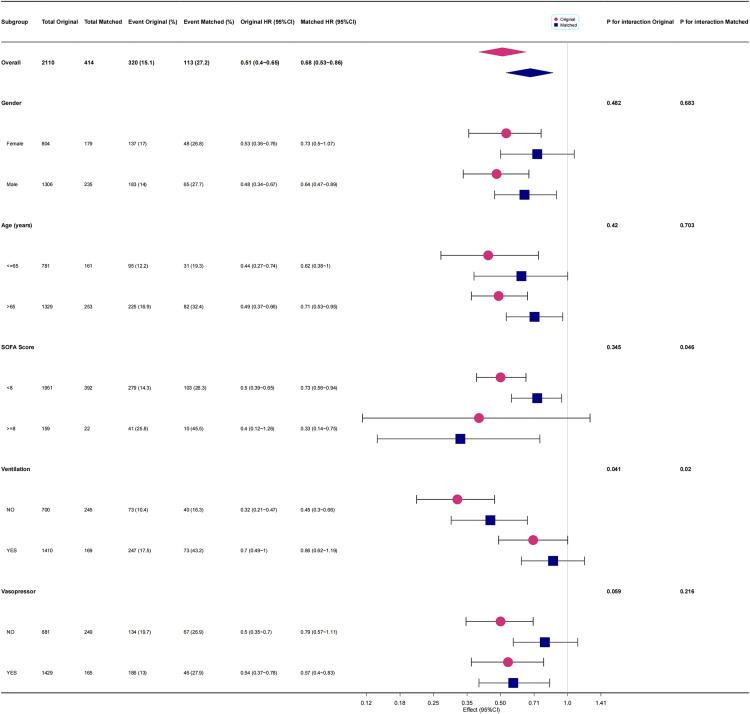
The effect size of propofol monotherapy on one-year all-cause mortality of patients with SAE in each subgroup.

Figs 5 and 6 presented the results of a subgroup analysis conducted using multivariate Cox regression model 2 and propensity score-matched model 4. The analysis indicated that in both the original and matched cohorts, sedation therapy with any type—predominantly with propofol monotherapy—was associated with HR values of less than 1 and *P* values below 0.05 across most subgroups stratified by gender, age, and vasopressor use.

In the SOFA score–stratified analysis, sedation therapy with any agent—or with propofol monotherapy—was generally associated with hazard ratios below 1 and P values < 0.05 across all strata. Among patients with higher SOFA scores (≥8), the observed hazard ratios were even lower than those in the lower SOFA group (<8). Moreover, in the propofol-only matched cohort, a significant interaction between sedation and SOFA score was detected (P for interaction < 0.05) (Fig 6).

In the ventilation-stratified analysis, sedation therapy with any agent or propofol monotherapy was associated with hazard ratios below 1 in both the original and matched cohorts; however, the P values were greater than 0.05 across ventilation subgroups. Notably, when analyzing propofol monotherapy, a significant interaction between sedation and ventilation support was observed in both the original and matched cohorts (P for interaction < 0.05) (Fig 6).

### 3.5 Sensitivity analysis

As shown in S2 and S3 Figs, the E-values and the lower CI limit for sedative use and propofol alone, were 2.13 (1.71) and 2.56 (2.03) in the original cohort, 1.91 (1.48) and 1.94 (1.46) in the matched cohort, respectively.

## 4 Discussion

Compared to SAE patients not receiving sedation, our study found that administering of any sedative agent—predominantly propofol monotherapy—significantly reduced one-year all-cause mortality in patients with SAE (*P* < 0.05). The subgroup analysis indicated that among patients who received ventilation support on the first day of ICU admission, the one-year all-cause mortality significantly increased with sedatives or propofol use, with a notable interaction observed between propofol use and ventilation support.

Reports indicated that a wide range of ICU patients (12%–80%) experience anxiety [26,27]. In our study, sedation therapy for anxiety or agitation was administered to 72.4% of the overall SAE patient population. Across the five aforementioned models, administering propofol alone, compared to no sedation, was consistently associated with a significant reduction in one-year all-cause mortality among SAE patients (P < 0.05). This suggested that our study’s findings were robust and reliable. Furthermore, across the five previously mentioned models, Models 2 and 3 demonstrated a statistically significant association (P < 0.001), whereas in Models 4 and 5, no significant difference was observed in one-year all-cause mortality between SAE patients receiving propofol combined with dexmedetomidine and those without sedation (*P* > 0.05). This lack of significance is most likely attributable to the limited sample size in the matched analysis. Given the high mortality rate (33%–66%) [28,29] of propofol infusion syndrome, a rare complication (<1%) [30] associated with high-dose [>4 mg/(kg·h) or >67 μg/(kg·min)] and prolonged (>48 h) propofol sedation [31,32], our findings suggested that expanding the sample size and further investigating the potential role of combining propofol with dexmedetomidine to reduce total propofol dosage may represent a promising direction for future research. For the dexmedetomidine group, which did not satisfy the PH assumption, the difference in RMST over the one-year follow-up period, compared to patients without sedation, was 68.678 (4.692 ~ 132.664) days. The violation of the PH assumption might be attributable to the relatively small sample size in the dexmedetomidine monotherapy group (n = 20). Nevertheless, the encouraging RMST results highlighted the need for further investigation with a larger sample size. The Kaplan–Meier curves illustrating the prognostic analyses of patients with SAE were consistent with these conclusions.

Figs 5 and 6 indicated that in both the original and matched cohorts, sedation therapy—predominantly with propofol monotherapy—was a protective factor against one-year all-cause mortality across subgroups stratified by gender, age, and vasopressor support. The *P*-value for the interaction between propofol use and SOFA score in the matched cohort was 0.046. This finding might reflect that patients with higher SOFA scores (≥8 points), indicating greater disease severity, experienced higher mortality and thus gained more benefit from sedation therapy. However, caution was warranted in interpreting this result, as this phenomenon was not observed in the original cohort, and the *P*-value was obtained after multiple tests and was close to 0.05. In the subgroup of patients receiving ventilation support on the first ICU day, the use of ventilation support appeared to attenuate the protective effect of sedation therapy on one-year all-cause mortality among SAE patients (Fig 5). Similarly, for those treated with propofol monotherapy, ventilation support also diminished the protective association with survival. These findings suggest that ventilation support may act as a risk factor for increased mortality in sedated SAE patients. Although higher sedative doses may indicate greater illness severity or hemodynamic instability, as detailed in S11 Table, our adjusted analyses—including SOFA score, SAPS II, and norepinephrine-equivalent dose—showed that sedation use remained independently associated with lower one-year mortality. After propensity score matching, circulatory parameters were well balanced between groups, suggesting that the observed protective effect of early sedation was not merely a reflection of baseline severity. Nevertheless, as an observational study, residual confounding by indication cannot be fully excluded, and the higher mortality observed among ventilated patients receiving propofol monotherapy may partly reflect their greater baseline illness burden rather than a direct pharmacologic effect.

The sensitivity analysis showed that the E-values and their corresponding lower CI limits for both sedative use and propofol monotherapy were approximately or above 2 in the original and matched cohorts. This finding suggested that substantial unmeasured confounding would be necessary to nullify the observed HRs, thereby supporting the robustness and stability of the current associations [33].

Our study had several limitations. Firstly, as a retrospective analysis and given the absence of a gold standard for SAE diagnosis, we defined SAE using previously established research criteria that primarily relied on subjective evaluation and diagnosis by exclusion. SAE represents a spectrum of sepsis-related brain dysfunction, in which delirium constitutes the most common clinical manifestation. According to current consensus and prior epidemiologic studies, SAE encompasses both acute confusional states and decreased levels of consciousness in the absence of direct central nervous system infection or structural injury. In this study, delirium was therefore considered an integral part of SAE rather than a distinct entity, and our diagnostic criteria—including GCS < 15 on the first ICU day, delirium diagnosis codes, or haloperidol use in septic patients—were designed to capture this full clinical continuum. This operational definition aligns with international consensus frameworks [2,4], which define SAE as diffuse brain dysfunction secondary to sepsis without direct CNS infection or structural injury, ensuring comparability with global SAE research. Although this approach might have introduced selection bias, future studies should incorporate more objective indicators, such as cranial ultrasound, brain MRI, and bedside EEG monitoring, to enhance the accuracy and timeliness of SAE identification. Additionally, a small subset of patients (n = 63) had a GCS score < 15 prior to ICU admission and thus may have developed SAE before ICU entry. However, as our study focused on long-term outcomes among patients with SAE irrespective of onset timing, this factor was unlikely to materially affect the observed associations. Secondly, due to the limited sample size in the current MIMIC-IV v3.1 database—particularly for the dexmedetomidine monotherapy group—even preliminary analyses suggesting a pronounced protective effect on one-year all-cause mortality in patients with SAE could not be examined more thoroughly, constraining further in-depth investigation. Thirdly, our study focused exclusively on the association between sedation therapy and one-year all-cause mortality; however, given the inherent limitations of an observational design, the existence of a causal relationship remained uncertain. Furthermore, the MIMIC-IV database provides information on long-term mortality but lacks follow-up data on post-discharge neurological or cognitive outcomes. Therefore, correlations between SAE and subsequent neurocognitive symptoms could not be evaluated, and prospective studies with standardized long-term assessments are warranted. Lastly, propofol lipid emulsions may favor microbial growth and require aseptic handling; clinicians should interpret the protective findings with caution.

In conclusion, our study found that compared to patients with SAE who did not receive sedation, administering propofol significantly reduced one-year all-cause mortality in patients with SAE. Among patients undergoing ventilation support on the first day of ICU admission, the interaction between propofol therapy and ventilation support significantly affected the one-year all-cause mortality of patients with SAE. Our study offers valuable insights for clinical practice, suggesting that propofol monotherapy can be chosen when sedation is needed for patients with SAE. Moreover, early propofol use may confer greater survival benefits among patients with higher SOFA scores (≥8), reflecting more severe illness. However, these findings need further validation in prospective trials.

## Supporting information

S1 Table Exclude patients with trauma of brain from the MIMIC-IV database according to ICD-codes. (DOCX)

S2 Table Exclude patients with intracerebral hemorrhage, cerebral embolism and ischemic stroke disease from the MIMIC-IV database according to ICD-codes. (DOCX)

S3 Table Exclude patients with meningitis and encephalitis disease from the MIMIC-IV database according to ICD-codes. (DOCX)

S4 Table Exclude patients with epilepsy disease from the MIMIC-IV database according to ICDcodes. (DOCX)

S5 Table Exclude patients with other cerebrovascular disease from the MIMIC-IV database according to ICD-codes. (DOCX)

S6 Table Exclude patients with mental disorders and and neurological disease from the MIMIC-IV database according to ICD-codes. (DOCX)

S7 Table Exclude patients with alcoholic intoxication or or drug abuse from the MIMIC-IV database according to ICD-codes. (DOCX)

S8 Table Exclude patients with metabolic encephalopathy, hepatic encephalopathy, hypertensive encephalopathy, diabetes with coma, disorders of urea cycle, hypernatremia, wrnicke’s encephalopathy from the MIMIC-IV database according to ICD-codes. (DOCX)

S9 Table Assessing proportional hazards. (DOCX)

S10 Table Comparing restricted mean survival time of dexmedetomidine with non-sedative. (DOCX)

S11 Table Vasopressor and sedative in the original and matched cohorts. (DOCX)

S12 Table Baseline characteristics stratified by day-1 ventilation status in the original and matched cohorts. (DOCX)

S13 Table Baseline characteristics stratified by renal replacement therapy (RRT) within the first 24 hours in the original and matched cohorts. (DOCX)

S14 Table Respiratory parameters and ARDS severity of patients receiving mechanical ventilation (MV) within the first 24 hours after ICU admission. (DOCX)

S15 Table ICU length of stay and mortality in the original and matched cohorts. (DOCX)

S1 Fig Standardized mean difference of variables before matched, after PSM and after weighted OW. (A) Sedative use; (B) Propofol. (DOCX)

S2 Fig E-value of sedative use. (A) Original cohort; (B) Matched cohort. (DOCX)

S3 Fig E-value of propofol. (A) Original cohort; (B) Matched cohort. (DOCX)

S4 Fig Distribution of representative continuous variables between sedative-use and non–sedative-use groups. (A) Respiratory rate; (B) SAPS II; (C) Blood urea nitrogen (BUN); (D) Lactate. (DOCX)
