# Supplementary material for: Propofol provides a significant survival advantage in sepsis-associated encephalopathy: A retrospective cohort study investigating one-year all-cause mortality
Source: PLoS One. 2026 Feb 5;21(2):e0340371. doi: 10.1371/journal.pone.0340371 (PMC12875438; doi:10.1371/journal.pone.0340371)
Supplement: S1 Table — (DOCX) [file pone.0340371.s001.docx]

Supporting Information

# S1 Table. Exclude patients with trauma of brain from the MIMIC-IV database according to ICD-codes

| ICD-code | ICD | Description |
| --- | --- | --- |
| 80016 | ICD9 | Closed fracture of vault of skull with cerebral laceration and contusion, with loss of consciousness of unspecified duration |
| 80019 | ICD9 | Closed fracture of vault of skull with cerebral laceration and contusion, with concussion, unspecified |
| 80020 | ICD9 | Closed fracture of vault of skull with subarachnoid, subdural, and extradural hemorrhage, unspecified state of consciousness |
| 80021 | ICD9 | Closed fracture of vault of skull with subarachnoid, subdural, and extradural hemorrhage, with no loss of consciousness |
| 80022 | ICD9 | Closed fracture of vault of skull with subarachnoid, subdural, and extradural hemorrhage, with brief [less than one hour] loss of consciousness |
| 80023 | ICD9 | Closed fracture of vault of skull with subarachnoid, subdural, and extradural hemorrhage, with moderate [1-24 hours] loss of consciousness |
| 80024 | ICD9 | Closed fracture of vault of skull with subarachnoid, subdural, and extradural hemorrhage, with prolonged [more than 24 hours] loss of consciousness and return to pre-existing conscious level |
| 80025 | ICD9 | Closed fracture of vault of skull with subarachnoid, subdural, and extradural hemorrhage, with prolonged [more than 24 hours] loss of consciousness, without return to pre-existing conscious level |
| 80026 | ICD9 | Closed fracture of vault of skull with subarachnoid, subdural, and extradural hemorrhage, with loss of consciousness of unspecified duration |
| 80029 | ICD9 | Closed fracture of vault of skull with subarachnoid, subdural, and extradural hemorrhage, with concussion, unspecified |
| 80030 | ICD9 | Closed fracture of vault of skull with other and unspecified intracranial hemorrhage, unspecified state of consciousness |
| 80031 | ICD9 | Closed fracture of vault of skull with other and unspecified intracranial hemorrhage, with no loss of consciousness |
| 80032 | ICD9 | Closed fracture of vault of skull with other and unspecified intracranial hemorrhage, with brief [less than one hour] loss of consciousness |
| 80033 | ICD9 | Closed fracture of vault of skull with other and unspecified intracranial hemorrhage, with moderate [1-24 hours] loss of consciousness |
| 80034 | ICD9 | Closed fracture of vault of skull with other and unspecified intracranial hemorrhage, with prolonged [more than 24 hours] loss of consciousness and return to pre-existing conscious level |
| 80035 | ICD9 | Closed fracture of vault of skull with other and unspecified intracranial hemorrhage, with prolonged [more than 24 hours] loss of consciousness, without return to pre-existing conscious level |
| 80036 | ICD9 | Closed fracture of vault of skull with other and unspecified intracranial hemorrhage, with loss of consciousness of unspecified duration |
| 80129 | ICD9 | Closed fracture of base of skull with subarachnoid, subdural, and extradural hemorrhage, with concussion, unspecified |
| 80130 | ICD9 | Closed fracture of base of skull with other and unspecified intracranial hemorrhage, unspecified state of consciousness |
| 80131 | ICD9 | Closed fracture of base of skull with other and unspecified intracranial hemorrhage, with no loss of consciousness |
| 80132 | ICD9 | Closed fracture of base of skull with other and unspecified intracranial hemorrhage, with brief [less than one hour] loss of consciousness |
| 80133 | ICD9 | Closed fracture of base of skull with other and unspecified intracranial hemorrhage, with moderate [1-24 hours] loss of consciousness |
| 80134 | ICD9 | Closed fracture of base of skull with other and unspecified intracranial hemorrhage, with prolonged [more than 24 hours] loss of consciousness and return to pre-existing conscious level |
| 80135 | ICD9 | Closed fracture of base of skull with other and unspecified intracranial hemorrhage, with prolonged [more than 24 hours] loss of consciousness, without return to pre-existing conscious level |
| 80136 | ICD9 | Closed fracture of base of skull with other and unspecified intracranial hemorrhage, with loss of consciousness of unspecified duration |
| 80139 | ICD9 | Closed fracture of base of skull with other and unspecified intracranial hemorrhage, with concussion, unspecified |
| 80140 | ICD9 | Closed fracture of base of skull with intracranial injury of other and unspecified nature, unspecified state of consciousness |
| 80141 | ICD9 | Closed fracture of base of skull with intracranial injury of other and unspecified nature, with no loss of consciousness |
| 80142 | ICD9 | Closed fracture of base of skull with intracranial injury of other and unspecified nature, with brief [less than one hour] loss of consciousness |
| 80143 | ICD9 | Closed fracture of base of skull with intracranial injury of other and unspecified nature, with moderate [1-24 hours] loss of consciousness |
| 80144 | ICD9 | Closed fracture of base of skull with intracranial injury of other and unspecified nature, with prolonged [more than 24 hours) loss of consciousness and return to pre-existing conscious level |
| 80145 | ICD9 | Closed fracture of base of skull with intracranial injury of other and unspecified nature, with prolonged [more than 24 hours] loss of consciousness, without return to pre-existing conscious level |
| 80146 | ICD9 | Closed fracture of base of skull with intracranial injury of other and unspecified nature, with loss of consciousness of unspecified duration |
| 80149 | ICD9 | Closed fracture of base of skull with intracranial injury of other and unspecified nature, with concussion, unspecified |
| 80150 | ICD9 | Open fracture of base of skull without mention of intracranial injury, unspecified state of consciousness |
| 80151 | ICD9 | Open fracture of base of skull without mention of intracranial injury, with no loss of consciousness |
| 80152 | ICD9 | Open fracture of base of skull without mention of intracranial injury, with brief [less than one hour] loss of consciousness |
| 80153 | ICD9 | Open fracture of base of skull without mention of intracranial injury, with moderate [1-24 hours] loss of consciousness |
| 80154 | ICD9 | Open fracture of base of skull without mention of intracranial injury, with prolonged [more than 24 hours] loss of consciousness and return to pre-existing conscious level |
| 80155 | ICD9 | Open fracture of base of skull without mention of intracranial injury, with prolonged [more than 24 hours] loss of consciousness, without return to pre-existing conscious level |
| 80156 | ICD9 | Open fracture of base of skull without mention of intracranial injury, with loss of consciousness of unspecified duration |
| 80159 | ICD9 | Open fracture of base of skull without mention of intracranial injury, with concussion, unspecified |
| 80160 | ICD9 | Open fracture of base of skull with cerebral laceration and contusion, unspecified state of consciousness |
| 80161 | ICD9 | Open fracture of base of skull with cerebral laceration and contusion, with no loss of consciousness |
| 80162 | ICD9 | Open fracture of base of skull with cerebral laceration and contusion, with brief [less than one hour] loss of consciousness |
| 80163 | ICD9 | Open fracture of base of skull with cerebral laceration and contusion, with moderate [1-24 hours] loss of consciousness |
| 80164 | ICD9 | Open fracture of base of skull with cerebral laceration and contusion, with prolonged [more than 24 hours] loss of consciousness and return to pre-existing conscious level |
| 80165 | ICD9 | Open fracture of base of skull with cerebral laceration and contusion, with prolonged [more than 24 hours] loss of consciousness, without return to pre-existing conscious level |
| 80166 | ICD9 | Open fracture of base of skull with cerebral laceration and contusion, with loss of consciousness of unspecified duration |
| 80169 | ICD9 | Open fracture of base of skull with cerebral laceration and contusion, with concussion, unspecified |
| 80170 | ICD9 | Open fracture of base of skull with subarachnoid, subdural, and extradural hemorrhage, unspecified state of consciousness |
| 80171 | ICD9 | Open fracture of base of skull with subarachnoid, subdural, and extradural hemorrhage, with no loss of consciousness |
| 80172 | ICD9 | Open fracture of base of skull with subarachnoid, subdural, and extradural hemorrhage, with brief [less than one hour] loss of consciousness |
| 80173 | ICD9 | Open fracture of base of skull with subarachnoid, subdural, and extradural hemorrhage, with moderate [1-24 hours] loss of consciousness |
| 85154 | ICD9 | Cerebellar or brain stem contusion with open intracranial wound, with prolonged [more than 24 hours] loss of consciousness and return |
| 85155 | ICD9 | Cerebellar or brain stem contusion with open intracranial wound, with prolonged [more than 24 hours] loss of consciousness without return to pre-existing conscious level |
| 85156 | ICD9 | Cerebellar or brain stem contusion with open intracranial wound, with loss of consciousness of unspecified duration |
| 85159 | ICD9 | Cerebellar or brain stem contusion with open intracranial wound, with concussion, unspecified |
| 85160 | ICD9 | Cerebellar or brain stem laceration without mention of open intracranial wound, unspecified state of consciousness |
| 85161 | ICD9 | Cerebellar or brain stem laceration without mention of open intracranial wound, with no loss of consciousness |
| 85162 | ICD9 | Cerebellar or brain stem laceration without mention of open intracranial wound, with brief [less than 1 hour] loss of consciousness |
| 85163 | ICD9 | Cerebellar or brain stem laceration without mention of open intracranial wound, with moderate [1-24 hours] loss of consciousness |
| 85164 | ICD9 | Cerebellar or brain stem laceration without mention of open intracranial wound, with prolonged [more than 24 hours] loss of consciousness and return to pre-existing conscious level |
| 85165 | ICD9 | Cerebellar or brain stem laceration without mention of open intracranial wound, with prolonged [more than 24 hours] loss of consciousness without return to pre-existing conscious level |
| 85166 | ICD9 | Cerebellar or brain stem laceration without mention of open intracranial wound, with loss of consciousness of unspecified duration |
| 85169 | ICD9 | Cerebellar or brain stem laceration without mention of open intracranial wound, with concussion, unspecified |
| 85170 | ICD9 | Cerebellar or brain stem laceration with open intracranial wound, unspecified state of consciousness |
| 85171 | ICD9 | Cerebellar or brain stem laceration with open intracranial wound, with no loss of consciousness |
| 85172 | ICD9 | Cerebellar or brain stem laceration with open intracranial wound, with brief [less than one hour] loss of consciousness |
| 85173 | ICD9 | Cerebellar or brain stem laceration with open intracranial wound, with moderate [1-24 hours] loss of consciousness |
| 85174 | ICD9 | Cerebellar or brain stem laceration with open intracranial wound, with prolonged [more than 24 hours] loss of consciousness and return to pre-existing conscious level |
| 85175 | ICD9 | Cerebellar or brain stem laceration with open intracranial wound, with prolonged [more than 24 hours] loss of consciousness without return to pre-existing conscious level |
| 85176 | ICD9 | Cerebellar or brain stem laceration with open intracranial wound, with loss of consciousness of unspecified duration |
| 85179 | ICD9 | Cerebellar or brain stem laceration with open intracranial wound, with concussion, unspecified |
| 85180 | ICD9 | Other and unspecified cerebral laceration and contusion, without mention of open intracranial wound, unspecified state of consciousness |
| 85181 | ICD9 | Other and unspecified cerebral laceration and contusion, without mention of open intracranial wound, with no loss of consciousness |
| 85182 | ICD9 | Other and unspecified cerebral laceration and contusion, without mention of open intracranial wound, with brief [less than one hour] loss of consciousness |
| 85183 | ICD9 | Other and unspecified cere85174bral laceration and contusion, without mention of open intracranial wound, with moderate [1-24 hours] loss of consciousness |
| 85184 | ICD9 | Other and unspecified cerebral laceration and contusion, without mention of open intracranial wound, with prolonged [more than 24 hours] loss of consciousness and return to pre- existing conscious level |
| 85185 | ICD9 | Other and unspecified cerebral laceration and contusion, without mention of open intracranial wound, with prolonged [more than 24 hours] loss of consciousness without return to pre-existing conscious level |
| 85186 | ICD9 | Other and unspecified cerebral laceration and contusion, without mention of open intracranial wound, with loss of consciousness of unspecified duration |
| 85189 | ICD9 | Other and unspecified cerebral laceration and contusion, without mention of open intracranial wound, with concussion, unspecified |
| 85190 | ICD9 | Other and unspecified cerebral laceration and contusion, with open intracranial wound, unspecified state of consciousness |
| 85191 | ICD9 | Other and unspecified cerebral laceration and contusion, with open intracranial wound, with no loss of consciousness |
| 85192 | ICD9 | Other and unspecified cerebral laceration and contusion, with open intracranial wound, with brief [less than one hour] loss of consciousness |
| 80039 | ICD9 | Closed fracture of vault of skull with other and unspecified intracranial hemorrhage, with concussion, unspecified |
| 80040 | ICD9 | Closed fracture of vault of skull with intracranial injury of other and unspecified nature, unspecified state of consciousness |
| 80041 | ICD9 | Closed fracture of vault of skull with intracranial injury of other and unspecified nature, with no loss of consciousness |
| 80042 | ICD9 | Closed fracture of vault of skull with intracranial injury of other and unspecified nature, with brief [less than one hour] loss of consciousness |
| 80043 | ICD9 | Closed fracture of vault of skull with intracranial injury of other and unspecified nature, with moderate [1-24 hours] loss of consciousness |
| 80044 | ICD9 | Closed fracture of vault of skull with intracranial injury of other and unspecified nature, with prolonged [more than 24 hours] loss of consciousness and return to pre-existing conscious level |
| 80045 | ICD9 | Closed fracture of vault of skull with intracranial injury of other and unspecified nature, with prolonged [more than 24 hours] loss of consciousness, without return to pre-existing conscious level |
| 80046 | ICD9 | Closed fracture of vault of skull with intracranial injury of other and unspecified nature, with loss of consciousness of unspecified duration |
| 80049 | ICD9 | Closed fracture of vault of skull with intracranial injury of other and unspecified nature, with concussion, unspecified |
| 80050 | ICD9 | Open fracture of vault of skull without mention of intracranial injury, unspecified state of consciousness |
| 80051 | ICD9 | Open fracture of vault of skull without mention of intracranial injury, with no loss of consciousness |
| 80052 | ICD9 | Open fracture of vault of skull without mention of intracranial injury, with brief [less than one hour] loss of consciousness |
| 80053 | ICD9 | Open fracture of vault of skull without mention of intracranial injury, with moderate [1-24 hours] loss of consciousness |
| 80054 | ICD9 | Open fracture of vault of skull without mention of intracranial injury, with prolonged [more than 24 hours] loss of consciousness and return to pre-existing conscious level |
| 80055 | ICD9 | Open fracture of vault of skull without mention of intracranial injury, with prolonged [more than 24 hours] loss of consciousness, without return to pre-existing conscious level |
| 80056 | ICD9 | Open fracture of vault of skull without mention of intracranial injury, with loss of consciousness of unspecified duration |
| 80059 | ICD9 | Open fracture of vault of skull without mention of intracranial injury, with concussion, unspecified |
| 80060 | ICD9 | Open fracture of vault of skull with cerebral laceration and contusion, unspecified state of consciousness |
| 80061 | ICD9 | Open fracture of vault of skull with cerebral laceration and contusion, with no loss of consciousness |
| 80062 | ICD9 | Open fracture of vault of skull with cerebral laceration and contusion, with brief [less than one hour] loss of consciousness |
| 80063 | ICD9 | Open fracture of vault of skull with cerebral laceration and contusion, with moderate [1-24 hours] loss of consciousness |
| 80064 | ICD9 | Open fracture of vault of skull with cerebral laceration and contusion, with prolonged [more than 24 hours] loss of consciousness and return to pre-existing conscious level |
| 80065 | ICD9 | Open fracture of vault of skull with cerebral laceration and contusion, with prolonged [more than 24 hours] loss of consciousness, without return to pre-existing conscious level |
| 80066 | ICD9 | Open fracture of vault of skull with cerebral laceration and contusion, with loss of consciousness of unspecified duration |
| 80069 | ICD9 | Open fracture of vault of skull with cerebral laceration and contusion, with concussion, unspecified |
| 80070 | ICD9 | Open fracture of vault of skull with subarachnoid, subdural, and extradural hemorrhage, unspecified state of consciousness |
| 80071 | ICD9 | Open fracture of vault of skull with subarachnoid, subdural, and extradural hemorrhage, with no loss of consciousness |
| 80072 | ICD9 | Open fracture of vault of skull with subarachnoid, subdural, and extradural hemorrhage, with brief [less than one hour] loss of consciousness |
| 80073 | ICD9 | Open fracture of vault of skull with subarachnoid, subdural, and extradural hemorrhage, with moderate [1-24 hours] loss of consciousness |
| 80074 | ICD9 | Open fracture of vault of skull with subarachnoid, subdural, and extradural hemorrhage, with moderate [1-24 hours] loss of consciousness |
| 80075 | ICD9 | Open fracture of vault of skull with subarachnoid, subdural, and extradural hemorrhage, with prolonged [more than 24 hours] loss of consciousness, without return to pre-existing conscious level |
| 80076 | ICD9 | Open fracture of vault of skull with subarachnoid, subdural, and extradural hemorrhage, with loss of consciousness of unspecified duration |
| 80079 | ICD9 | Open fracture of vault of skull with subarachnoid, subdural, and extradural hemorrhage, with concussion, unspecified |
| 80080 | ICD9 | Open fracture of vault of skull with other and unspecified intracranial hemorrhage, unspecified state of consciousness |
| 80081 | ICD9 | Open fracture of vault of skull with other and unspecified intracranial hemorrhage, with no loss of consciousness |
| 80082 | ICD9 | Open fracture of vault of skull with other and unspecified intracranial hemorrhage, with brief [less than one hour] loss of |
| 80083 | ICD9 | Open fracture of vault of skull with other and unspecified intracranial hemorrhage, with moderate [1-24 hours] loss of consciousness |
| 80084 | ICD9 | Open fracture of vault of skull with other and unspecified intracranial hemorrhage, with prolonged [more than 24 hours] loss of consciousness and return to pre-existing conscious level |
| 80085 | ICD9 | Open fracture of vault of skull with other and unspecified intracranial hemorrhage, with prolonged [more than 24 hours] loss of consciousness, without return to pre-existing conscious level |
| 80086 | ICD9 | Open fracture of vault of skull with other and unspecified intracranial hemorrhage, with loss of consciousness of unspecified duration |
| 80089 | ICD9 | Open fracture of vault of skull with other and unspecified intracranial hemorrhage, with concussion, unspecified |
| 80090 | ICD9 | Open fracture of vault of skull with intracranial injury of other and unspecified nature, unspecified state of consciousness |
| 80091 | ICD9 | Open fracture of vault of skull with intracranial injury of other and unspecified nature, with no loss of consciousness |
| 80092 | ICD9 | Open fracture of vault of skull with intracranial injury of other and unspecified nature, with brief [less than one hour] loss of consciousness |
| 80093 | ICD9 | Open fracture of vault of skull with intracranial injury of other and unspecified nature, with moderate [1-24 hours] loss of consciousness |
| 80094 | ICD9 | Open fracture of vault of skull with intracranial injury of other and unspecified nature, with prolonged [more than 24 hours] loss of consciousness and return to pre-existing conscious level |
| 80095 | ICD9 | Open fracture of vault of skull with intracranial injury of other and unspecified nature, with prolonged [more than 24 hours] loss of consciousness, without return to pre-existing conscious level |
| 80096 | ICD9 | Open fracture of vault of skull with intracranial injury of other and unspecified nature, with loss of consciousness of unspecified duration |
| 80099 | ICD9 | Open fracture of vault of skull with intracranial injury of other and unspecified nature, with concussion, unspecified |
| 80100 | ICD9 | Closed fracture of base of skull without mention of intra cranial injury, unspecified state of consciousness |
| 80101 | ICD9 | Closed fracture of base of skull without mention of intra cranial injury, with no loss of consciousness |
| 80102 | ICD9 | Closed fracture of base of skull without mention of intra cranial injury, with brief [less than one hour] loss of consciousness |
| 80103 | ICD9 | Closed fracture of base of skull without mention of intra cranial injury, with moderate [1-24 hours] loss of consciousness |
| 80104 | ICD9 | Closed fracture of base of skull without mention of intra cranial injury, with prolonged [more than 24 hours] loss of consciousness and return to pre-existing conscious level |
| 80105 | ICD9 | Closed fracture of base of skull without mention of intra cranial injury, with prolonged [more than 24 hours] loss of consciousness, without return to pre-existing conscious level |
| 80106 | ICD9 | Closed fracture of base of skull without mention of intra cranial injury, with loss of consciousness of unspecified duration |
| 80109 | ICD9 | Closed fracture of base of skull without mention of intra cranial injury, with concussion, unspecified |
| 80110 | ICD9 | Closed fracture of base of skull with cerebral laceration and contusion, unspecified state of consciousness |
| 80111 | ICD9 | Closed fracture of base of skull with cerebral laceration and contusion, with no loss of consciousness |
| 80112 | ICD9 | Closed fracture of base of skull with cerebral laceration and contusion, with brief [less than one hour] loss of consciousness |
| 80113 | ICD9 | Closed fracture of base of skull with cerebral laceration and contusion, with moderate [1-24 hours] loss of consciousness |
| 80114 | ICD9 | Closed fracture of base of skull with cerebral laceration and contusion, with prolonged [more than 24 hours] loss of consciousness and return to pre-existing conscious level |
| 80115 | ICD9 | Closed fracture of base of skull with cerebral laceration and contusion, with prolonged [more than 24 hours] loss of consciousness, without return to pre-existing conscious level |
| 80116 | ICD9 | Closed fracture of base of skull with cerebral laceration and contusion, with loss of consciousness of unspecified duration |
| 80119 | ICD9 | Closed fracture of base of skull with cerebral laceration and contusion, with concussion, unspecified |
| 80120 | ICD9 | Closed fracture of base of skull with subarachnoid, subdural, and extradural hemorrhage, unspecified state of consciousness |
| 80121 | ICD9 | Closed fracture of base of skull with subarachnoid, subdural, and extradural hemorrhage, with no loss of consciousness |
| 80122 | ICD9 | Closed fracture of base of skull with subarachnoid, subdural, and extradural hemorrhage, with brief [less than one hour] loss of consciousness |
| 80123 | ICD9 | Closed fracture of base of skull with subarachnoid, subdural, and extradural hemorrhage, with moderate [1-24 hours] loss of consciousness |
| 80124 | ICD9 | Closed fracture of base of skull with subarachnoid, subdural, and extradural hemorrhage, with prolonged [more than 24 hours] loss of consciousness and return to pre-existing conscious level |
| 80300 | ICD9 | Other closed skull fracture without mention of intracranial injury, unspecified state of consciousness |
| 80301 | ICD9 | Other closed skull fracture without mention of intracranial injury, with no loss of consciousness |
| 80302 | ICD9 | Other closed skull fracture without mention of intracranial injury, with brief [less than one hour] loss of consciousness |
| 80303 | ICD9 | Other closed skull fracture without mention of intracranial injury, with moderate [1-24 hours] loss of consciousness |
| 80304 | ICD9 | Other closed skull fracture without mention of intracranial injury, with prolonged [more than 24 hours] loss of consciousness and return to pre-existing conscious level |
| 80305 | ICD9 | Other closed skull fracture without mention of intracranial injury, with prolonged [more than 24 hours] loss of consciousness, without return to pre-existing conscious level |
| 80306 | ICD9 | Other closed skull fracture without mention of intracranial injury, with loss of consciousness of unspecified duration |
| 80309 | ICD9 | Other closed skull fracture without mention of intracranial injury, with concussion, unspecified |
| 80310 | ICD9 | Other closed skull fracture with cerebral laceration and contusion, unspecified state of consciousness |
| 80311 | ICD9 | Other closed skull fracture with cerebral laceration and contusion, with no loss of consciousness |
| 80312 | ICD9 | Other closed skull fracture with cerebral laceration and contusion, with brief [less than one hour] loss of consciousness |
| 80313 | ICD9 | Other closed skull fracture with cerebral laceration and contusion, with moderate [1-24 hours] loss of consciousness |
| 80314 | ICD9 | Other closed skull fracture with cerebral laceration and contusion, with prolonged [more than 24 hours] loss of consciousness and return to pre-existing conscious level |
| 80315 | ICD9 | Other closed skull fracture with cerebral laceration and contusion, with prolonged [more than 24 hours] loss of consciousness, without return to pre-existing conscious level |
| 80316 | ICD9 | Other closed skull fracture with cerebral laceration and contusion, with loss of consciousness of unspecified duration |
| 80319 | ICD9 | Other closed skull fracture with cerebral laceration and contusion, with concussion, unspecified |
| 80320 | ICD9 | Other closed skull fracture with subarachnoid, subdural, and extradural hemorrhage, unspecified state of consciousness |
| 80321 | ICD9 | Other closed skull fracture with subarachnoid, subdural, and extradural hemorrhage, with no loss of consciousness |
| 80322 | ICD9 | Other closed skull fracture with subarachnoid, subdural, and extradural hemorrhage, with brief [less than one hour] loss of consciousness |
| 80323 | ICD9 | Other closed skull fracture with subarachnoid, subdural, and extradural hemorrhage, with moderate [1-24 hours] loss of consciousness |
| 80324 | ICD9 | Other closed skull fracture with subarachnoid, subdural, and extradural hemorrhage, with prolonged [more than 24 hours] loss of consciousness and return to pre-existing conscious level |
| 80325 | ICD9 | Other closed skull fracture with subarachnoid, subdural, and extradural hemorrhage, with prolonged [more than 24 hours] loss of consciousness, without return to pre-existing conscious level |
| 80326 | ICD9 | Other closed skull fracture with subarachnoid, subdural, and extradural hemorrhage, with loss of consciousness of unspecified duration |
| 80329 | ICD9 | Other closed skull fracture with subarachnoid, subdural, and extradural hemorrhage, with concussion, unspecified |
| 80330 | ICD9 | Other closed skull fracture with other and unspecified intracranial hemorrhage, unspecified state of unconsciousness |
| 80331 | ICD9 | Other closed skull fracture with other and unspecified intracranial hemorrhage, with no loss of consciousness |
| 80332 | ICD9 | Other closed skull fracture with other and unspecified intracranial hemorrhage, with brief [less than one hour] loss of consciousness |
| 80333 | ICD9 | Other closed skull fracture with other and unspecified intracranial hemorrhage, with moderate [1-24 hours] loss of consciousness |
| 80334 | ICD9 | Other closed skull fracture with other and unspecified intracranial hemorrhage, with prolonged [more than 24 hours] loss of consciousness and return to pre-existing conscious level |
| 80335 | ICD9 | Other closed skull fracture with other and unspecified intracranial hemorrhage, with prolonged [more than 24 hours] loss of consciousness, without return to pre-existing conscious level |
| 80336 | ICD9 | Other closed skull fracture with other and unspecified intracranial hemorrhage, with loss of consciousness of unspecified duration |
| 80339 | ICD9 | Other closed skull fracture with other and unspecified intracranial hemorrhage, with concussion, unspecified |
| 80340 | ICD9 | Other closed skull fracture with intracranial injury of other and unspecified nature, unspecified state of consciousness |
| 80341 | ICD9 | Other closed skull fracture with intracranial injury of other and unspecified nature, with no loss of consciousness |
| 80342 | ICD9 | Other closed skull fracture with intracranial injury of other and unspecified nature, with brief [less than one hour] loss of consciousness |
| 80343 | ICD9 | Other closed skull fracture with intracranial injury of other and unspecified nature, with moderate [1-24 hours] loss of consciousness |
| 80344 | ICD9 | Other closed skull fracture with intracranial injury of other and unspecified nature, with prolonged [more than 24 hours] loss of consciousness and return to pre-existing conscious level |
| 80345 | ICD9 | Other closed skull fracture with intracranial injury of other and unspecified nature, with prolonged [more than 24 hours] loss of consciousness, without return to pre-existing conscious level |
| 8503 | ICD9 | Concussion with prolonged loss of consciousness and return to pre-existing conscious level |
| 85193 | ICD9 | Other and unspecified cerebral laceration and contusion, with open intracranial wound, with moderate [1-24 hours] loss of consciousness |
| 85194 | ICD9 | Other and unspecified cerebral laceration and contusion, with open intracranial wound, with prolonged [more than 24 hours] loss of consciousness and return to pre-existing conscious level |
| 85195 | ICD9 | Other and unspecified cerebral laceration and contusion, with open intracranial wound, with prolonged [more than 24 hours] loss of consciousness without return to pre-existing conscious level |
| 85196 | ICD9 | Other and unspecified cerebral laceration and contusion, with open intracranial wound, with loss of consciousness of unspecified duration |
| 85199 | ICD9 | Other and unspecified cerebral laceration and contusion, with open intracranial wound, with concussion, unspecified |
| 85200 | ICD9 | Subarachnoid hemorrhage following injury without mention of open intracranial wound, unspecified state of consciousness |
| 85201 | ICD9 | Subarachnoid hemorrhage following injury without mention of open intracranial wound, with no loss of consciousness |
| 85412 | ICD9 | Intracranial injury of other and unspecified nature with open intracranial wound, with brief [less than one hour] loss of consciousness |
| 85413 | ICD9 | Intracranial injury of other and unspecified nature with open intracranial wound, with moderate [1-24 hours] loss of consciousness |
| 85414 | ICD9 | Intracranial injury of other and unspecified nature with open intracranial wound, with prolonged [more than 24 hours] loss of consciousness and return to pre-existing conscious level |
| 85415 | ICD9 | Intracranial injury of other and unspecified nature with open intracranial wound, with prolonged [more than 24 hours] loss of consciousness without return to pre-existing conscious level |
| 85416 | ICD9 | Intracranial injury of other and unspecified nature with open intracranial wound, with loss of consciousness of unspecified duration |
| 85419 | ICD9 | Intracranial injury of other and unspecified nature with open intracranial wound, with concussion, unspecified |
| 80411 | ICD9 | Closed fractures involving skull or face with other bones, with cerebral laceration and contusion, with no loss of consciousness |
| 80420 | ICD9 | Closed fractures involving skull or face with other bones with subarachnoid, subdural, and extradural hemorrhage, unspecified state of consciousness |
| 80421 | ICD9 | Closed fractures involving skull or face with other bones with subarachnoid, subdural, and extradural hemorrhage, with no loss of consciousness |
| 80422 | ICD9 | Closed fractures involving skull or face with other bones with subarachnoid, subdural, and extradural hemorrhage, with brief [less than one hour] loss of consciousness |
| 80423 | ICD9 | Closed fractures involving skull or face with other bones with subarachnoid, subdural, and extradural hemorrhage, with moderate [1-24 hours] loss of consciousness |
| 80424 | ICD9 | Closed fractures involving skull or face with other bones with subarachnoid, subdural, and extradural hemorrhage, with prolonged [more than 24 hours] loss of consciousness and return to pre-existing conscious level |
| 80425 | ICD9 | Closed fractures involving skull or face with other bones with subarachnoid, subdural, and extradural hemorrhage, with prolonged [more than 24 hours] loss of consciousness, without return to pre-existing conscious level |
| 80426 | ICD9 | Closed fractures involving skull or face with other bones with subarachnoid, subdural, and extradural hemorrhage, with loss of consciousness of unspecified duration |
| 80429 | ICD9 | Closed fractures involving skull or face with other bones with subarachnoid, subdural, and extradural hemorrhage, with concussion, unspecified |
| 80430 | ICD9 | Closed fractures involving skull or face with other bones, with other and unspecified intracranial hemorrhage, unspecified state of consciousness |
| 80431 | ICD9 | Closed fractures involving skull or face with other bones, with other and unspecified intracranial hemorrhage, with no loss of consciousness |
| 80432 | ICD9 | Closed fractures involving skull or face with other bones, with other and unspecified intracranial hemorrhage, with brief [less than one hour] loss of consciousness |
| 80433 | ICD9 | Closed fractures involving skull or face with other bones, with other and unspecified intracranial hemorrhage, with moderate [1-24 hours] loss of consciousness |
| 85250 | ICD9 | Extradural hemorrhage following injury with open intracranial wound, unspecified state of consciousness |
| 85251 | ICD9 | Extradural hemorrhage following injury with open intracranial wound, with no loss of consciousness |
| 85252 | ICD9 | Extradural hemorrhage following injury with open intracranial wound, with brief [less than one hour] loss of consciousness |
| 85253 | ICD9 | Extradural hemorrhage following injury with open intracranial wound, with moderate [1-24 hours] loss of consciousness |
| 85254 | ICD9 | Extradural hemorrhage following injury with open intracranial wound, with prolonged [more than 24 hours] loss of consciousness and return to pre-existing conscious level |
| 85255 | ICD9 | Extradural hemorrhage following injury with open intracranial wound, with prolonged [more than 24 hours] loss of consciousness without return to pre-existing conscious level |
| 85256 | ICD9 | Extradural hemorrhage following injury with open intracranial wound, with loss of consciousness of unspecified duration |
| 85259 | ICD9 | Extradural hemorrhage following injury with open intracranial wound, with concussion, unspecified |
| 85300 | ICD9 | Other and unspecified intracranial hemorrhage following injury without mention of open intracranial wound, unspecified state of consciousness |
| 85301 | ICD9 | Other and unspecified intracranial hemorrhage following injury without mention of open intracranial wound, with no loss of consciousness |
| 85302 | ICD9 | Other and unspecified intracranial hemorrhage following injury without mention of open intracranial wound, with brief [less than one hour] loss of consciousness |
| 85303 | ICD9 | Other and unspecified intracranial hemorrhage following injury without mention of open intracranial wound, with moderate [1-24 hours] loss of consciousness |
| 85304 | ICD9 | Other and unspecified intracranial hemorrhage following injury without mention of open intracranial wound, with prolonged [more than 24 hours] loss of consciousness and return to pre- existing conscious level |
| 85305 | ICD9 | Other and unspecified intracranial hemorrhage following injury without mention of open intracranial wound, with prolonged [more than 24 hours] loss of consciousness without return to pre-existing conscious level |
| 85306 | ICD9 | Other and unspecified intracranial hemorrhage following injury without mention of open intracranial wound, with loss of consciousness of unspecified duration |
| 85309 | ICD9 | Other and unspecified intracranial hemorrhage following injury without mention of open intracranial wound, with concussion, unspecified |
| 85310 | ICD9 | Other and unspecified intracranial hemorrhage following injury with open intracranial wound, unspecified state of consciousness |
| 85311 | ICD9 | Other and unspecified intracranial hemorrhage following injury with open intracranial wound, with no loss of consciousness |
| 85312 | ICD9 | Other and unspecified intracranial hemorrhage following injury with open intracranial wound, with brief [less than one hour] loss of consciousness |
| 85313 | ICD9 | Other and unspecified intracranial hemorrhage following injury with open intracranial wound, with moderate [1-24 hours] loss of consciousness |
| 85314 | ICD9 | Other and unspecified intracranial hemorrhage following injury with open intracranial wound, with prolonged [more than 24 hours] loss of consciousness and return to pre-existing conscious level |
| 85315 | ICD9 | Other and unspecified intracranial hemorrhage following injury with open intracranial wound, with prolonged [more than 24 hours] loss of consciousness without return to pre-existing conscious level |
| 85316 | ICD9 | Other and unspecified intracranial hemorrhage following injury with open intracranial wound, with loss of consciousness of unspecified duration |
| 85319 | ICD9 | Other and unspecified intracranial hemorrhage following injury with open intracranial wound, with concussion, unspecified |
| 85400 | ICD9 | Intracranial injury of other and unspecified nature without mention of open intracranial wound, unspecified state of consciousness |
| 85401 | ICD9 | Intracranial injury of other and unspecified nature without mention of open intracranial wound, with no loss of consciousness |
| 85402 | ICD9 | Intracranial injury of other and unspecified nature without mention of open intracranial wound, with brief [less than one hour] loss of consciousness |
| 85403 | ICD9 | Intracranial injury of other and unspecified nature without mention of open intracranial wound, with moderate [1-24 hours] loss of consciousness |
| 85404 | ICD9 | Intracranial injury of other and unspecified nature without mention of open intracranial wound, with prolonged [more than 24 hours] loss of consciousness and return to pre-existing conscious level |
| 85405 | ICD9 | Intracranial injury of other and unspecified nature without mention of open intracranial wound, with prolonged [more than 24 hours] loss of consciousness without return to pre-existing conscious level |
| 85406 | ICD9 | Intracranial injury of other and unspecified nature without mention of open intracranial wound, with loss of consciousness of unspecified duration |
| 85409 | ICD9 | Intracranial injury of other and unspecified nature without mention of open intracranial wound, with concussion, unspecified |
| 85410 | ICD9 | Intracranial injury of other and unspecified nature with open intracranial wound, unspecified state of consciousness |
| 85411 | ICD9 | Intracranial injury of other and unspecified nature with open intracranial wound, with no loss of consciousness |
| 85412 | ICD9 | Intracranial injury of other and unspecified nature with open intracranial wound, with brief [less than one hour] loss of consciousness |
| 85413 | ICD9 | Intracranial injury of other and unspecified nature with open intracranial wound, with moderate [1-24 hours] loss of consciousness |
| 85414 | ICD9 | Intracranial injury of other and unspecified nature with open intracranial wound, with prolonged [more than 24 hours] loss of consciousness and return to pre-existing conscious level |
| 85415 | ICD9 | Intracranial injury of other and unspecified nature with open intracranial wound, with prolonged [more than 24 hours] loss of consciousness without return to pre-existing conscious level |
| 85416 | ICD9 | Intracranial injury of other and unspecified nature with open intracranial wound, with loss of consciousness of unspecified duration |
| 85419 | ICD9 | Intracranial injury of other and unspecified nature with open intracranial wound, with concussion, unspecified |
| 80434 | ICD9 | Closed fractures involving skull or face with other bones, with other and unspecified intracranial hemorrhage, with prolonged [more than 24 hours] loss of consciousness and return to pre- existing conscious level |
| 80435 | ICD9 | Closed fractures involving skull or face with other bones, with other and unspecified intracranial hemorrhage, with prolonged [more than 24 hours] loss of consciousness, without return to pre-existing conscious level |
| 80436 | ICD9 | Closed fractures involving skull or face with other bones, with other and unspecified intracranial hemorrhage, with loss of consciousness of unspecified duration |
| 80439 | ICD9 | Closed fractures involving skull or face with other bones, with other and unspecified intracranial hemorrhage, with concussion, unspecified |
| 80440 | ICD9 | Closed fractures involving skull or face with other bones, with intracranial injury of other and unspecified nature, unspecified state of consciousness |
| 80441 | ICD9 | Closed fractures involving skull or face with other bones, with intracranial injury of other and unspecified nature, with no loss of consciousness |
| 80442 | ICD9 | Closed fractures involving skull or face with other bones, with intracranial injury of other and unspecified nature, with brief [less than one hour] loss of consciousness |
| 80443 | ICD9 | Closed fractures involving skull or face with other bones, with intracranial injury of other and unspecified nature, with moderate [1-24 hours] loss of consciousness |
| 80444 | ICD9 | Closed fractures involving skull or face with other bones, with intracranial injury of other and unspecified nature, with prolonged [more than 24 hours] loss of consciousness and return to pre-existing conscious level |
| 80445 | ICD9 | Closed fractures involving skull or face with other bones, with intracranial injury of other and unspecified nature, with prolonged [more than 24 hours] loss of consciousness, without return to pre-existing conscious level |
| 80446 | ICD9 | Closed fractures involving skull or face with other bones, with intracranial injury of other and unspecified nature, with loss of consciousness of unspecified duration |
| 80449 | ICD9 | Closed fractures involving skull or face with other bones, with intracranial injury of other and unspecified nature, with concussion, unspecified |
| 80450 | ICD9 | Open fractures involving skull or face with other bones, without mention of intracranial injury, unspecified state of consciousness |
| 80451 | ICD9 | Open fractures involving skull or face with other bones, without mention of intracranial injury, with no loss of consciousness |
| 80452 | ICD9 | Open fractures involving skull or face with other bones, without mention of intracranial injury, with brief [less than one hour] loss of consciousness |
| 80453 | ICD9 | Open fractures involving skull or face with other bones, without mention of intracranial injury, with moderate [1-24 hours] loss of consciousness |
| 80454 | ICD9 | Open fractures involving skull or face with other bones, without mention of intracranial injury, with prolonged [more than 24 hours] loss of consciousness and return to pre-existing conscious level |
| 80455 | ICD9 | Open fractures involving skull or face with other bones, without mention of intracranial injury, with prolonged [more than 24 hours] loss of consciousness, without return to pre-existing conscious level |
| 80456 | ICD9 | Open fractures involving skull or face with other bones, without mention of intracranial injury, with loss of consciousness of unspecified duration |
| 80459 | ICD9 | Open fractures involving skull or face with other bones, without mention of intracranial injury, with concussion, unspecified |
| 80460 | ICD9 | Open fractures involving skull or face with other bones, with cerebral laceration and contusion, unspecified state of consciousness |
| 80461 | ICD9 | Open fractures involving skull or face with other bones, with cerebral laceration and contusion, with no loss of consciousness |
| 80462 | ICD9 | Open fractures involving skull or face with other bones, with cerebral laceration and contusion, with brief [less than one hour] loss of consciousness |
| 80463 | ICD9 | Open fractures involving skull or face with other bones, with cerebral laceration and contusion, with moderate [1-24 hours] loss of consciousness |
| 80464 | ICD9 | Open fractures involving skull or face with other bones, with cerebral laceration and contusion, with prolonged [more than 24 hours] loss of consciousness and return to pre-existing conscious level |
| 80465 | ICD9 | Open fractures involving skull or face with other bones, with cerebral laceration and contusion, with prolonged [more than 24 hours] loss of consciousness, without return to pre-existing conscious level |
| 80466 | ICD9 | Open fractures involving skull or face with other bones, with cerebral laceration and contusion, with loss of consciousness of unspecified duration |
| 80469 | ICD9 | Open fractures involving skull or face with other bones, with cerebral laceration and contusion, with concussion, unspecified |
| 80470 | ICD9 | Open fractures involving skull or face with other bones with subarachnoid, subdural, and extradural hemorrhage, unspecified state of consciousness |
| 80471 | ICD9 | Open fractures involving skull or face with other bones with subarachnoid, subdural, and extradural hemorrhage, with no loss of consciousness |
| 80472 | ICD9 | Open fractures involving skull or face with other bones with subarachnoid, subdural, and extradural hemorrhage, with brief [less than one hour] loss of consciousness |
| 80473 | ICD9 | Open fractures involving skull or face with other bones with subarachnoid, subdural, and extradural hemorrhage, with moderate [1-24 hours] loss of consciousness |
| 85103 | ICD9 | Cortex (cerebral) contusion without mention of open intracranial wound, with moderate [1-24 hours] loss of consciousness |
| 85104 | ICD9 | Cortex (cerebral) contusion without mention of open intracranial wound, with prolonged [more than 24 hours] loss of consciousness and return to pre-existing conscious level |
| 85105 | ICD9 | Cortex (cerebral) contusion without mention of open intracranial wound, with prolonged [more than 24 hours] loss of consciousness without return to pre-existing conscious level |
| 85106 | ICD9 | Cortex (cerebral) contusion without mention of open intracranial wound, with loss of consciousness of unspecified duration |
| 80346 | ICD9 | Other closed skull fracture with intracranial injury of other and unspecified nature, with loss of consciousness of unspecified duration |
| 80349 | ICD9 | Other closed skull fracture with intracranial injury of other and unspecified nature, with concussion, unspecified |
| 80350 | ICD9 | Other open skull fracture without mention of injury, unspecified state of consciousness |
| 80351 | ICD9 | Other open skull fracture without mention of intracranial injury, with no loss of consciousness |
| 80352 | ICD9 | Other open skull fracture without mention of intracranial injury, with brief [less than one hour] loss of consciousness |
| 80353 | ICD9 | Other open skull fracture without mention of intracranial injury, with moderate [1-24 hours] loss of consciousness |
| 80354 | ICD9 | Other open skull fracture without mention of intracranial injury, with prolonged [more than 24 hours] loss of consciousness and return to pre-existing conscious level |
| 80355 | ICD9 | Other open skull fracture without mention of intracranial injury, with prolonged [more than 24 hours] loss of consciousness, without return to pre-existing conscious level |
| 80356 | ICD9 | Other open skull fracture without mention of intracranial injury, with loss of consciousness of unspecified duration |
| 80359 | ICD9 | Other open skull fracture without mention of intracranial injury, with concussion, unspecified |
| 80360 | ICD9 | Other open skull fracture with cerebral laceration and contusion, unspecified state of consciousness |
| 80361 | ICD9 | Other open skull fracture with cerebral laceration and contusion, with no loss of consciousness |
| 80362 | ICD9 | Other open skull fracture with cerebral laceration and contusion, with brief [less than one hour] loss of consciousness |
| 80363 | ICD9 | Other open skull fracture with cerebral laceration and contusion, with moderate [1-24 hours] loss of consciousness |
| 80364 | ICD9 | Other open skull fracture with cerebral laceration and contusion, with prolonged [more than 24 hours] loss of consciousness and return to pre-existing conscious level |
| 80365 | ICD9 | Other open skull fracture with cerebral laceration and contusion, with prolonged [more than 24 hours] loss of consciousness, without return to pre-existing conscious level |
| 80366 | ICD9 | Other open skull fracture with cerebral laceration and contusion, with loss of consciousness of unspecified duration |
| 80369 | ICD9 | Other open skull fracture with cerebral laceration and contusion, with concussion, unspecified |
| 80370 | ICD9 | Other open skull fracture with subarachnoid, subdural, and extradural hemorrhage, unspecified state of consciousness |
| 80371 | ICD9 | Other open skull fracture with subarachnoid, subdural, and extradural hemorrhage, with no loss of consciousness |
| 80372 | ICD9 | Other open skull fracture with subarachnoid, subdural, and extradural hemorrhage, with brief [less than one hour] loss of consciousness |
| 80373 | ICD9 | Other open skull fracture with subarachnoid, subdural, and extradural hemorrhage, with moderate [1-24 hours] loss of consciousness |
| 80374 | ICD9 | Other open skull fracture with subarachnoid, subdural, and extradural hemorrhage, with prolonged [more than 24 hours] loss of consciousness and return to pre-existing conscious level |
| 80375 | ICD9 | Other open skull fracture with subarachnoid, subdural, and extradural hemorrhage, with prolonged [more than 24 hours] loss of consciousness, without return to pre-existing conscious level |
| 80376 | ICD9 | Other open skull fracture with subarachnoid, subdural, and extradural hemorrhage, with loss of consciousness of unspecified duration |
| 80379 | ICD9 | Other open skull fracture with subarachnoid, subdural, and extradural hemorrhage, with concussion, unspecified |
| 80380 | ICD9 | Other open skull fracture with other and unspecified intracranial hemorrhage, unspecified state of consciousness |
| 80381 | ICD9 | Other open skull fracture with other and unspecified intracranial hemorrhage, with no loss of consciousness |
| 80382 | ICD9 | Other open skull fracture with other and unspecified intracranial hemorrhage, with brief [less than one hour] loss of consciousness |
| 80383 | ICD9 | Other open skull fracture with other and unspecified intracranial hemorrhage, with moderate [1-24 hours] loss of consciousness |
| 80384 | ICD9 | Other open skull fracture with other and unspecified intracranial hemorrhage, with prolonged [more than 24 hours] loss of consciousness and return to pre-existing conscious level |
| 80385 | ICD9 | Other open skull fracture with other and unspecified intracranial hemorrhage, with prolonged [more than 24 hours] loss of consciousness, without return to pre-existing conscious level |
| 80386 | ICD9 | Other open skull fracture with other and unspecified intracranial hemorrhage, with loss of consciousness of unspecified duration |
| 80389 | ICD9 | Other open skull fracture with other and unspecified intracranial hemorrhage, with concussion, unspecified |
| 80390 | ICD9 | Other open skull fracture with intracranial injury of other and unspecified nature, unspecified state of consciousness |
| 80391 | ICD9 | Other open skull fracture with intracranial injury of other and unspecified nature, with no loss of consciousness |
| 80392 | ICD9 | Other open skull fracture with intracranial injury of other and unspecified nature, with brief [less than one hour] loss of consciousness |
| 80393 | ICD9 | Other open skull fracture with intracranial injury of other and unspecified nature, with moderate [1-24 hours] loss of consciousness |
| 80394 | ICD9 | Other open skull fracture with intracranial injury of other and unspecified nature, with prolonged [more than 24 hours] loss of consciousness and return to pre-existing conscious level |
| 80395 | ICD9 | Other open skull fracture with intracranial injury of other and unspecified nature, with prolonged [more than 24 hours] loss of consciousness, without return to pre-existing conscious level |
| 80396 | ICD9 | Other open skull fracture with intracranial injury of other and unspecified nature, with loss of consciousness of unspecified duration |
| 80399 | ICD9 | Other open skull fracture with intracranial injury of other and unspecified nature, with concussion, unspecified |
| 80400 | ICD9 | Closed fractures involving skull or face with other bones, without mention of intracranial injury, unspecified state of consciousness |
| 80401 | ICD9 | Closed fractures involving skull or face with other bones, without mention of intracranial injury, with no loss of consciousness |
| 80402 | ICD9 | Closed fractures involving skull or face with other bones, without mention of intracranial injury, with brief [less than one hour] loss of consciousness |
| 80403 | ICD9 | Closed fractures involving skull or face with other bones, without mention of intracranial injury, with moderate [1-24 hours] loss of consciousness |
| 80404 | ICD9 | Closed fractures involving skull or face with other bones, without mention or intracranial injury, with prolonged [more than 24 hours] loss of consciousness and return to pre-existing conscious level |
| 80405 | ICD9 | Closed fractures involving skull of face with other bones, without mention of intracranial injury, with prolonged [more than 24 hours] loss of consciousness, without return to pre-existing conscious level |
| 80406 | ICD9 | Closed fractures involving skull of face with other bones, without mention of intracranial injury, with loss of consciousness of unspecified duration |
| 80409 | ICD9 | Closed fractures involving skull of face with other bones, without mention of intracranial injury, with concussion, unspecified |
| 80410 | ICD9 | Closed fractures involving skull or face with other bones, with cerebral laceration and contusion, unspecified state of consciousness |
| 80474 | ICD9 | Open fractures involving skull or face with other bones with subarachnoid, subdural, and extradural hemorrhage, with prolonged [more than 24 hours] loss of consciousness and return to pre-existing conscious level |
| 80475 | ICD9 | Open fractures involving skull or face with other bones with subarachnoid, subdural, and extradural hemorrhage, with prolonged [more than 24 hours] loss of consciousness, without return to pre-existing conscious level |
| 80476 | ICD9 | Open fractures involving skull or face with other bones with subarachnoid, subdural, and extradural hemorrhage, with loss of consciousness of unspecified duration |
| 80479 | ICD9 | Open fractures involving skull or face with other bones with subarachnoid, subdural, and extradural hemorrhage, with concussion, unspecified |
| 80480 | ICD9 | Open fractures involving skull or face with other bones, with other and unspecified intracranial hemorrhage, unspecified state of consciousness |
| 80481 | ICD9 | Open fractures involving skull or face with other bones, with other and unspecified intracranial hemorrhage, with no loss of consciousness |
| 80482 | ICD9 | Open fractures involving skull or face with other bones, with other and unspecified intracranial hemorrhage, with brief [less than one hour] loss of consciousness |
| 80483 | ICD9 | Open fractures involving skull or face with other bones, with other and unspecified intracranial hemorrhage, with moderate [1-24 hours] loss of consciousness |
| 80484 | ICD9 | Open fractures involving skull or face with other bones, with other and unspecified intracranial hemorrhage, with prolonged [more than 24 hours] loss of consciousness and return to pre-existing conscious level |
| 80485 | ICD9 | Open fractures involving skull or face with other bones, with other and unspecified intracranial hemorrhage, with prolonged [more than 24 hours] loss consciousness, without return to pre-existing conscious level |
| 80486 | ICD9 | Open fractures involving skull or face with other bones, with other and unspecified intracranial hemorrhage, with loss of consciousness of unspecified duration |
| 80489 | ICD9 | Open fractures involving skull or face with other bones, with other and unspecified intracranial hemorrhage, with concussion, unspecified |
| 80490 | ICD9 | Open fractures involving skull or face with other bones, with intracranial injury of other and unspecified nature, unspecified state of consciousness |
| 80491 | ICD9 | Open fractures involving skull or face with other bones, with intracranial injury of other and unspecified nature, with no loss of consciousness |
| 80492 | ICD9 | Open fractures involving skull or face with other bones, with intracranial injury of other and unspecified nature, with brief [less than one hour] loss of consciousness |
| 80493 | ICD9 | Open fractures involving skull or face with other bones, with intracranial injury of other and unspecified nature, with moderate [1-24 hours] loss of consciousness |
| 80494 | ICD9 | Open fractures involving skull or face with other bones, with intracranial injury of other and unspecified nature, with prolonged [more than 24 hours] loss of consciousness and return to pre-existing conscious level |
| 80495 | ICD9 | Open fractures involving skull or face with other bones, with intracranial injury of other and unspecified nature, with prolonged [more than 24 hours] loss of consciousness without return to pre-existing conscious level |
| 80496 | ICD9 | Open fractures involving skull or face with other bones, with intracranial injury of other and unspecified nature, with loss of consciousness of unspecified duration |
| 80499 | ICD9 | Open fractures involving skull or face with other bones, with intracranial injury of other and unspecified nature, with concussion, unspecified |
| 85109 | ICD9 | Cortex (cerebral) contusion without mention of open intracranial wound, with concussion, unspecified |
| 85110 | ICD9 | Cortex (cerebral) contusion with open intracranial wound, unspecified state of consciousness |
| 85111 | ICD9 | Cortex (cerebral) contusion with open intracranial wound, with no loss of consciousness |
| 85112 | ICD9 | Cortex (cerebral) contusion with open intracranial wound, with brief [less than one hour] loss of consciousness |
| 85113 | ICD9 | Cortex (cerebral) contusion with open intracranial wound, with moderate [1-24 hours] loss of consciousness |
| 85114 | ICD9 | Cortex (cerebral) contusion with open intracranial wound, with prolonged [more than 24 hours] loss of consciousness and return to pre-existing conscious level |
| 85115 | ICD9 | Cortex (cerebral) contusion with open intracranial wound, with prolonged [more than 24 hours] loss of consciousness without return to pre-existing conscious level |
| 85116 | ICD9 | Cortex (cerebral) contusion with open intracranial wound, with loss of consciousness of unspecified duration |
| 85119 | ICD9 | Cortex (cerebral) contusion with open intracranial wound, with concussion, unspecified |
| 85120 | ICD9 | Cortex (cerebral) laceration without mention of open intracranial wound, unspecified state of consciousness |
| 85121 | ICD9 | Cortex (cerebral) laceration without mention of open intracranial wound, with no loss of consciousness |
| 85122 | ICD9 | Cortex (cerebral) laceration without mention of open intracranial wound, with brief [less than one hour] loss of consciousness |
| 85123 | ICD9 | Cortex (cerebral) laceration without mention of open intracranial wound, with moderate [1-24 hours] loss of consciousness |
| 85124 | ICD9 | Cortex (cerebral) laceration without mention of open intracranial wound, with prolonged [more than 24 hours] loss of consciousness and return to pre-existing conscious level |
| 85125 | ICD9 | Cortex (cerebral) laceration without mention of open intracranial wound, with prolonged [more than 24 hours] loss of consciousness |
| 85126 | ICD9 | Cortex (cerebral) laceration without mention of open intracranial wound, with loss of consciousness of unspecified duration |
| 85129 | ICD9 | Cortex (cerebral) laceration without mention of open intracranial wound, with concussion, unspecified |
| 85130 | ICD9 | Cortex (cerebral) laceration with open intracranial wound, unspecified state of consciousness |
| 85131 | ICD9 | Cortex (cerebral) laceration with open intracranial wound, with no loss of consciousness |
| 85132 | ICD9 | Cortex (cerebral) laceration with open intracranial wound, with brief [less than one hour] loss of consciousness |
| 85133 | ICD9 | Cortex (cerebral) laceration with open intracranial wound, with moderate [1-24 hours] loss of consciousness |
| 85134 | ICD9 | Cortex (cerebral) laceration with open intracranial wound, with prolonged [more than 24 hours] loss of consciousness and return to pre-existing conscious level |
| 85135 | ICD9 | Cortex (cerebral) laceration with open intracranial wound, with prolonged [more than 24 hours] loss of consciousness without return to pre-existing conscious level |
| 85136 | ICD9 | Cortex (cerebral) laceration with open intracranial wound, with loss of consciousness of unspecified duration |
| 85139 | ICD9 | Cortex (cerebral) laceration with open intracranial wound, with concussion, unspecified |
| 85140 | ICD9 | Cerebellar or brain stem contusion without mention of open intracranial wound, unspecified state of consciousness |
| 85141 | ICD9 | Cerebellar or brain stem contusion without mention of open intracranial wound, with no loss of consciousness |
| 85142 | ICD9 | Cerebellar or brain stem contusion without mention of open intracranial wound, with brief [less than one hour] loss of consciousness |
| 85143 | ICD9 | Cerebellar or brain stem contusion without mention of open intracranial wound, with moderate [1-24 hours] loss of consciousness |
| 85144 | ICD9 | Cerebellar or brain stem contusion without mention of open intracranial wound, with prolonged [more than 24 hours] loss consciousness and return to pre-existing conscious level |
| 85145 | ICD9 | Cerebellar or brain stem contusion without mention of open intracranial wound, with prolonged [more than 24 hours] loss of consciousness without return to pre-existing conscious level |
| 85146 | ICD9 | Cerebellar or brain stem contusion without mention of open intracranial wound, with loss of consciousness of unspecified duration |
| 85149 | ICD9 | Cerebellar or brain stem contusion without mention of open intracranial wound, with concussion, unspecified |
| 85150 | ICD9 | Cerebellar or brain stem contusion with open intracranial wound, unspecified state of consciousness |
| 85151 | ICD9 | Cerebellar or brain stem contusion with open intracranial wound, with no loss of consciousness |
| 85152 | ICD9 | Cerebellar or brain stem contusion with open intracranial wound, with brief [less than one hour] loss of consciousness |
| 85153 | ICD9 | Cerebellar or brain stem contusion with open intracranial wound, with moderate [1-24 hours] loss of consciousness |
| P108 | ICD10 | Other intracranial lacerations and hemorrhages due to birth injury |
| P109 | ICD10 | Unspecified intracranial laceration and hemorrhage due to birth injury |
| P258 | ICD10 | Other intracranial (nontraumatic) hemorrhages of newborn |
| S06810A | ICD10 | Injury of right internal carotid artery, intracranial portion, not elsewhere classified without loss of consciousness, initial encounter |
| S06810D | ICD10 | Injury of right internal carotid artery, intracranial portion, not elsewhere classified without loss of consciousness, subsequent encounter |
| S06810S | ICD10 | Injury of right internal carotid artery, intracranial portion, not elsewhere classified without loss of consciousness, sequela |
| S06811A | ICD10 | Injury of right internal carotid artery, intracranial portion, not elsewhere classified with loss of consciousness of 30 minutes or less, initial encounter |
| S06811D | ICD10 | Injury of right internal carotid artery, intracranial portion, not elsewhere classified with loss of consciousness of 30 minutes or less, subsequent encounter |
| S06811S | ICD10 | Injury of right internal carotid artery, intracranial portion, not elsewhere classified with loss of consciousness of 30 minutes or less, sequela |
| S06812A | ICD10 | Injury of right internal carotid artery, intracranial portion, not elsewhere classified with loss of consciousness of 31 minutes to 59 minutes, initial encounter |
| S06812D | ICD10 | Injury of right internal carotid artery, intracranial portion, not elsewhere classified with loss of consciousness of 31 minutes to 59 minutes, subsequent encounter |
| S06812D | ICD10 | Injury of right internal carotid artery, intracranial portion, not elsewhere classified with loss of consciousness of 31 minutes to 59 minutes, subsequent encounter |
| S06812S | ICD10 | Injury of right internal carotid artery, intracranial portion, not elsewhere classified with loss of consciousness of 31 minutes to 59 minutes, sequela |
| S06813A | ICD10 | Injury of right internal carotid artery, intracranial portion, not elsewhere classified with loss of consciousness of 1 hour to 5 hours 59 minutes, initial encounter |
| S06813D | ICD10 | Injury of right internal carotid artery, intracranial portion, not elsewhere classified with loss of consciousness of 1 hour to 5 hours 59 minutes, subsequent encounter |
| S06813S | ICD10 | Injury of right internal carotid artery, intracranial portion, not elsewhere classified with loss of consciousness of 1 hour to 5 hours 59 minutes, sequela |
| S06814A | ICD10 | Injury of right internal carotid artery, intracranial portion, not elsewhere classified with loss of consciousness of 6 hours to 24 hours, initial encounter |
| S06814D | ICD10 | Injury of right internal carotid artery, intracranial portion, not elsewhere classified with loss of consciousness of 6 hours to 24 hours, subsequent encounter |
| S06814S | ICD10 | Injury of right internal carotid artery, intracranial portion, not elsewhere classified with loss of consciousness of 6 hours to 24 hours, sequela |
| S06815A | ICD10 | Injury of right internal carotid artery, intracranial portion, not elsewhere classified with loss of consciousness greater than 24 hours with return to pre-existing conscious level, initial encounter |
| S06815D | ICD10 | Injury of right internal carotid artery, intracranial portion, not elsewhere classified with loss of consciousness greater than 24 hours with return to pre-existing conscious level, subsequent encounter |
| S06815S | ICD10 | Injury of right internal carotid artery, intracranial portion, not elsewhere classified with loss of consciousness greater than 24 hours with return to pre-existing conscious level, sequela |
| S06816A | ICD10 | Injury of right internal carotid artery, intracranial portion, not elsewhere classified with loss of consciousness greater than 24 hours without return to pre-existing conscious level with patient surviving, initial encounter |
| S079XXA | ICD10 | Crushing injury of head, part unspecified, initial encounter |
| S06816D | ICD10 | Injury of right internal carotid artery, intracranial portion, not elsewhere classified with loss of consciousness greater than 24 hours without return to pre-existing conscious level with patient surviving, subsequent encounter |
| S06816S | ICD10 | Injury of right internal carotid artery, intracranial portion, not elsewhere classified with loss of consciousness greater than 24 hours without return to pre-existing conscious level with patient surviving, sequela |
| S06817A | ICD10 | Injury of right internal carotid artery, intracranial portion, not elsewhere classified with loss of consciousness of any duration with death due to brain injury prior to regaining consciousness, initial encounter |
| S06818A | ICD10 | Injury of right internal carotid artery, intracranial portion, not elsewhere classified with loss of consciousness of any duration with death due to other cause prior to regaining consciousness, initial encounter |
| S06819A | ICD10 | Injury of right internal carotid artery, intracranial portion, not elsewhere classified with loss of consciousness of unspecified duration, initial encounter |
| S06819D | ICD10 | Injury of right internal carotid artery, intracranial portion, not elsewhere classified with loss of consciousness of unspecified duration, subsequent encounter |
| S06819S | ICD10 | Injury of right internal carotid artery, intracranial portion, not elsewhere classified with loss of consciousness of unspecified duration, sequela |
| S06820A | ICD10 | Injury of left internal carotid artery, intracranial portion, not elsewhere classified without loss of consciousness, initial encounter |
| S06820D | ICD10 | Injury of left internal carotid artery, intracranial portion, not elsewhere classified without loss of consciousness, subsequent encounter |
| S06820S | ICD10 | Injury of left internal carotid artery, intracranial portion, not elsewhere classified without loss of consciousness, sequela |
| S06821A | ICD10 | Injury of left internal carotid artery, intracranial portion, not elsewhere classified with loss of consciousness of 30 minutes or less, initial encounter |
| S06821D | ICD10 | Injury of left internal carotid artery, intracranial portion, not elsewhere classified with loss of consciousness of 30 minutes or less, subsequent encounter |
| S06821S | ICD10 | Injury of left internal carotid artery, intracranial portion, not elsewhere classified with loss of consciousness of 30 minutes or less, sequela |
| S06822A | ICD10 | Injury of left internal carotid artery, intracranial portion, not elsewhere classified with loss of consciousness of 31 minutes to 59 minutes, initial encounter |
| S06822D | ICD10 | Injury of left internal carotid artery, intracranial portion, not elsewhere classified with loss of consciousness of 31 minutes to 59 minutes, subsequent encounter |
| S06822S | ICD10 | Injury of left internal carotid artery, intracranial portion, not elsewhere classified with loss of consciousness of 31 minutes to 59 minutes, sequela |
| S06823A | ICD10 | Injury of left internal carotid artery, intracranial portion, not elsewhere classified with loss of consciousness of 1 hour to 5 hours 59 minutes, initial encounter |
| S06823D | ICD10 | Injury of left internal carotid artery, intracranial portion, not elsewhere classified with loss of consciousness of 1 hour to 5 hours 59 minutes, subsequent encounter |
| S06823S | ICD10 | Injury of left internal carotid artery, intracranial portion, not elsewhere classified with loss of consciousness of 1 hour to 5 hours 59 minutes, sequela |
| S06824A | ICD10 | Injury of left internal carotid artery, intracranial portion, not elsewhere classified with loss of consciousness of 6 hours to 24 hours, initial encounter |
| S06824D | ICD10 | Injury of left internal carotid artery, intracranial portion, not elsewhere classified with loss of consciousness of 6 hours to 24 hours, subsequent encounter |
| S06824S | ICD10 | Injury of left internal carotid artery, intracranial portion, not elsewhere classified with loss of consciousness of 6 hours to 24 hours, sequela |
| S06825A | ICD10 | Injury of left internal carotid artery, intracranial portion, not elsewhere classified with loss of consciousness greater than 24 hours with return to pre-existing conscious level, initial encounter |
| S06825D | ICD10 | Injury of left internal carotid artery, intracranial portion, not elsewhere classified with loss of consciousness greater than 24 hours with return to pre-existing conscious level, subsequent encounter |
| S06825S | ICD10 | Injury of left internal carotid artery, intracranial portion, not elsewhere classified with loss of consciousness greater than 24 hours with return to pre-existing conscious level, sequela |
| S06826A | ICD10 | Injury of left internal carotid artery, intracranial portion, not elsewhere classified with loss of consciousness greater than 24 hours without return to pre-existing conscious level with patient surviving, initial encounter |
| S06826D | ICD10 | Injury of left internal carotid artery, intracranial portion, not elsewhere classified with loss of consciousness greater than 24 hours without return to pre-existing conscious level with patient surviving, subsequent encounter |
| S06826S | ICD10 | Injury of left internal carotid artery, intracranial portion, not elsewhere classified with loss of consciousness greater than 24 hours without return to pre-existing conscious level with patient surviving, sequela |
| S06827A | ICD10 | Injury of left internal carotid artery, intracranial portion, not elsewhere classified with loss of consciousness of any duration with death due to brain injury prior to regaining consciousness, initial encounter |
| S06828A | ICD10 | Injury of left internal carotid artery, intracranial portion, not elsewhere classified with loss of consciousness of any duration with death due to other cause prior to regaining consciousness, initial encounter |
| S06829A | ICD10 | Injury of left internal carotid artery, intracranial portion, not elsewhere classified with loss of consciousness of unspecified duration, initial encounter |
| S06829D | ICD10 | Injury of left internal carotid artery, intracranial portion, not elsewhere classified with loss of consciousness of unspecified duration, subsequent encounter |
| S06829S | ICD10 | Injury of left internal carotid artery, intracranial portion, not elsewhere classified with loss of consciousness of unspecified duration, sequela |
| S06890A | ICD10 | Other specified intracranial injury without loss of consciousness, initial encounter |
| S06890D | ICD10 | Other specified intracranial injury without loss of consciousness, subsequent encounter |
| S06890S | ICD10 | Other specified intracranial injury without loss of consciousness, sequela |
| S06891A | ICD10 | Other specified intracranial injury with loss of consciousness of 30 minutes or less, initial encounter |
| S06891D | ICD10 | Other specified intracranial injury with loss of consciousness of 30 minutes or less, subsequent encounter |
| S06891S | ICD10 | Other specified intracranial injury with loss of consciousness of 30 minutes or less, sequela |
| W880XXS | ICD10 | Exposure to X-rays, sequela |
| S06892A | ICD10 | Other specified intracranial injury with loss of consciousness of 31 minutes to 59 minutes, initial encounter |
| S06892D | ICD10 | Other specified intracranial injury with loss of consciousness of 31 minutes to 59 minutes, subsequent encounter |
| S06892S | ICD10 | Other specified intracranial injury with loss of consciousness of 31 minutes to 59 minutes, sequela |
| S06893A | ICD10 | Other specified intracranial injury with loss of consciousness of 1 hour to 5 hours 59 minutes, initial encounter |
| S06893D | ICD10 | Other specified intracranial injury with loss of consciousness of 1 hour to 5 hours 59 minutes, subsequent encounter |
| S06893S | ICD10 | Other specified intracranial injury with loss of consciousness of 1 hour to 5 hours 59 minutes, sequela |
| S06894A | ICD10 | Other specified intracranial injury with loss of consciousness of 6 hours to 24 hours, initial encounter |
| S06894D | ICD10 | Other specified intracranial injury with loss of consciousness of 6 hours to 24 hours, subsequent encounter |
| S06894S | ICD10 | Other specified intracranial injury with loss of consciousness of 6 hours to 24 hours, sequela |
| S06895A | ICD10 | Other specified intracranial injury with loss of consciousness greater than 24 hours with return to pre-existing conscious level, initial encounter |
| S06895D | ICD10 | Other specified intracranial injury with loss of consciousness greater than 24 hours with return to pre-existing conscious level, subsequent encounter |
| S06895S | ICD10 | Other specified intracranial injury with loss of consciousness greater than 24 hours with return to pre-existing conscious level, sequela |
| S06896A | ICD10 | Other specified intracranial injury with loss of consciousness greater than 24 hours without return to pre-existing conscious level with patient surviving, initial encounter |
| S06896D | ICD10 | Other specified intracranial injury with loss of consciousness greater than 24 hours without return to pre-existing conscious level with patient surviving, subsequent encounter |
| S06896S | ICD10 | Other specified intracranial injury with loss of consciousness greater than 24 hours without return to pre-existing conscious level with patient surviving, sequela |
| S06897A | ICD10 | Other specified intracranial injury with loss of consciousness of any duration with death due to brain injury prior to regaining consciousness, initial encounter |
| S06898A | ICD10 | Other specified intracranial injury with loss of consciousness of any duration with death due to other cause prior to regaining consciousness, initial encounter |
| S06899A | ICD10 | Other specified intracranial injury with loss of consciousness of unspecified duration, initial encounter |
| S06899D | ICD10 | Other specified intracranial injury with loss of consciousness of unspecified duration, subsequent encounter |
| S06899S | ICD10 | Other specified intracranial injury with loss of consciousness of unspecified duration, sequela |
| S069X0A | ICD10 | Unspecified intracranial injury without loss of consciousness, initial encounter |
| S069X0D | ICD10 | Unspecified intracranial injury without loss of consciousness, subsequent encounter |
| S069X0S | ICD10 | Unspecified intracranial injury without loss of consciousness, sequela |
| S069X1A | ICD10 | Unspecified intracranial injury with loss of consciousness of 30 minutes or less, initial encounter |
| S069X1D | ICD10 | Unspecified intracranial injury with loss of consciousness of 30 minutes or less, subsequent encounter |
| S069X1S | ICD10 | Unspecified intracranial injury with loss of consciousness of 30 minutes or less, sequela |
| S069X2A | ICD10 | Unspecified intracranial injury with loss of consciousness of 31 minutes to 59 minutes, initial encounter |
| S069X2D | ICD10 | Unspecified intracranial injury with loss of consciousness of 31 minutes to 59 minutes, subsequent encounter |
| S069X2S | ICD10 | Unspecified intracranial injury with loss of consciousness of 31 minutes to 59 minutes, sequela |
| S069X3A | ICD10 | Unspecified intracranial injury with loss of consciousness of 1 hour to 5 hours 59 minutes, initial encounter |
| S069X3D | ICD10 | Unspecified intracranial injury with loss of consciousness of 1 hour to 5 hours 59 minutes, subsequent encounter |
| S069X3S | ICD10 | Unspecified intracranial injury with loss of consciousness of 1 hour to 5 hours 59 minutes, sequela |
| S069X4A | ICD10 | Unspecified intracranial injury with loss of consciousness of 6 hours to 24 hours, initial encounter |
| S069X4D | ICD10 | Unspecified intracranial injury with loss of consciousness of 6 hours to 24 hours, subsequent encounter |
| S069X4S | ICD10 | Unspecified intracranial injury with loss of consciousness of 6 hours to 24 hours, sequela |
| S069X5A | ICD10 | Unspecified intracranial injury with loss of consciousness greater than 24 hours with return to pre-existing conscious level, initial encounter |
| S069X5D | ICD10 | Unspecified intracranial injury with loss of consciousness greater than 24 hours with return to pre-existing conscious level, subsequent encounter |
| S069X5S | ICD10 | Unspecified intracranial injury with loss of consciousness greater than 24 hours with return to pre-existing conscious level, sequela |
| S069X6A | ICD10 | Unspecified intracranial injury with loss of consciousness greater than 24 hours without return to pre-existing conscious level with patient surviving, initial encounter |
| S069X6D | ICD10 | Unspecified intracranial injury with loss of consciousness greater than 24 hours without return to pre-existing conscious level with patient surviving, subsequent encounter |
| S069X6S | ICD10 | Unspecified intracranial injury with loss of consciousness greater than 24 hours without return to pre-existing conscious level with patient surviving, sequela |
| S069X7A | ICD10 | Unspecified intracranial injury with loss of consciousness of any duration with death due to brain injury prior to regaining consciousness, initial encounter |
| S069X8A | ICD10 | Unspecified intracranial injury with loss of consciousness of any duration with death due to other cause prior to regaining consciousness, initial encounter |
| S069X9A | ICD10 | Unspecified intracranial injury with loss of consciousness of unspecified duration, initial encounter |
| S069X9D | ICD10 | Unspecified intracranial injury with loss of consciousness of unspecified duration, subsequent encounter |
| S069X9S | ICD10 | Unspecified intracranial injury with loss of consciousness of unspecified duration, sequela |
| S070XXA | ICD10 | Crushing injury of face, initial encounter |
| S070XXD | ICD10 | Crushing injury of face, subsequent encounter |
| S070XXS | ICD10 | Crushing injury of face, sequela |
| S071XXA | ICD10 | Crushing injury of skull, initial encounter |
| S071XXD | ICD10 | Crushing injury of skull, subsequent encounter |
| S071XXS | ICD10 | Crushing injury of skull, sequela |
| S078XXA | ICD10 | Crushing injury of other parts of head, initial encounter |
| S079XXD | ICD10 | Crushing injury of head, part unspecified, subsequent encounter |
| S079XXS | ICD10 | Crushing injury of head, part unspecified, sequela |
| S080XXA | ICD10 | Avulsion of scalp, initial encounter |
| S080XXD | ICD10 | Avulsion of scalp, subsequent encounter |
| S080XXS | ICD10 | Avulsion of scalp, sequela |
| S08111A | ICD10 | Complete traumatic amputation of right ear, initial encounter |
| S08111D | ICD10 | Complete traumatic amputation of right ear, subsequent encounter |
| S08111S | ICD10 | Complete traumatic amputation of right ear, sequela |
| S08112A | ICD10 | Complete traumatic amputation of left ear, initial encounter |
| S08112D | ICD10 | Complete traumatic amputation of left ear, subsequent encounter |
| S08112S | ICD10 | Complete traumatic amputation of left ear, sequela |
| S08119A | ICD10 | Complete traumatic amputation of unspecified ear, initial encounter |
| S08119D | ICD10 | Complete traumatic amputation of unspecified ear, subsequent encounter |
| S08119S | ICD10 | Complete traumatic amputation of unspecified ear, sequela |
| S08121A | ICD10 | Partial traumatic amputation of right ear, initial encounter |
| S08121D | ICD10 | Partial traumatic amputation of right ear, subsequent encounter |
| S08121S | ICD10 | Partial traumatic amputation of right ear, sequela |
| S08122A | ICD10 | Partial traumatic amputation of left ear, initial encounter |
| S08122D | ICD10 | Partial traumatic amputation of left ear, subsequent encounter |
| S08122S | ICD10 | Partial traumatic amputation of left ear, sequela |
| S08129A | ICD10 | Partial traumatic amputation of unspecified ear, initial encounter |
| S08129D | ICD10 | Partial traumatic amputation of unspecified ear, subsequent encounter |
| S08129S | ICD10 | Partial traumatic amputation of unspecified ear, sequela |
| S08811A | ICD10 | Complete traumatic amputation of nose, initial encounter |
| S08811D | ICD10 | Complete traumatic amputation of nose, subsequent encounter |
| S08811S | ICD10 | Complete traumatic amputation of nose, sequela |
| S08812A | ICD10 | Partial traumatic amputation of nose, initial encounter |
| S08812D | ICD10 | Partial traumatic amputation of nose, subsequent encounter |
| S08812S | ICD10 | Partial traumatic amputation of nose, sequela |
| S0889XA | ICD10 | Traumatic amputation of other parts of head, initial encounter |
| S0889XD | ICD10 | Traumatic amputation of other parts of head, subsequent encounter |
| S0889XS | ICD10 | Traumatic amputation of other parts of head, sequela |
| S090XXA | ICD10 | Injury of blood vessels of head, not elsewhere classified, initial encounter |
| S090XXD | ICD10 | Injury of blood vessels of head, not elsewhere classified, subsequent encounter |
| S090XXS | ICD10 | Injury of blood vessels of head, not elsewhere classified, sequela |
| S0280XA | ICD10 | Fracture of other specified skull and facial bones, unspecified side, initial encounter for closed fracture |
| S0280XB | ICD10 | Fracture of other specified skull and facial bones, unspecified side, initial encounter for open fracture |
| S0280XD | ICD10 | Fracture of other specified skull and facial bones, unspecified side, subsequent encounter for fracture with routine healing |
| S0280XG | ICD10 | Fracture of other specified skull and facial bones, unspecified side, subsequent encounter for fracture with delayed healing |
| S0280XK | ICD10 | Fracture of other specified skull and facial bones, unspecified side, subsequent encounter for fracture with nonunion |
| S0280XS | ICD10 | Fracture of other specified skull and facial bones, unspecified side, sequela |
| S0281XA | ICD10 | Fracture of other specified skull and facial bones, right side, initial encounter for closed fracture |
| S0281XB | ICD10 | Fracture of other specified skull and facial bones, right side, initial encounter for open fracture |
| S0291XA | ICD10 | Unspecified fracture of skull, initial encounter for closed fracture |
| S0291XB | ICD10 | Unspecified fracture of skull, initial encounter for open fracture |
| S0291XD | ICD10 | Unspecified fracture of skull, subsequent encounter for fracture with routine healing |
| S0291XG | ICD10 | Unspecified fracture of skull, subsequent encounter for fracture with delayed healing |
| S0291XK | ICD10 | Unspecified fracture of skull, subsequent encounter for fracture with nonunion |
| S0291XS | ICD10 | Unspecified fracture of skull, sequela |
| S060X1S | ICD10 | Concussion with loss of consciousness of 30 minutes or less, sequela |
| S060X9A | ICD10 | Concussion with loss of consciousness of unspecified duration, initial encounter |
| S060X9D | ICD10 | Concussion with loss of consciousness of unspecified duration, subsequent encounter |
| S060X9S | ICD10 | Concussion with loss of consciousness of unspecified duration, sequela |
| S061X0A | ICD10 | Traumatic cerebral edema without loss of consciousness, initial encounter |
| S061X0D | ICD10 | Traumatic cerebral edema without loss of consciousness, subsequent encounter |
| S061X0S | ICD10 | Traumatic cerebral edema without loss of consciousness, sequela |
| S061X1A | ICD10 | Traumatic cerebral edema with loss of consciousness of 30 minutes or less, initial encounter |
| S061X1D | ICD10 | Traumatic cerebral edema with loss of consciousness of 30 minutes or less, subsequent encounter |
| S061X1S | ICD10 | Traumatic cerebral edema with loss of consciousness of 30 minutes or less, sequela |
| S061X2A | ICD10 | Traumatic cerebral edema with loss of consciousness of 31 minutes to 59 minutes, initial encounter |
| S061X2D | ICD10 | Traumatic cerebral edema with loss of consciousness of 31 minutes to 59 minutes, subsequent encounter |
| S061X2S | ICD10 | Traumatic cerebral edema with loss of consciousness of 31 minutes to 59 minutes, sequela |
| S061X3A | ICD10 | Traumatic cerebral edema with loss of consciousness of 1 hour to 5 hours 59 minutes, initial encounter |
| S061X3D | ICD10 | Traumatic cerebral edema with loss of consciousness of 1 hour to 5 hours 59 minutes, subsequent encounter |
| S061X3S | ICD10 | Traumatic cerebral edema with loss of consciousness of 1 hour to 5 hours 59 minutes, sequela |
| S061X4A | ICD10 | Traumatic cerebral edema with loss of consciousness of 6 hours to 24 hours, initial encounter |
| S061X4D | ICD10 | Traumatic cerebral edema with loss of consciousness of 6 hours to 24 hours, subsequent encounter |
| S061X4S | ICD10 | Traumatic cerebral edema with loss of consciousness of 6 hours to 24 hours, sequela |
| S061X5A | ICD10 | Traumatic cerebral edema with loss of consciousness greater than 24 hours with return to pre-existing conscious level, initial encounter |
| S061X5D | ICD10 | Traumatic cerebral edema with loss of consciousness greater than 24 hours with return to pre-existing conscious level, subsequent encounter |
| S061X5S | ICD10 | Traumatic cerebral edema with loss of consciousness greater than 24 hours with return to pre-existing conscious level, sequela |
| S061X6A | ICD10 | Traumatic cerebral edema with loss of consciousness greater than 24 hours without return to pre-existing conscious level with patient surviving, initial encounter |
| S061X6D | ICD10 | Traumatic cerebral edema with loss of consciousness greater than 24 hours without return to pre-existing conscious level with patient surviving, subsequent encounter |
| S061X6S | ICD10 | Traumatic cerebral edema with loss of consciousness greater than 24 hours without return to pre-existing conscious level with patient surviving, sequela |
| S061X7A | ICD10 | Traumatic cerebral edema with loss of consciousness of any duration with death due to brain injury prior to regaining consciousness, initial encounter |
| S061X8A | ICD10 | Traumatic cerebral edema with loss of consciousness of any duration with death due to other cause prior to regaining consciousness, initial encounter |
| S061X9A | ICD10 | Traumatic cerebral edema with loss of consciousness of unspecified duration, initial encounter |
| S061X9D | ICD10 | Traumatic cerebral edema with loss of consciousness of unspecified duration, subsequent encounter |
| S061X9S | ICD10 | Traumatic cerebral edema with loss of consciousness of unspecified duration, sequela |
| S062X0A | ICD10 | Diffuse traumatic brain injury without loss of consciousness, initial encounter |
| S062X0D | ICD10 | Diffuse traumatic brain injury without loss of consciousness, subsequent encounter |
| S062X0S | ICD10 | Diffuse traumatic brain injury without loss of consciousness, sequela |
| S062X1A | ICD10 | Diffuse traumatic brain injury with loss of consciousness of 30 minutes or less, initial encounter |
| S062X1D | ICD10 | Diffuse traumatic brain injury with loss of consciousness of 30 minutes or less, subsequent encounter |
| S062X1S | ICD10 | Diffuse traumatic brain injury with loss of consciousness of 30 minutes or less, sequela |
| S062X2A | ICD10 | Diffuse traumatic brain injury with loss of consciousness of 31 minutes to 59 minutes, initial encounter |
| S062X2D | ICD10 | Diffuse traumatic brain injury with loss of consciousness of 31 minutes to 59 minutes, subsequent encounter |
| S062X2S | ICD10 | Diffuse traumatic brain injury with loss of consciousness of 31 minutes to 59 minutes, sequela |
| S062X3A | ICD10 | Diffuse traumatic brain injury with loss of consciousness of 1 hour to 5 hours 59 minutes, initial encounter |
| S062X3D | ICD10 | Diffuse traumatic brain injury with loss of consciousness of 1 hour to 5 hours 59 minutes, subsequent encounter |
| S062X3S | ICD10 | Diffuse traumatic brain injury with loss of consciousness of 1 hour to 5 hours 59 minutes, sequela |
| S062X4A | ICD10 | Diffuse traumatic brain injury with loss of consciousness of 6 hours to 24 hours, initial encounter |
| S062X4D | ICD10 | Diffuse traumatic brain injury with loss of consciousness of 6 hours to 24 hours, subsequent encounter |
| S062X4S | ICD10 | Diffuse traumatic brain injury with loss of consciousness of 6 hours to 24 hours, sequela |
| S062X5A | ICD10 | Diffuse traumatic brain injury with loss of consciousness greater than 24 hours with return to pre-existing conscious levels, initial encounter |
| S062X5D | ICD10 | Diffuse traumatic brain injury with loss of consciousness greater than 24 hours with return to pre-existing conscious levels, subsequent encounter |
| S062X5S | ICD10 | Diffuse traumatic brain injury with loss of consciousness greater than 24 hours with return to pre-existing conscious levels, sequela |
| S062X6A | ICD10 | Diffuse traumatic brain injury with loss of consciousness greater than 24 hours without return to pre-existing conscious level with patient surviving, initial encounter |
| S062X6D | ICD10 | Diffuse traumatic brain injury with loss of consciousness greater than 24 hours without return to pre-existing conscious level with patient surviving, subsequent encounter |
| S062X6S | ICD10 | Diffuse traumatic brain injury with loss of consciousness greater than 24 hours without return to pre-existing conscious level with patient surviving, sequela |
| S062X7A | ICD10 | Diffuse traumatic brain injury with loss of consciousness of any duration with death due to brain injury prior to regaining consciousness, initial encounter |
| S062X8A | ICD10 | Diffuse traumatic brain injury with loss of consciousness of any duration with death due to other cause prior to regaining consciousness, initial encounter |
| V1542 | ICD10 | History of emotional abuse |
| S062X9A | ICD10 | Diffuse traumatic brain injury with loss of consciousness of unspecified duration, initial encounter |
| S062X9D | ICD10 | Diffuse traumatic brain injury with loss of consciousness of unspecified duration, subsequent encounter |
| S062X9S | ICD10 | Diffuse traumatic brain injury with loss of consciousness of unspecified duration, sequela |
| S06300A | ICD10 | Unspecified focal traumatic brain injury without loss of consciousness, initial encounter |
| S06300D | ICD10 | Unspecified focal traumatic brain injury without loss of consciousness, subsequent encounter |
| S06300S | ICD10 | Unspecified focal traumatic brain injury without loss of consciousness, sequela |
| S06301A | ICD10 | Unspecified focal traumatic brain injury with loss of consciousness of 30 minutes or less, initial encounter |
| S06301D | ICD10 | Unspecified focal traumatic brain injury with loss of consciousness of 30 minutes or less, subsequent encounter |
| S06301S | ICD10 | Unspecified focal traumatic brain injury with loss of consciousness of 30 minutes or less, sequela |
| S06302A | ICD10 | Unspecified focal traumatic brain injury with loss of consciousness of 31 minutes to 59 minutes, initial encounter |
| S06302D | ICD10 | Unspecified focal traumatic brain injury with loss of consciousness of 31 minutes to 59 minutes, subsequent encounter |
| S06302S | ICD10 | Unspecified focal traumatic brain injury with loss of consciousness of 31 minutes to 59 minutes, sequela |
| S06303A | ICD10 | Unspecified focal traumatic brain injury with loss of consciousness of 1 hour to 5 hours 59 minutes, initial encounter |
| S06303D | ICD10 | Unspecified focal traumatic brain injury with loss of consciousness of 1 hour to 5 hours 59 minutes, subsequent encounter |
| S06303S | ICD10 | Unspecified focal traumatic brain injury with loss of consciousness of 1 hour to 5 hours 59 minutes, sequela |
| S06304A | ICD10 | Unspecified focal traumatic brain injury with loss of consciousness of 6 hours to 24 hours, initial encounter |
| S06304D | ICD10 | Unspecified focal traumatic brain injury with loss of consciousness of 6 hours to 24 hours, subsequent encounter |
| S06304S | ICD10 | Unspecified focal traumatic brain injury with loss of consciousness of 6 hours to 24 hours, sequela |
| S06305A | ICD10 | Unspecified focal traumatic brain injury with loss of consciousness greater than 24 hours with return to pre-existing conscious level, initial encounter |
| S06305D | ICD10 | Unspecified focal traumatic brain injury with loss of consciousness greater than 24 hours with return to pre-existing conscious level, subsequent encounter |
| S06305S | ICD10 | Unspecified focal traumatic brain injury with loss of consciousness greater than 24 hours with return to pre-existing conscious level, sequela |
| S06306A | ICD10 | Unspecified focal traumatic brain injury with loss of consciousness greater than 24 hours without return to pre-existing conscious level with patient surviving, initial encounter |
| S06306D | ICD10 | Unspecified focal traumatic brain injury with loss of consciousness greater than 24 hours without return to pre-existing conscious level with patient surviving, subsequent encounter |
| S06306S | ICD10 | Unspecified focal traumatic brain injury with loss of consciousness greater than 24 hours without return to pre-existing conscious level with patient surviving, sequela |
| S06307A | ICD10 | Unspecified focal traumatic brain injury with loss of consciousness of any duration with death due to brain injury prior to regaining consciousness, initial encounter |
| S06308A | ICD10 | Unspecified focal traumatic brain injury with loss of consciousness of any duration with death due to other cause prior to regaining consciousness, initial encounter |
| S06309A | ICD10 | Unspecified focal traumatic brain injury with loss of consciousness of unspecified duration, initial encounter |
| S06309D | ICD10 | Unspecified focal traumatic brain injury with loss of consciousness of unspecified duration, subsequent encounter |
| S06309S | ICD10 | Unspecified focal traumatic brain injury with loss of consciousness of unspecified duration, sequela |
| S06310A | ICD10 | Contusion and laceration of right cerebrum without loss of consciousness, initial encounter |
| S06310D | ICD10 | Contusion and laceration of right cerebrum without loss of consciousness, subsequent encounter |
| S06310S | ICD10 | Contusion and laceration of right cerebrum without loss of consciousness, sequela |
| S06311A | ICD10 | Contusion and laceration of right cerebrum with loss of consciousness of 30 minutes or less, initial encounter |
| S06311D | ICD10 | Contusion and laceration of right cerebrum with loss of consciousness of 30 minutes or less, subsequent encounter |
| S06311S | ICD10 | Contusion and laceration of right cerebrum with loss of consciousness of 30 minutes or less, sequela |
| S06312A | ICD10 | Contusion and laceration of right cerebrum with loss of consciousness of 31 minutes to 59 minutes, initial encounter |
| S06312D | ICD10 | Contusion and laceration of right cerebrum with loss of consciousness of 31 minutes to 59 minutes, subsequent encounter |
| S06312S | ICD10 | Contusion and laceration of right cerebrum with loss of consciousness of 31 minutes to 59 minutes, sequela |
| S06313A | ICD10 | Contusion and laceration of right cerebrum with loss of consciousness of 1 hour to 5 hours 59 minutes, initial encounter |
| S06313D | ICD10 | Contusion and laceration of right cerebrum with loss of consciousness of 1 hour to 5 hours 59 minutes, subsequent encounter |
| S06313S | ICD10 | Contusion and laceration of right cerebrum with loss of consciousness of 1 hour to 5 hours 59 minutes, sequela |
| S06314A | ICD10 | Contusion and laceration of right cerebrum with loss of consciousness of 6 hours to 24 hours, initial encounter |
| S06314D | ICD10 | Contusion and laceration of right cerebrum with loss of consciousness of 6 hours to 24 hours, subsequent encounter |
| S06314S | ICD10 | Contusion and laceration of right cerebrum with loss of consciousness of 6 hours to 24 hours, sequela |
| S06315A | ICD10 | Contusion and laceration of right cerebrum with loss of consciousness greater than 24 hours with return to pre-existing conscious level, initial encounter |
| S06315D | ICD10 | Contusion and laceration of right cerebrum with loss of consciousness greater than 24 hours with return to pre-existing conscious level, subsequent encounter |
| S06315S | ICD10 | Contusion and laceration of right cerebrum with loss of consciousness greater than 24 hours with return to pre-existing conscious level, sequela |
| S06316A | ICD10 | Contusion and laceration of right cerebrum with loss of consciousness greater than 24 hours without return to pre-existing conscious level with patient surviving, initial encounter |
| S06316D | ICD10 | Contusion and laceration of right cerebrum with loss of consciousness greater than 24 hours without return to pre-existing conscious level with patient surviving, subsequent encounter |
| S078XXD | ICD10 | Crushing injury of other parts of head, subsequent encounter |
| S06316S | ICD10 | Contusion and laceration of right cerebrum with loss of consciousness greater than 24 hours without return to pre-existing conscious level with patient surviving, sequela |
| S06317A | ICD10 | Contusion and laceration of right cerebrum with loss of consciousness of any duration with death due to brain injury prior to regaining consciousness, initial encounter |
| S06318A | ICD10 | Contusion and laceration of right cerebrum with loss of consciousness of any duration with death due to other cause prior to regaining consciousness, initial encounter |
| S06319A | ICD10 | Contusion and laceration of right cerebrum with loss of consciousness of unspecified duration, initial encounter |
| S06319D | ICD10 | Contusion and laceration of right cerebrum with loss of consciousness of unspecified duration, subsequent encounter |
| S06319S | ICD10 | Contusion and laceration of right cerebrum with loss of consciousness of unspecified duration, sequela |
| S06320A | ICD10 | Contusion and laceration of left cerebrum without loss of consciousness, initial encounter |
| S06320D | ICD10 | Contusion and laceration of left cerebrum without loss of consciousness, subsequent encounter |
| S06320S | ICD10 | Contusion and laceration of left cerebrum without loss of consciousness, sequela |
| S06321A | ICD10 | Contusion and laceration of left cerebrum with loss of consciousness of 30 minutes or less, initial encounter |
| S06321D | ICD10 | Contusion and laceration of left cerebrum with loss of consciousness of 30 minutes or less, subsequent encounter |
| S06321S | ICD10 | Contusion and laceration of left cerebrum with loss of consciousness of 30 minutes or less, sequela |
| S06322A | ICD10 | Contusion and laceration of left cerebrum with loss of consciousness of 31 minutes to 59 minutes, initial encounter |
| S06322D | ICD10 | Contusion and laceration of left cerebrum with loss of consciousness of 31 minutes to 59 minutes, subsequent encounter |
| S06322S | ICD10 | Contusion and laceration of left cerebrum with loss of consciousness of 31 minutes to 59 minutes, sequela |
| S06323A | ICD10 | Contusion and laceration of left cerebrum with loss of consciousness of 1 hour to 5 hours 59 minutes, initial encounter |
| S06323D | ICD10 | Contusion and laceration of left cerebrum with loss of consciousness of 1 hour to 5 hours 59 minutes, subsequent encounter |
| S06323S | ICD10 | Contusion and laceration of left cerebrum with loss of consciousness of 1 hour to 5 hours 59 minutes, sequela |
| S06324A | ICD10 | Contusion and laceration of left cerebrum with loss of consciousness of 6 hours to 24 hours, initial encounter |
| S06324D | ICD10 | Contusion and laceration of left cerebrum with loss of consciousness of 6 hours to 24 hours, subsequent encounter |
| S06324S | ICD10 | Contusion and laceration of left cerebrum with loss of consciousness of 6 hours to 24 hours, sequela |
| S06325A | ICD10 | Contusion and laceration of left cerebrum with loss of consciousness greater than 24 hours with return to pre-existing conscious level, initial encounter |
| S06325D | ICD10 | Contusion and laceration of left cerebrum with loss of consciousness greater than 24 hours with return to pre-existing conscious level, subsequent encounter |
| S06325S | ICD10 | Contusion and laceration of left cerebrum with loss of consciousness greater than 24 hours with return to pre-existing conscious level, sequela |
| S06326A | ICD10 | Contusion and laceration of left cerebrum with loss of consciousness greater than 24 hours without return to pre-existing conscious level with patient surviving, initial encounter |
| S06326D | ICD10 | Contusion and laceration of left cerebrum with loss of consciousness greater than 24 hours without return to pre-existing conscious level with patient surviving, subsequent encounter |
| S06326S | ICD10 | Contusion and laceration of left cerebrum with loss of consciousness greater than 24 hours without return to pre-existing conscious level with patient surviving, sequela |
| S06327A | ICD10 | Contusion and laceration of left cerebrum with loss of consciousness of any duration with death due to brain injury prior to regaining consciousness, initial encounter |
| S06328A | ICD10 | Contusion and laceration of left cerebrum with loss of consciousness of any duration with death due to other cause prior to regaining consciousness, initial encounter |
| S06329A | ICD10 | Contusion and laceration of left cerebrum with loss of consciousness of unspecified duration, initial encounter |
| S06329D | ICD10 | Contusion and laceration of left cerebrum with loss of consciousness of unspecified duration, subsequent encounter |
| S06329S | ICD10 | Contusion and laceration of left cerebrum with loss of consciousness of unspecified duration, sequela |
| S06330A | ICD10 | Contusion and laceration of cerebrum, unspecified, without loss of consciousness, initial encounter |
| S06330D | ICD10 | Contusion and laceration of cerebrum, unspecified, without loss of consciousness, subsequent encounter |
| S06330S | ICD10 | Contusion and laceration of cerebrum, unspecified, without loss of consciousness, sequela |
| S06331A | ICD10 | Contusion and laceration of cerebrum, unspecified, with loss of consciousness of 30 minutes or less, initial encounter |
| S06331D | ICD10 | Contusion and laceration of cerebrum, unspecified, with loss of consciousness of 30 minutes or less, subsequent encounter |
| S06331S | ICD10 | Contusion and laceration of cerebrum, unspecified, with loss of consciousness of 30 minutes or less, sequela |
| S06332A | ICD10 | Contusion and laceration of cerebrum, unspecified, with loss of consciousness of 31 minutes to 59 minutes, initial encounter |
| S06332D | ICD10 | Contusion and laceration of cerebrum, unspecified, with loss of consciousness of 31 minutes to 59 minutes, subsequent encounter |
| S06332S | ICD10 | Contusion and laceration of cerebrum, unspecified, with loss of consciousness of 31 minutes to 59 minutes, sequela |
| S06333A | ICD10 | Contusion and laceration of cerebrum, unspecified, with loss of consciousness of 1 hour to 5 hours 59 minutes, initial encounter |
| S06333D | ICD10 | Contusion and laceration of cerebrum, unspecified, with loss of consciousness of 1 hour to 5 hours 59 minutes, subsequent encounter |
| S06334A | ICD10 | Contusion and laceration of cerebrum, unspecified, with loss of consciousness of 6 hours to 24 hours, initial encounter |
| S06334D | ICD10 | Contusion and laceration of cerebrum, unspecified, with loss of consciousness of 6 hours to 24 hours, subsequent encounter |
| S06334S | ICD10 | Contusion and laceration of cerebrum, unspecified, with loss of consciousness of 6 hours to 24 hours, sequela |
| S06335A | ICD10 | Contusion and laceration of cerebrum, unspecified, with loss of consciousness greater than 24 hours with return to pre-existing conscious level, initial encounter |
| S06335D | ICD10 | Contusion and laceration of cerebrum, unspecified, with loss of consciousness greater than 24 hours with return to pre-existing conscious level, subsequent encounter |
| S06335S | ICD10 | Contusion and laceration of cerebrum, unspecified, with loss of consciousness greater than 24 hours with return to pre-existing conscious level, sequela |
| S06336A | ICD10 | Contusion and laceration of cerebrum, unspecified, with loss of consciousness greater than 24 hours without return to pre-existing conscious level with patient surviving, initial encounter |
| S06336D | ICD10 | Contusion and laceration of cerebrum, unspecified, with loss of consciousness greater than 24 hours without return to pre-existing conscious level with patient surviving, subsequent encounter |
| S06336S | ICD10 | Contusion and laceration of cerebrum, unspecified, with loss of consciousness greater than 24 hours without return to pre-existing conscious level with patient surviving, sequela |
| S06337A | ICD10 | Contusion and laceration of cerebrum, unspecified, with loss of consciousness of any duration with death due to brain injury prior to regaining consciousness, initial encounter |
| S06338A | ICD10 | Contusion and laceration of cerebrum, unspecified, with loss of consciousness of any duration with death due to other cause prior to regaining consciousness, initial encounter |
| S06339A | ICD10 | Contusion and laceration of cerebrum, unspecified, with loss of consciousness of unspecified duration, initial encounter |
| S06339D | ICD10 | Contusion and laceration of cerebrum, unspecified, with loss of consciousness of unspecified duration, subsequent encounter |
| S06339S | ICD10 | Contusion and laceration of cerebrum, unspecified, with loss of consciousness of unspecified duration, sequela |
| S06340A | ICD10 | Traumatic hemorrhage of right cerebrum without loss of consciousness, initial encounter |
| S06340D | ICD10 | Traumatic hemorrhage of right cerebrum without loss of consciousness, subsequent encounter |
| S06340S | ICD10 | Traumatic hemorrhage of right cerebrum without loss of consciousness, sequela |
| S06341A | ICD10 | Traumatic hemorrhage of right cerebrum with loss of consciousness of 30 minutes or less, initial encounter |
| S06341D | ICD10 | Traumatic hemorrhage of right cerebrum with loss of consciousness of 30 minutes or less, subsequent encounter |
| S06341S | ICD10 | Traumatic hemorrhage of right cerebrum with loss of consciousness of 30 minutes or less, sequela |
| S06342A | ICD10 | Traumatic hemorrhage of right cerebrum with loss of consciousness of 31 minutes to 59 minutes, initial encounter |
| S06342D | ICD10 | Traumatic hemorrhage of right cerebrum with loss of consciousness of 31 minutes to 59 minutes, subsequent encounter |
| S06342S | ICD10 | Traumatic hemorrhage of right cerebrum with loss of consciousness of 31 minutes to 59 minutes, sequela |
| S06343A | ICD10 | Traumatic hemorrhage of right cerebrum with loss of consciousness of 1 hours to 5 hours 59 minutes, initial encounter |
| S06343D | ICD10 | Traumatic hemorrhage of right cerebrum with loss of consciousness of 1 hours to 5 hours 59 minutes, subsequent encounter |
| S06343S | ICD10 | Traumatic hemorrhage of right cerebrum with loss of consciousness of 1 hours to 5 hours 59 minutes, sequela |
| S06344A | ICD10 | Traumatic hemorrhage of right cerebrum with loss of consciousness of 6 hours to 24 hours, initial encounter |
| S06344D | ICD10 | Traumatic hemorrhage of right cerebrum with loss of consciousness of 6 hours to 24 hours, subsequent encounter |
| S06344S | ICD10 | Traumatic hemorrhage of right cerebrum with loss of consciousness of 6 hours to 24 hours, sequela |
| S06345A | ICD10 | Traumatic hemorrhage of right cerebrum with loss of consciousness greater than 24 hours with return to pre-existing conscious level, initial encounter |
| S06345D | ICD10 | Traumatic hemorrhage of right cerebrum with loss of consciousness greater than 24 hours with return to pre-existing conscious level, subsequent encounter |
| S06345S | ICD10 | Traumatic hemorrhage of right cerebrum with loss of consciousness greater than 24 hours with return to pre-existing conscious level, sequela |
| S06346A | ICD10 | Traumatic hemorrhage of right cerebrum with loss of consciousness greater than 24 hours without return to pre-existing conscious level with patient surviving, initial encounter |
| S06346D | ICD10 | Traumatic hemorrhage of right cerebrum with loss of consciousness greater than 24 hours without return to pre-existing conscious level with patient surviving, subsequent encounter |
| S06346S | ICD10 | Traumatic hemorrhage of right cerebrum with loss of consciousness greater than 24 hours without return to pre-existing conscious level with patient surviving, sequela |
| S06347A | ICD10 | Traumatic hemorrhage of right cerebrum with loss of consciousness of any duration with death due to brain injury prior to regaining consciousness, initial encounter |
| S06348A | ICD10 | Traumatic hemorrhage of right cerebrum with loss of consciousness of any duration with death due to other cause prior to regaining consciousness, initial encounter |
| S06349A | ICD10 | Traumatic hemorrhage of right cerebrum with loss of consciousness of unspecified duration, initial encounter |
| S06349D | ICD10 | Traumatic hemorrhage of right cerebrum with loss of consciousness of unspecified duration, subsequent encounter |
| S06349S | ICD10 | Traumatic hemorrhage of right cerebrum with loss of consciousness of unspecified duration, sequela |
| S06350A | ICD10 | Traumatic hemorrhage of left cerebrum without loss of consciousness, initial encounter |
| S06350D | ICD10 | Traumatic hemorrhage of left cerebrum without loss of consciousness, subsequent encounter |
| S06350S | ICD10 | Traumatic hemorrhage of left cerebrum without loss of consciousness, sequela |
| S06351A | ICD10 | Traumatic hemorrhage of left cerebrum with loss of consciousness of 30 minutes or less, initial encounter |
| S06351D | ICD10 | Traumatic hemorrhage of left cerebrum with loss of consciousness of 30 minutes or less, subsequent encounter |
| S06351S | ICD10 | Traumatic hemorrhage of left cerebrum with loss of consciousness of 30 minutes or less, sequela |
| S06352A | ICD10 | Traumatic hemorrhage of left cerebrum with loss of consciousness of 31 minutes to 59 minutes, initial encounter |
| S06352D | ICD10 | Traumatic hemorrhage of left cerebrum with loss of consciousness of 31 minutes to 59 minutes, subsequent encounter |
| S06352S | ICD10 | Traumatic hemorrhage of left cerebrum with loss of consciousness of 31 minutes to 59 minutes, sequela |
| S06353A | ICD10 | Traumatic hemorrhage of left cerebrum with loss of consciousness of 1 hours to 5 hours 59 minutes, initial encounter |
| S06353D | ICD10 | Traumatic hemorrhage of left cerebrum with loss of consciousness of 1 hours to 5 hours 59 minutes, subsequent encounter |
| S06353S | ICD10 | Traumatic hemorrhage of left cerebrum with loss of consciousness of 1 hours to 5 hours 59 minutes, sequela |
| V425 | ICD10 | Cornea replaced by transplant |
| S06354A | ICD10 | Traumatic hemorrhage of left cerebrum with loss of consciousness of 6 hours to 24 hours, initial encounter |
| S06354D | ICD10 | Traumatic hemorrhage of left cerebrum with loss of consciousness of 6 hours to 24 hours, subsequent encounter |
| S06354S | ICD10 | Traumatic hemorrhage of left cerebrum with loss of consciousness of 6 hours to 24 hours, sequela |
| S06355A | ICD10 | Traumatic hemorrhage of left cerebrum with loss of consciousness greater than 24 hours with return to pre-existing conscious level, initial encounter |
| S06355D | ICD10 | Traumatic hemorrhage of left cerebrum with loss of consciousness greater than 24 hours with return to pre-existing conscious level, subsequent encounter |
| S06355S | ICD10 | Traumatic hemorrhage of left cerebrum with loss of consciousness greater than 24 hours with return to pre-existing conscious level, sequela |
| S06356A | ICD10 | Traumatic hemorrhage of left cerebrum with loss of consciousness greater than 24 hours without return to pre-existing conscious level with patient surviving, initial encounter |
| S06356D | ICD10 | Traumatic hemorrhage of left cerebrum with loss of consciousness greater than 24 hours without return to pre-existing conscious level with patient surviving, subsequent encounter |
| S06356S | ICD10 | Traumatic hemorrhage of left cerebrum with loss of consciousness greater than 24 hours without return to pre-existing conscious level with patient surviving, sequela |
| S06357A | ICD10 | Traumatic hemorrhage of left cerebrum with loss of consciousness of any duration with death due to brain injury prior to regaining consciousness, initial encounter |
| S06358A | ICD10 | Traumatic hemorrhage of left cerebrum with loss of consciousness of any duration with death due to other cause prior to regaining consciousness, initial encounter |
| S06359A | ICD10 | Traumatic hemorrhage of left cerebrum with loss of consciousness of unspecified duration, initial encounter |
| S06359D | ICD10 | Traumatic hemorrhage of left cerebrum with loss of consciousness of unspecified duration, subsequent encounter |
| S06359S | ICD10 | Traumatic hemorrhage of left cerebrum with loss of consciousness of unspecified duration, sequela |
| S06360A | ICD10 | Traumatic hemorrhage of cerebrum, unspecified, without loss of consciousness, initial encounter |
| S06360D | ICD10 | Traumatic hemorrhage of cerebrum, unspecified, without loss of consciousness, subsequent encounter |
| S06360S | ICD10 | Traumatic hemorrhage of cerebrum, unspecified, without loss of consciousness, sequela |
| S06361A | ICD10 | Traumatic hemorrhage of cerebrum, unspecified, with loss of consciousness of 30 minutes or less, initial encounter |
| S06361D | ICD10 | Traumatic hemorrhage of cerebrum, unspecified, with loss of consciousness of 30 minutes or less, subsequent encounter |
| S06361S | ICD10 | Traumatic hemorrhage of cerebrum, unspecified, with loss of consciousness of 30 minutes or less, sequela |
| S06362A | ICD10 | Traumatic hemorrhage of cerebrum, unspecified, with loss of consciousness of 31 minutes to 59 minutes, initial encounter |
| S06362D | ICD10 | Traumatic hemorrhage of cerebrum, unspecified, with loss of consciousness of 31 minutes to 59 minutes, subsequent encounter |
| S06362S | ICD10 | Traumatic hemorrhage of cerebrum, unspecified, with loss of consciousness of 31 minutes to 59 minutes, sequela |
| S06363A | ICD10 | Traumatic hemorrhage of cerebrum, unspecified, with loss of consciousness of 1 hours to 5 hours 59 minutes, initial encounter |
| S06363D | ICD10 | Traumatic hemorrhage of cerebrum, unspecified, with loss of consciousness of 1 hours to 5 hours 59 minutes, subsequent encounter |
| S06363S | ICD10 | Traumatic hemorrhage of cerebrum, unspecified, with loss of consciousness of 1 hours to 5 hours 59 minutes, sequela |
| S06364A | ICD10 | Traumatic hemorrhage of cerebrum, unspecified, with loss of consciousness of 6 hours to 24 hours, initial encounter |
| S06364D | ICD10 | Traumatic hemorrhage of cerebrum, unspecified, with loss of consciousness of 6 hours to 24 hours, subsequent encounter |
| S06364S | ICD10 | Traumatic hemorrhage of cerebrum, unspecified, with loss of consciousness of 6 hours to 24 hours, sequela |
| S06365A | ICD10 | Traumatic hemorrhage of cerebrum, unspecified, with loss of consciousness greater than 24 hours with return to pre-existing conscious level, initial encounter |
| S06365D | ICD10 | Traumatic hemorrhage of cerebrum, unspecified, with loss of consciousness greater than 24 hours with return to pre-existing conscious level, subsequent encounter |
| S06365S | ICD10 | Traumatic hemorrhage of cerebrum, unspecified, with loss of consciousness greater than 24 hours with return to pre-existing conscious level, sequela |
| S06366A | ICD10 | Traumatic hemorrhage of cerebrum, unspecified, with loss of consciousness greater than 24 hours without return to pre-existing conscious level with patient surviving, initial encounter |
| S06366D | ICD10 | Traumatic hemorrhage of cerebrum, unspecified, with loss of consciousness greater than 24 hours without return to pre-existing conscious level with patient surviving, subsequent encounter |
| S06366S | ICD10 | Traumatic hemorrhage of cerebrum, unspecified, with loss of consciousness greater than 24 hours without return to pre-existing conscious level with patient surviving, sequela |
| S06367A | ICD10 | Traumatic hemorrhage of cerebrum, unspecified, with loss of consciousness of any duration with death due to brain injury prior to regaining consciousness, initial encounter |
| S06368A | ICD10 | Traumatic hemorrhage of cerebrum, unspecified, with loss of consciousness of any duration with death due to other cause prior to regaining consciousness, initial encounter |
| S06369A | ICD10 | Traumatic hemorrhage of cerebrum, unspecified, with loss of consciousness of unspecified duration, initial encounter |
| S06369D | ICD10 | Traumatic hemorrhage of cerebrum, unspecified, with loss of consciousness of unspecified duration, subsequent encounter |
| S06369S | ICD10 | Traumatic hemorrhage of cerebrum, unspecified, with loss of consciousness of unspecified duration, sequela |
| S06370A | ICD10 | Contusion, laceration, and hemorrhage of cerebellum without loss of consciousness, initial encounter |
| S06370D | ICD10 | Contusion, laceration, and hemorrhage of cerebellum without loss of consciousness, subsequent encounter |
| S06370S | ICD10 | Contusion, laceration, and hemorrhage of cerebellum without loss of consciousness, sequela |
| S06371A | ICD10 | Contusion, laceration, and hemorrhage of cerebellum with loss of consciousness of 30 minutes or less, initial encounter |
| S06371D | ICD10 | Contusion, laceration, and hemorrhage of cerebellum with loss of consciousness of 30 minutes or less, subsequent encounter |
| S06371S | ICD10 | Contusion, laceration, and hemorrhage of cerebellum with loss of consciousness of 30 minutes or less, sequela |
| S06372A | ICD10 | Contusion, laceration, and hemorrhage of cerebellum with loss of consciousness of 31 minutes to 59 minutes, initial encounter |
| S06372D | ICD10 | Contusion, laceration, and hemorrhage of cerebellum with loss of consciousness of 31 minutes to 59 minutes, subsequent encounter |
| S06372S | ICD10 | Contusion, laceration, and hemorrhage of cerebellum with loss of consciousness of 31 minutes to 59 minutes, sequela |
| S06373A | ICD10 | Contusion, laceration, and hemorrhage of cerebellum with loss of consciousness of 1 hour to 5 hours 59 minutes, initial encounter |
| S06373D | ICD10 | Contusion, laceration, and hemorrhage of cerebellum with loss of consciousness of 1 hour to 5 hours 59 minutes, subsequent encounter |
| S06373S | ICD10 | Contusion, laceration, and hemorrhage of cerebellum with loss of consciousness of 1 hour to 5 hours 59 minutes, sequela |
| S06374A | ICD10 | Contusion, laceration, and hemorrhage of cerebellum with loss of consciousness of 6 hours to 24 hours, initial encounter |
| S06374D | ICD10 | Contusion, laceration, and hemorrhage of cerebellum with loss of consciousness of 6 hours to 24 hours, subsequent encounter |
| S06374S | ICD10 | Contusion, laceration, and hemorrhage of cerebellum with loss of consciousness of 6 hours to 24 hours, sequela |
| S06375A | ICD10 | Contusion, laceration, and hemorrhage of cerebellum with loss of consciousness greater than 24 hours with return to pre-existing conscious level, initial encounter |
| S06375D | ICD10 | Contusion, laceration, and hemorrhage of cerebellum with loss of consciousness greater than 24 hours with return to pre-existing conscious level, subsequent encounter |
| S06375S | ICD10 | Contusion, laceration, and hemorrhage of cerebellum with loss of consciousness greater than 24 hours with return to pre-existing conscious level, sequela |
| S06376A | ICD10 | Contusion, laceration, and hemorrhage of cerebellum with loss of consciousness greater than 24 hours without return to pre-existing conscious level with patient surviving, initial encounter |
| S06376D | ICD10 | Contusion, laceration, and hemorrhage of cerebellum with loss of consciousness greater than 24 hours without return to pre-existing conscious level with patient surviving, subsequent encounter |
| S06376S | ICD10 | Contusion, laceration, and hemorrhage of cerebellum with loss of consciousness greater than 24 hours without return to pre-existing conscious level with patient surviving, sequela |
| S06377A | ICD10 | Contusion, laceration, and hemorrhage of cerebellum with loss of consciousness of any duration with death due to brain injury prior to regaining consciousness, initial encounter |
| S06378A | ICD10 | Contusion, laceration, and hemorrhage of cerebellum with loss of consciousness of any duration with death due to other cause prior to regaining consciousness, initial encounter |
| S06379A | ICD10 | Contusion, laceration, and hemorrhage of cerebellum with loss of consciousness of unspecified duration, initial encounter |
| S06379D | ICD10 | Contusion, laceration, and hemorrhage of cerebellum with loss of consciousness of unspecified duration, subsequent encounter |
| S06379S | ICD10 | Contusion, laceration, and hemorrhage of cerebellum with loss of consciousness of unspecified duration, sequela |
| S06380A | ICD10 | Contusion, laceration, and hemorrhage of brainstem without loss of consciousness, initial encounter |
| S06380D | ICD10 | Contusion, laceration, and hemorrhage of brainstem without loss of consciousness, subsequent encounter |
| S06380S | ICD10 | Contusion, laceration, and hemorrhage of brainstem without loss of consciousness, sequela |
| S06381A | ICD10 | Contusion, laceration, and hemorrhage of brainstem with loss of consciousness of 30 minutes or less, initial encounter |
| S06381D | ICD10 | Contusion, laceration, and hemorrhage of brainstem with loss of consciousness of 30 minutes or less, subsequent encounter |
| S06381S | ICD10 | Contusion, laceration, and hemorrhage of brainstem with loss of consciousness of 30 minutes or less, sequela |
| S06382A | ICD10 | Contusion, laceration, and hemorrhage of brainstem with loss of consciousness of 31 minutes to 59 minutes, initial encounter |
| S06382D | ICD10 | Contusion, laceration, and hemorrhage of brainstem with loss of consciousness of 31 minutes to 59 minutes, subsequent encounter |
| S06382S | ICD10 | Contusion, laceration, and hemorrhage of brainstem with loss of consciousness of 31 minutes to 59 minutes, sequela |
| S06383A | ICD10 | Contusion, laceration, and hemorrhage of brainstem with loss of consciousness of 1 hour to 5 hours 59 minutes, initial encounter |
| S06383D | ICD10 | Contusion, laceration, and hemorrhage of brainstem with loss of consciousness of 1 hour to 5 hours 59 minutes, subsequent encounter |
| S06383S | ICD10 | Contusion, laceration, and hemorrhage of brainstem with loss of consciousness of 1 hour to 5 hours 59 minutes, sequela |
| S06384A | ICD10 | Contusion, laceration, and hemorrhage of brainstem with loss of consciousness of 6 hours to 24 hours, initial encounter |
| S06384D | ICD10 | Contusion, laceration, and hemorrhage of brainstem with loss of consciousness of 6 hours to 24 hours, subsequent encounter |
| S06384S | ICD10 | Contusion, laceration, and hemorrhage of brainstem with loss of consciousness of 6 hours to 24 hours, sequela |
| S06385A | ICD10 | Contusion, laceration, and hemorrhage of brainstem with loss of consciousness greater than 24 hours with return to pre-existing conscious level, initial encounter |
| S06385D | ICD10 | Contusion, laceration, and hemorrhage of brainstem with loss of consciousness greater than 24 hours with return to pre-existing conscious level, subsequent encounter |
| S06385S | ICD10 | Contusion, laceration, and hemorrhage of brainstem with loss of consciousness greater than 24 hours with return to pre-existing conscious level, sequela |
| S06386A | ICD10 | Contusion, laceration, and hemorrhage of brainstem with loss of consciousness greater than 24 hours without return to pre-existing conscious level with patient surviving, initial encounter |
| S06386D | ICD10 | Contusion, laceration, and hemorrhage of brainstem with loss of consciousness greater than 24 hours without return to pre-existing conscious level with patient surviving, subsequent encounter |
| S06386S | ICD10 | Contusion, laceration, and hemorrhage of brainstem with loss of consciousness greater than 24 hours without return to pre-existing conscious level with patient surviving, sequela |
| S06387A | ICD10 | Contusion, laceration, and hemorrhage of brainstem with loss of consciousness of any duration with death due to brain injury prior to regaining consciousness, initial encounter |
| S06388A | ICD10 | Contusion, laceration, and hemorrhage of brainstem with loss of consciousness of any duration with death due to other cause prior to regaining consciousness, initial encounter |
| S06389A | ICD10 | Contusion, laceration, and hemorrhage of brainstem with loss of consciousness of unspecified duration, initial encounter |
| S06389D | ICD10 | Contusion, laceration, and hemorrhage of brainstem with loss of consciousness of unspecified duration, subsequent encounter |
| S078XXS | ICD10 | Crushing injury of other parts of head, sequela |
| S06389S | ICD10 | Contusion, laceration, and hemorrhage of brainstem with loss of consciousness of unspecified duration, sequela |
| S06335D | ICD10 | Contusion and laceration of cerebrum, unspecified, with loss of consciousness greater than 24 hours with return to pre-existing conscious level, subsequent encounter |
| S06335S | ICD10 | Contusion and laceration of cerebrum, unspecified, with loss of consciousness greater than 24 hours with return to pre-existing conscious level, sequela |
| S06355A | ICD10 | Traumatic hemorrhage of left cerebrum with loss of consciousness greater than 24 hours with return to pre-existing conscious level, initial encounter |
| S06355D | ICD10 | Traumatic hemorrhage of left cerebrum with loss of consciousness greater than 24 hours with return to pre-existing conscious level, subsequent encounter |
| S064X0A | ICD10 | Epidural hemorrhage without loss of consciousness, initial encounter |
| S064X0D | ICD10 | Epidural hemorrhage without loss of consciousness, subsequent encounter |
| S064X0S | ICD10 | Epidural hemorrhage without loss of consciousness, sequela |
| S064X1A | ICD10 | Epidural hemorrhage with loss of consciousness of 30 minutes or less, initial encounter |
| S064X1D | ICD10 | Epidural hemorrhage with loss of consciousness of 30 minutes or less, subsequent encounter |
| S064X1S | ICD10 | Epidural hemorrhage with loss of consciousness of 30 minutes or less, sequela |
| S064X2A | ICD10 | Epidural hemorrhage with loss of consciousness of 31 minutes to 59 minutes, initial encounter |
| S064X2D | ICD10 | Epidural hemorrhage with loss of consciousness of 31 minutes to 59 minutes, subsequent encounter |
| S064X2S | ICD10 | Epidural hemorrhage with loss of consciousness of 31 minutes to 59 minutes, sequela |
| S064X3A | ICD10 | Epidural hemorrhage with loss of consciousness of 1 hour to 5 hours 59 minutes, initial encounter |
| S064X3D | ICD10 | Epidural hemorrhage with loss of consciousness of 1 hour to 5 hours 59 minutes, subsequent encounter |
| S064X3S | ICD10 | Epidural hemorrhage with loss of consciousness of 1 hour to 5 hours 59 minutes, sequela |
| S064X4A | ICD10 | Epidural hemorrhage with loss of consciousness of 6 hours to 24 hours, initial encounter |
| S064X4D | ICD10 | Epidural hemorrhage with loss of consciousness of 6 hours to 24 hours, subsequent encounter |
| S064X4S | ICD10 | Epidural hemorrhage with loss of consciousness of 6 hours to 24 hours, sequela |
| S064X5A | ICD10 | Epidural hemorrhage with loss of consciousness greater than 24 hours with return to pre-existing conscious level, initial encounter |
| S064X5D | ICD10 | Epidural hemorrhage with loss of consciousness greater than 24 hours with return to pre-existing conscious level, subsequent encounter |
| S064X5S | ICD10 | Epidural hemorrhage with loss of consciousness greater than 24 hours with return to pre-existing conscious level, sequela |
| S064X6A | ICD10 | Epidural hemorrhage with loss of consciousness greater than 24 hours without return to pre-existing conscious level with patient surviving, initial encounter |
| S064X6D | ICD10 | Epidural hemorrhage with loss of consciousness greater than 24 hours without return to pre-existing conscious level with patient surviving, subsequent encounter |
| S064X6S | ICD10 | Epidural hemorrhage with loss of consciousness greater than 24 hours without return to pre-existing conscious level with patient surviving, sequela |
| S064X7A | ICD10 | Epidural hemorrhage with loss of consciousness of any duration with death due to brain injury prior to regaining consciousness, initial encounter |
| S064X8A | ICD10 | Epidural hemorrhage with loss of consciousness of any duration with death due to other causes prior to regaining consciousness, initial encounter |
| S064X9A | ICD10 | Epidural hemorrhage with loss of consciousness of unspecified duration, initial encounter |
| S064X9D | ICD10 | Epidural hemorrhage with loss of consciousness of unspecified duration, subsequent encounter |
| S064X9S | ICD10 | Epidural hemorrhage with loss of consciousness of unspecified duration, sequela |
| S065X0A | ICD10 | Traumatic subdural hemorrhage without loss of consciousness, initial encounter |
| S065X0D | ICD10 | Traumatic subdural hemorrhage without loss of consciousness, subsequent encounter |
| S065X0S | ICD10 | Traumatic subdural hemorrhage without loss of consciousness, sequela |
| S065X1A | ICD10 | Traumatic subdural hemorrhage with loss of consciousness of 30 minutes or less, initial encounter |
| S065X1D | ICD10 | Traumatic subdural hemorrhage with loss of consciousness of 30 minutes or less, subsequent encounter |
| S065X1S | ICD10 | Traumatic subdural hemorrhage with loss of consciousness of 30 minutes or less, sequela |
| S065X2A | ICD10 | Traumatic subdural hemorrhage with loss of consciousness of 31 minutes to 59 minutes, initial encounter |
| S065X2D | ICD10 | Traumatic subdural hemorrhage with loss of consciousness of 31 minutes to 59 minutes, subsequent encounter |
| S065X2S | ICD10 | Traumatic subdural hemorrhage with loss of consciousness of 31 minutes to 59 minutes, sequela |
| S065X3A | ICD10 | Traumatic subdural hemorrhage with loss of consciousness of 1 hour to 5 hours 59 minutes, initial encounter |
| S065X3D | ICD10 | Traumatic subdural hemorrhage with loss of consciousness of 1 hour to 5 hours 59 minutes, subsequent encounter |
| S065X3S | ICD10 | Traumatic subdural hemorrhage with loss of consciousness of 1 hour to 5 hours 59 minutes, sequela |
| S065X4A | ICD10 | Traumatic subdural hemorrhage with loss of consciousness of 6 hours to 24 hours, initial encounter |
| S065X4D | ICD10 | Traumatic subdural hemorrhage with loss of consciousness of 6 hours to 24 hours, subsequent encounter |
| S065X4S | ICD10 | Traumatic subdural hemorrhage with loss of consciousness of 6 hours to 24 hours, sequela |
| S065X5A | ICD10 | Traumatic subdural hemorrhage with loss of consciousness greater than 24 hours with return to pre-existing conscious level, initial encounter |
| S065X5D | ICD10 | Traumatic subdural hemorrhage with loss of consciousness greater than 24 hours with return to pre-existing conscious level, subsequent encounter |
| S065X5S | ICD10 | Traumatic subdural hemorrhage with loss of consciousness greater than 24 hours with return to pre-existing conscious level, sequela |
| S065X6A | ICD10 | Traumatic subdural hemorrhage with loss of consciousness greater than 24 hours without return to pre-existing conscious level with patient surviving, initial encounter |
| S065X6D | ICD10 | Traumatic subdural hemorrhage with loss of consciousness greater than 24 hours without return to pre-existing conscious level with patient surviving, subsequent encounter |
| S065X6S | ICD10 | Traumatic subdural hemorrhage with loss of consciousness greater than 24 hours without return to pre-existing conscious level with patient surviving, sequela |
| S065X7A | ICD10 | Traumatic subdural hemorrhage with loss of consciousness of any duration with death due to brain injury before regaining consciousness, initial encounter |
| S065X8A | ICD10 | Traumatic subdural hemorrhage with loss of consciousness of any duration with death due to other cause before regaining consciousness, initial encounter |
| S065X9A | ICD10 | Traumatic subdural hemorrhage with loss of consciousness of unspecified duration, initial encounter |
| S065X9D | ICD10 | Traumatic subdural hemorrhage with loss of consciousness of unspecified duration, subsequent encounter |
| S065X9S | ICD10 | Traumatic subdural hemorrhage with loss of consciousness of unspecified duration, sequela |
| S066X0A | ICD10 | Traumatic subarachnoid hemorrhage without loss of consciousness, initial encounter |
| S066X0D | ICD10 | Traumatic subarachnoid hemorrhage without loss of consciousness, subsequent encounter |
| S066X0S | ICD10 | Traumatic subarachnoid hemorrhage without loss of consciousness, sequela |
| S066X1A | ICD10 | Traumatic subarachnoid hemorrhage with loss of consciousness of 30 minutes or less, initial encounter |
| S066X1D | ICD10 | Traumatic subarachnoid hemorrhage with loss of consciousness of 30 minutes or less, subsequent encounter |
| S066X1S | ICD10 | Traumatic subarachnoid hemorrhage with loss of consciousness of 30 minutes or less, sequela |
| S066X2A | ICD10 | Traumatic subarachnoid hemorrhage with loss of consciousness of 31 minutes to 59 minutes, initial encounter |
| S066X2D | ICD10 | Traumatic subarachnoid hemorrhage with loss of consciousness of 31 minutes to 59 minutes, subsequent encounter |
| S066X2S | ICD10 | Traumatic subarachnoid hemorrhage with loss of consciousness of 31 minutes to 59 minutes, sequela |
| S066X3A | ICD10 | Traumatic subarachnoid hemorrhage with loss of consciousness of 1 hour to 5 hours 59 minutes, initial encounter |
| S066X3D | ICD10 | Traumatic subarachnoid hemorrhage with loss of consciousness of 1 hour to 5 hours 59 minutes, subsequent encounter |
| S066X3S | ICD10 | Traumatic subarachnoid hemorrhage with loss of consciousness of 1 hour to 5 hours 59 minutes, sequela |
| S066X4A | ICD10 | Traumatic subarachnoid hemorrhage with loss of consciousness of 6 hours to 24 hours, initial encounter |
| S066X4D | ICD10 | Traumatic subarachnoid hemorrhage with loss of consciousness of 6 hours to 24 hours, subsequent encounter |
| S066X4S | ICD10 | Traumatic subarachnoid hemorrhage with loss of consciousness of 6 hours to 24 hours, sequela |
| S066X5A | ICD10 | Traumatic subarachnoid hemorrhage with loss of consciousness greater than 24 hours with return to pre-existing conscious level, initial encounter |
| S066X5D | ICD10 | Traumatic subarachnoid hemorrhage with loss of consciousness greater than 24 hours with return to pre-existing conscious level, subsequent encounter |
| S066X5S | ICD10 | Traumatic subarachnoid hemorrhage with loss of consciousness greater than 24 hours with return to pre-existing conscious level, sequela |
| S066X6A | ICD10 | Traumatic subarachnoid hemorrhage with loss of consciousness greater than 24 hours without return to pre-existing conscious level with patient surviving, initial encounter |
| S066X6D | ICD10 | Traumatic subarachnoid hemorrhage with loss of consciousness greater than 24 hours without return to pre-existing conscious level with patient surviving, subsequent encounter |
| S066X6S | ICD10 | Traumatic subarachnoid hemorrhage with loss of consciousness greater than 24 hours without return to pre-existing conscious level with patient surviving, sequela |
| S066X7A | ICD10 | Traumatic subarachnoid hemorrhage with loss of consciousness of any duration with death due to brain injury prior to regaining consciousness, initial encounter |
| S066X8A | ICD10 | Traumatic subarachnoid hemorrhage with loss of consciousness of any duration with death due to other cause prior to regaining consciousness, initial encounter |
| S066X9A | ICD10 | Traumatic subarachnoid hemorrhage with loss of consciousness of unspecified duration, initial encounter |
| S066X9D | ICD10 | Traumatic subarachnoid hemorrhage with loss of consciousness of unspecified duration, subsequent encounter |
| S066X9S | ICD10 | Traumatic subarachnoid hemorrhage with loss of consciousness of unspecified duration, sequela |
| S06810A | ICD10 | Injury of right internal carotid artery, intracranial portion, not elsewhere classified without loss of consciousness, initial encounter |
| S06810D | ICD10 | Injury of right internal carotid artery, intracranial portion, not elsewhere classified without loss of consciousness, subsequent encounter |
| S06810S | ICD10 | Injury of right internal carotid artery, intracranial portion, not elsewhere classified without loss of consciousness, sequela |
| S06811A | ICD10 | Injury of right internal carotid artery, intracranial portion, not elsewhere classified with loss of consciousness of 30 minutes or less, initial encounter |
| S06811D | ICD10 | Injury of right internal carotid artery, intracranial portion, not elsewhere classified with loss of consciousness of 30 minutes or less, subsequent encounter |
| S06811S | ICD10 | Injury of right internal carotid artery, intracranial portion, not elsewhere classified with loss of consciousness of 30 minutes or less, sequela |
| S06812A | ICD10 | Injury of right internal carotid artery, intracranial portion, not elsewhere classified with loss of consciousness of 31 minutes to 59 minutes, initial encounter |
| S06812D | ICD10 | Injury of right internal carotid artery, intracranial portion, not elsewhere classified with loss of consciousness of 31 minutes to 59 minutes, subsequent encounter |
| S06812S | ICD10 | Injury of right internal carotid artery, intracranial portion, not elsewhere classified with loss of consciousness of 31 minutes to 59 minutes, sequela |
| S06813A | ICD10 | Injury of right internal carotid artery, intracranial portion, not elsewhere classified with loss of consciousness of 1 hour to 5 hours 59 minutes, initial encounter |
| S06813D | ICD10 | Injury of right internal carotid artery, intracranial portion, not elsewhere classified with loss of consciousness of 1 hour to 5 hours 59 minutes, subsequent encounter |
| S06813S | ICD10 | Injury of right internal carotid artery, intracranial portion, not elsewhere classified with loss of consciousness of 1 hour to 5 hours 59 minutes, sequela |
| S06814A | ICD10 | Injury of right internal carotid artery, intracranial portion, not elsewhere classified with loss of consciousness of 6 hours to 24 hours, initial encounter |
| S06814D | ICD10 | Injury of right internal carotid artery, intracranial portion, not elsewhere classified with loss of consciousness of 6 hours to 24 hours, subsequent encounter |
| S06814S | ICD10 | Injury of right internal carotid artery, intracranial portion, not elsewhere classified with loss of consciousness of 6 hours to 24 hours, sequela |
| S06815A | ICD10 | Injury of right internal carotid artery, intracranial portion, not elsewhere classified with loss of consciousness greater than 24 hours with return to pre-existing conscious level, initial encounter |
| S06815D | ICD10 | Injury of right internal carotid artery, intracranial portion, not elsewhere classified with loss of consciousness greater than 24 hours with return to pre-existing conscious level, subsequent encounter |
| S06815S | ICD10 | Injury of right internal carotid artery, intracranial portion, not elsewhere classified with loss of consciousness greater than 24 hours with return to pre-existing conscious level, sequela |
| S06816A | ICD10 | Injury of right internal carotid artery, intracranial portion, not elsewhere classified with loss of consciousness greater than 24 hours without return to pre-existing conscious level with patient surviving, initial encounter |
| S079XXA | ICD10 | Crushing injury of head, part unspecified, initial encounter |
| S06816D | ICD10 | Injury of right internal carotid artery, intracranial portion, not elsewhere classified with loss of consciousness greater than 24 hours without return to pre-existing conscious level with patient surviving, subsequent encounter |
| S06816S | ICD10 | Injury of right internal carotid artery, intracranial portion, not elsewhere classified with loss of consciousness greater than 24 hours without return to pre-existing conscious level with patient surviving, sequela |
| S06817A | ICD10 | Injury of right internal carotid artery, intracranial portion, not elsewhere classified with loss of consciousness of any duration with death due to brain injury prior to regaining consciousness, initial encounter |
| S06818A | ICD10 | Injury of right internal carotid artery, intracranial portion, not elsewhere classified with loss of consciousness of any duration with death due to other cause prior to regaining consciousness, initial encounter |
| S06819A | ICD10 | Injury of right internal carotid artery, intracranial portion, not elsewhere classified with loss of consciousness of unspecified duration, initial encounter |
| S06819D | ICD10 | Injury of right internal carotid artery, intracranial portion, not elsewhere classified with loss of consciousness of unspecified duration, subsequent encounter |
| S06819S | ICD10 | Injury of right internal carotid artery, intracranial portion, not elsewhere classified with loss of consciousness of unspecified duration, sequela |
| S06820A | ICD10 | Injury of left internal carotid artery, intracranial portion, not elsewhere classified without loss of consciousness, initial encounter |
| S06820D | ICD10 | Injury of left internal carotid artery, intracranial portion, not elsewhere classified without loss of consciousness, subsequent encounter |
| S06820S | ICD10 | Injury of left internal carotid artery, intracranial portion, not elsewhere classified without loss of consciousness, sequela |
| S06821A | ICD10 | Injury of left internal carotid artery, intracranial portion, not elsewhere classified with loss of consciousness of 30 minutes or less, initial encounter |
| S06821D | ICD10 | Injury of left internal carotid artery, intracranial portion, not elsewhere classified with loss of consciousness of 30 minutes or less, subsequent encounter |
| S06821S | ICD10 | Injury of left internal carotid artery, intracranial portion, not elsewhere classified with loss of consciousness of 30 minutes or less, sequela |
| S06822A | ICD10 | Injury of left internal carotid artery, intracranial portion, not elsewhere classified with loss of consciousness of 31 minutes to 59 minutes, initial encounter |
| S06822D | ICD10 | Injury of left internal carotid artery, intracranial portion, not elsewhere classified with loss of consciousness of 31 minutes to 59 minutes, subsequent encounter |
| S06822S | ICD10 | Injury of left internal carotid artery, intracranial portion, not elsewhere classified with loss of consciousness of 31 minutes to 59 minutes, sequela |
| S06823A | ICD10 | Injury of left internal carotid artery, intracranial portion, not elsewhere classified with loss of consciousness of 1 hour to 5 hours 59 minutes, initial encounter |
| S06823D | ICD10 | Injury of left internal carotid artery, intracranial portion, not elsewhere classified with loss of consciousness of 1 hour to 5 hours 59 minutes, subsequent encounter |
| S06823S | ICD10 | Injury of left internal carotid artery, intracranial portion, not elsewhere classified with loss of consciousness of 1 hour to 5 hours 59 minutes, sequela |
| S06824A | ICD10 | Injury of left internal carotid artery, intracranial portion, not elsewhere classified with loss of consciousness of 6 hours to 24 hours, initial encounter |
| S06824D | ICD10 | Injury of left internal carotid artery, intracranial portion, not elsewhere classified with loss of consciousness of 6 hours to 24 hours, subsequent encounter |
| S06824S | ICD10 | Injury of left internal carotid artery, intracranial portion, not elsewhere classified with loss of consciousness of 6 hours to 24 hours, sequela |
| S06825A | ICD10 | Injury of left internal carotid artery, intracranial portion, not elsewhere classified with loss of consciousness greater than 24 hours with return to pre-existing conscious level, initial encounter |
| S06825D | ICD10 | Injury of left internal carotid artery, intracranial portion, not elsewhere classified with loss of consciousness greater than 24 hours with return to pre-existing conscious level, subsequent encounter |
| S06825S | ICD10 | Injury of left internal carotid artery, intracranial portion, not elsewhere classified with loss of consciousness greater than 24 hours with return to pre-existing conscious level, sequela |
| S06826A | ICD10 | Injury of left internal carotid artery, intracranial portion, not elsewhere classified with loss of consciousness greater than 24 hours without return to pre-existing conscious level with patient surviving, initial encounter |
| S06826D | ICD10 | Injury of left internal carotid artery, intracranial portion, not elsewhere classified with loss of consciousness greater than 24 hours without return to pre-existing conscious level with patient surviving, subsequent encounter |
| S06826S | ICD10 | Injury of left internal carotid artery, intracranial portion, not elsewhere classified with loss of consciousness greater than 24 hours without return to pre-existing conscious level with patient surviving, sequela |
| S06827A | ICD10 | Injury of left internal carotid artery, intracranial portion, not elsewhere classified with loss of consciousness of any duration with death due to brain injury prior to regaining consciousness, initial encounter |
| S06828A | ICD10 | Injury of left internal carotid artery, intracranial portion, not elsewhere classified with loss of consciousness of any duration with death due to other cause prior to regaining consciousness, initial encounter |
| S06829A | ICD10 | Injury of left internal carotid artery, intracranial portion, not elsewhere classified with loss of consciousness of unspecified duration, initial encounter |
| S06829D | ICD10 | Injury of left internal carotid artery, intracranial portion, not elsewhere classified with loss of consciousness of unspecified duration, subsequent encounter |
| S06829S | ICD10 | Injury of left internal carotid artery, intracranial portion, not elsewhere classified with loss of consciousness of unspecified duration, sequela |
| S06890A | ICD10 | Other specified intracranial injury without loss of consciousness, initial encounter |
| S06890D | ICD10 | Other specified intracranial injury without loss of consciousness, subsequent encounter |
| S06890S | ICD10 | Other specified intracranial injury without loss of consciousness, sequela |
| S06891A | ICD10 | Other specified intracranial injury with loss of consciousness of 30 minutes or less, initial encounter |
| S06891D | ICD10 | Other specified intracranial injury with loss of consciousness of 30 minutes or less, subsequent encounter |
| S06891S | ICD10 | Other specified intracranial injury with loss of consciousness of 30 minutes or less, sequela |
| W880XXS | ICD10 | Exposure to X-rays, sequela |
| S06892A | ICD10 | Other specified intracranial injury with loss of consciousness of 31 minutes to 59 minutes, initial encounter |
| S06892D | ICD10 | Other specified intracranial injury with loss of consciousness of 31 minutes to 59 minutes, subsequent encounter |
| S06892S | ICD10 | Other specified intracranial injury with loss of consciousness of 31 minutes to 59 minutes, sequela |
| S06893A | ICD10 | Other specified intracranial injury with loss of consciousness of 1 hour to 5 hours 59 minutes, initial encounter |
| S06893D | ICD10 | Other specified intracranial injury with loss of consciousness of 1 hour to 5 hours 59 minutes, subsequent encounter |
| S06893S | ICD10 | Other specified intracranial injury with loss of consciousness of 1 hour to 5 hours 59 minutes, sequela |
| S06894A | ICD10 | Other specified intracranial injury with loss of consciousness of 6 hours to 24 hours, initial encounter |
| S06894D | ICD10 | Other specified intracranial injury with loss of consciousness of 6 hours to 24 hours, subsequent encounter |
| S06894S | ICD10 | Other specified intracranial injury with loss of consciousness of 6 hours to 24 hours, sequela |
| S06895A | ICD10 | Other specified intracranial injury with loss of consciousness greater than 24 hours with return to pre-existing conscious level, initial encounter |
| S06895D | ICD10 | Other specified intracranial injury with loss of consciousness greater than 24 hours with return to pre-existing conscious level, subsequent encounter |
| S06895S | ICD10 | Other specified intracranial injury with loss of consciousness greater than 24 hours with return to pre-existing conscious level, sequela |
| S06896A | ICD10 | Other specified intracranial injury with loss of consciousness greater than 24 hours without return to pre-existing conscious level with patient surviving, initial encounter |
| S06896D | ICD10 | Other specified intracranial injury with loss of consciousness greater than 24 hours without return to pre-existing conscious level with patient surviving, subsequent encounter |
| S06896S | ICD10 | Other specified intracranial injury with loss of consciousness greater than 24 hours without return to pre-existing conscious level with patient surviving, sequela |
| S06897A | ICD10 | Other specified intracranial injury with loss of consciousness of any duration with death due to brain injury prior to regaining consciousness, initial encounter |
| S06898A | ICD10 | Other specified intracranial injury with loss of consciousness of any duration with death due to other cause prior to regaining consciousness, initial encounter |
| S06899A | ICD10 | Other specified intracranial injury with loss of consciousness of unspecified duration, initial encounter |
| S06899D | ICD10 | Other specified intracranial injury with loss of consciousness of unspecified duration, subsequent encounter |
| S06899S | ICD10 | Other specified intracranial injury with loss of consciousness of unspecified duration, sequela |
| S069X0A | ICD10 | Unspecified intracranial injury without loss of consciousness, initial encounter |
| S069X0D | ICD10 | Unspecified intracranial injury without loss of consciousness, subsequent encounter |
| S069X0S | ICD10 | Unspecified intracranial injury without loss of consciousness, sequela |
| S069X1A | ICD10 | Unspecified intracranial injury with loss of consciousness of 30 minutes or less, initial encounter |
| S069X1D | ICD10 | Unspecified intracranial injury with loss of consciousness of 30 minutes or less, subsequent encounter |
| S069X1S | ICD10 | Unspecified intracranial injury with loss of consciousness of 30 minutes or less, sequela |
| S069X2A | ICD10 | Unspecified intracranial injury with loss of consciousness of 31 minutes to 59 minutes, initial encounter |
| S069X2D | ICD10 | Unspecified intracranial injury with loss of consciousness of 31 minutes to 59 minutes, subsequent encounter |
| S069X2S | ICD10 | Unspecified intracranial injury with loss of consciousness of 31 minutes to 59 minutes, sequela |
| S069X3A | ICD10 | Unspecified intracranial injury with loss of consciousness of 1 hour to 5 hours 59 minutes, initial encounter |
| S069X3D | ICD10 | Unspecified intracranial injury with loss of consciousness of 1 hour to 5 hours 59 minutes, subsequent encounter |
| S069X3S | ICD10 | Unspecified intracranial injury with loss of consciousness of 1 hour to 5 hours 59 minutes, sequela |
| S069X4A | ICD10 | Unspecified intracranial injury with loss of consciousness of 6 hours to 24 hours, initial encounter |
| S069X4D | ICD10 | Unspecified intracranial injury with loss of consciousness of 6 hours to 24 hours, subsequent encounter |
| S069X4S | ICD10 | Unspecified intracranial injury with loss of consciousness of 6 hours to 24 hours, sequela |
| S069X5A | ICD10 | Unspecified intracranial injury with loss of consciousness greater than 24 hours with return to pre-existing conscious level, initial encounter |
| S069X5D | ICD10 | Unspecified intracranial injury with loss of consciousness greater than 24 hours with return to pre-existing conscious level, subsequent encounter |
| S069X5S | ICD10 | Unspecified intracranial injury with loss of consciousness greater than 24 hours with return to pre-existing conscious level, sequela |
| S069X6A | ICD10 | Unspecified intracranial injury with loss of consciousness greater than 24 hours without return to pre-existing conscious level with patient surviving, initial encounter |
| S069X6D | ICD10 | Unspecified intracranial injury with loss of consciousness greater than 24 hours without return to pre-existing conscious level with patient surviving, subsequent encounter |
| S069X6S | ICD10 | Unspecified intracranial injury with loss of consciousness greater than 24 hours without return to pre-existing conscious level with patient surviving, sequela |
| S069X7A | ICD10 | Unspecified intracranial injury with loss of consciousness of any duration with death due to brain injury prior to regaining consciousness, initial encounter |
| S069X8A | ICD10 | Unspecified intracranial injury with loss of consciousness of any duration with death due to other cause prior to regaining consciousness, initial encounter |
| S069X9A | ICD10 | Unspecified intracranial injury with loss of consciousness of unspecified duration, initial encounter |
| S069X9D | ICD10 | Unspecified intracranial injury with loss of consciousness of unspecified duration, subsequent encounter |
| S069X9S | ICD10 | Unspecified intracranial injury with loss of consciousness of unspecified duration, sequela |
