# Supplementary material for: Propofol provides a significant survival advantage in sepsis-associated encephalopathy: A retrospective cohort study investigating one-year all-cause mortality
Source: PLoS One. 2026 Feb 5;21(2):e0340371. doi: 10.1371/journal.pone.0340371 (PMC12875438; doi:10.1371/journal.pone.0340371)
Supplement: S2 Table — (DOCX) [file pone.0340371.s002.docx]

Supporting Information

# S2 Table. Exclude patients with intracerebral hemorrhage, cerebral embolism and ischemic stroke disease from the MIMIC-IV database according to ICD-codes

| ICD-code | ICD | Description |
| --- | --- | --- |
| 430 | ICD9 | Subarachnoid hemorrhage |
| 431 | ICD9 | Intracerebral hemorrhage |
| 5430 | ICD9 | Subarachnoid hemorrhage |
| 4329 | ICD9 | Unspecified intracranial hemorrhage |
| 4321 | ICD9 | Subdural hemorrhage |
| 4320 | ICD9 | Nontraumatic extradural hemorrhage |
| 7670 | ICD9 | Subdural and cerebral hemorrhage |
| 43411 | ICD9 | Cerebral embolism with cerebral infarction |
| 4376 | ICD9 | Nonpyogenic thrombosis of intracranial venous sinus |
| V1254 | ICD9 | Personal history of transient ischemic attack (TIA), and cerebral infarction without residual deficits |
| I6000 | ICD10 | Nontraumatic subarachnoid hemorrhage from unspecified carotid siphon and bifurcation |
| I6001 | ICD10 | Nontraumatic subarachnoid hemorrhage from right carotid siphon and bifurcation |
| I6002 | ICD10 | Nontraumatic subarachnoid hemorrhage from left carotid siphon and bifurcation |
| I6010 | ICD10 | Nontraumatic subarachnoid hemorrhage from unspecified middle cerebral artery |
| I6011 | ICD10 | Nontraumatic subarachnoid hemorrhage from right middle cerebral artery |
| I6012 | ICD10 | Nontraumatic subarachnoid hemorrhage from left middle cerebral artery |
| I602 | ICD10 | Nontraumatic subarachnoid hemorrhage from anterior communicating artery |
| I6030 | ICD10 | Nontraumatic subarachnoid hemorrhage from unspecified posterior communicating artery |
| I6031 | ICD10 | Nontraumatic subarachnoid hemorrhage from right posterior communicating artery |
| I6032 | ICD10 | Nontraumatic subarachnoid hemorrhage from left posterior communicating artery |
| I604 | ICD10 | Nontraumatic subarachnoid hemorrhage from basilar artery |
| I6050 | ICD10 | Nontraumatic subarachnoid hemorrhage from unspecified vertebral artery |
| I6051 | ICD10 | Nontraumatic subarachnoid hemorrhage from right vertebral artery |
| I6052 | ICD10 | Nontraumatic subarachnoid hemorrhage from left vertebral artery |
| I606 | ICD10 | Nontraumatic subarachnoid hemorrhage from other intracranial arteries |
| I607 | ICD10 | Nontraumatic subarachnoid hemorrhage from unspecified intracranial artery |
| I608 | ICD10 | Other nontraumatic subarachnoid hemorrhage |
| I609 | ICD10 | Nontraumatic subarachnoid hemorrhage, unspecified |
| I610 | ICD10 | Nontraumatic intracerebral hemorrhage in hemisphere, subcortical |
| I611 | ICD10 | Nontraumatic intracerebral hemorrhage in hemisphere, cortical |
| I612 | ICD10 | Nontraumatic intracerebral hemorrhage in hemisphere, unspecified |
| I613 | ICD10 | Nontraumatic intracerebral hemorrhage in brain stem |
| I614 | ICD10 | Nontraumatic intracerebral hemorrhage in cerebellum |
| I615 | ICD10 | Nontraumatic intracerebral hemorrhage, intraventricular |
| I616 | ICD10 | Nontraumatic intracerebral hemorrhage, multiple localized |
| I618 | ICD10 | Other nontraumatic intracerebral hemorrhage |
| I619 | ICD10 | Nontraumatic intracerebral hemorrhage, unspecified |
| I6200 | ICD10 | Nontraumatic subdural hemorrhage, unspecified |
| I6201 | ICD10 | Nontraumatic acute subdural hemorrhage |
| I6202 | ICD10 | Nontraumatic subacute subdural hemorrhage |
| I6203 | ICD10 | Nontraumatic chronic subdural hemorrhage |
| I621 | ICD10 | Nontraumatic extradural hemorrhage |
| I629 | ICD10 | Nontraumatic intracranial hemorrhage, unspecified |
| I6300 | ICD10 | Cerebral infarction due to thrombosis of unspecified precerebral artery |
| I63011 | ICD10 | Cerebral infarction due to thrombosis of right vertebral artery |
| I63012 | ICD10 | Cerebral infarction due to thrombosis of left vertebral artery |
| I63013 | ICD10 | Cerebral infarction due to thrombosis of bilateral vertebral arteries |
| I63019 | ICD10 | Cerebral infarction due to thrombosis of unspecified vertebral artery |
| I6302 | ICD10 | Cerebral infarction due to thrombosis of basilar artery |
| I63031 | ICD10 | Cerebral infarction due to thrombosis of right carotid artery |
| I63032 | ICD10 | Cerebral infarction due to thrombosis of left carotid artery |
| I63033 | ICD10 | Cerebral infarction due to thrombosis of bilateral carotid arteries |
| I63039 | ICD10 | Cerebral infarction due to thrombosis of unspecified carotid artery |
| I6309 | ICD10 | Cerebral infarction due to thrombosis of other precerebral artery |
| I6310 | ICD10 | Cerebral infarction due to embolism of unspecified precerebral artery |
| I63111 | ICD10 | Cerebral infarction due to embolism of right vertebral artery |
| I63112 | ICD10 | Cerebral infarction due to embolism of left vertebral artery |
| I63113 | ICD10 | Cerebral infarction due to embolism of bilateral vertebral arteries |
| I63119 | ICD10 | Cerebral infarction due to embolism of unspecified vertebral artery |
| I6312 | ICD10 | Cerebral infarction due to embolism of basilar artery |
| I63131 | ICD10 | Cerebral infarction due to embolism of right carotid artery |
| I63132 | ICD10 | Cerebral infarction due to embolism of left carotid artery |
| I63133 | ICD10 | Cerebral infarction due to embolism of bilateral carotid arteries |
| I63139 | ICD10 | Cerebral infarction due to embolism of unspecified carotid artery |
| I6319 | ICD10 | Cerebral infarction due to embolism of other precerebral artery |
| I6320 | ICD10 | Cerebral infarction due to unspecified occlusion or stenosis of unspecified precerebral arteries |
| I63211 | ICD10 | Cerebral infarction due to unspecified occlusion or stenosis of right vertebral artery |
| I63212 | ICD10 | Cerebral infarction due to unspecified occlusion or stenosis of left vertebral artery |
| I63213 | ICD10 | Cerebral infarction due to unspecified occlusion or stenosis of bilateral vertebral arteries |
| I63219 | ICD10 | Cerebral infarction due to unspecified occlusion or stenosis of unspecified vertebral artery |
| I6322 | ICD10 | Cerebral infarction due to unspecified occlusion or stenosis of basilar artery |
| I63231 | ICD10 | Cerebral infarction due to unspecified occlusion or stenosis of right carotid arteries |
| I63232 | ICD10 | Cerebral infarction due to unspecified occlusion or stenosis of left carotid arteries |
| I63233 | ICD10 | Cerebral infarction due to unspecified occlusion or stenosis of bilateral carotid arteries |
| I63239 | ICD10 | Cerebral infarction due to unspecified occlusion or stenosis of unspecified carotid artery |
| I6329 | ICD10 | Cerebral infarction due to unspecified occlusion or stenosis of other precerebral arteries |
| I6330 | ICD10 | Cerebral infarction due to thrombosis of unspecified cerebral artery |
| I63311 | ICD10 | Cerebral infarction due to thrombosis of right middle cerebral artery |
| I63312 | ICD10 | Cerebral infarction due to thrombosis of left middle cerebral artery |
| I63313 | ICD10 | Cerebral infarction due to thrombosis of bilateral middle cerebral arteries |
| I63319 | ICD10 | Cerebral infarction due to thrombosis of unspecified middle cerebral artery |
| I63321 | ICD10 | Cerebral infarction due to thrombosis of right anterior cerebral artery |
| I63322 | ICD10 | Cerebral infarction due to thrombosis of left anterior cerebral artery |
| I63323 | ICD10 | Cerebral infarction due to thrombosis of bilateral anterior cerebral arteries |
| I63329 | ICD10 | Cerebral infarction due to thrombosis of unspecified anterior cerebral artery |
| I63331 | ICD10 | Cerebral infarction due to thrombosis of right posterior cerebral artery |
| I63332 | ICD10 | Cerebral infarction due to thrombosis of left posterior cerebral artery |
| I63333 | ICD10 | Cerebral infarction due to thrombosis of bilateral posterior cerebral arteries |
| I63339 | ICD10 | Cerebral infarction due to thrombosis of unspecified posterior cerebral artery |
| I63341 | ICD10 | Cerebral infarction due to thrombosis of right cerebellar artery |
| I63342 | ICD10 | Cerebral infarction due to thrombosis of left cerebellar artery |
| I63343 | ICD10 | Cerebral infarction due to thrombosis of bilateral cerebellar arteries |
| I63349 | ICD10 | Cerebral infarction due to thrombosis of unspecified cerebellar artery |
| I6339 | ICD10 | Cerebral infarction due to thrombosis of other cerebral artery |
| I6340 | ICD10 | Cerebral infarction due to embolism of unspecified cerebral artery |
| I63411 | ICD10 | Cerebral infarction due to embolism of right middle cerebral artery |
| I63412 | ICD10 | Cerebral infarction due to embolism of left middle cerebral artery |
| I63413 | ICD10 | Cerebral infarction due to embolism of bilateral middle cerebral arteries |
| I63419 | ICD10 | Cerebral infarction due to embolism of unspecified middle cerebral artery |
| I63421 | ICD10 | Cerebral infarction due to embolism of right anterior cerebral artery |
| I63422 | ICD10 | Cerebral infarction due to embolism of left anterior cerebral artery |
| I63423 | ICD10 | Cerebral infarction due to embolism of bilateral anterior cerebral arteries |
| I63429 | ICD10 | Cerebral infarction due to embolism of unspecified anterior cerebral artery |
| I63431 | ICD10 | Cerebral infarction due to embolism of right posterior cerebral artery |
| I63432 | ICD10 | Cerebral infarction due to embolism of left posterior cerebral artery |
| I63433 | ICD10 | Cerebral infarction due to embolism of bilateral posterior cerebral arteries |
| I63439 | ICD10 | Cerebral infarction due to embolism of unspecified posterior cerebral artery |
| I63441 | ICD10 | Cerebral infarction due to embolism of right cerebellar artery |
| I63442 | ICD10 | Cerebral infarction due to embolism of left cerebellar artery |
| I63443 | ICD10 | Cerebral infarction due to embolism of bilateral cerebellar arteries |
| I63449 | ICD10 | Cerebral infarction due to embolism of unspecified cerebellar artery |
| I6349 | ICD10 | Cerebral infarction due to embolism of other cerebral artery |
| I6350 | ICD10 | Cerebral infarction due to unspecified occlusion or stenosis of unspecified cerebral artery |
| I63511 | ICD10 | Cerebral infarction due to unspecified occlusion or stenosis of right middle cerebral artery |
| I63512 | ICD10 | Cerebral infarction due to unspecified occlusion or stenosis of left middle cerebral artery |
| I63513 | ICD10 | Cerebral infarction due to unspecified occlusion or stenosis of bilateral middle cerebral arteries |
| I63519 | ICD10 | Cerebral infarction due to unspecified occlusion or stenosis of unspecified middle cerebral artery |
| I63521 | ICD10 | Cerebral infarction due to unspecified occlusion or stenosis of right anterior cerebral artery |
| I63522 | ICD10 | Cerebral infarction due to unspecified occlusion or stenosis of left anterior cerebral artery |
| I63523 | ICD10 | Cerebral infarction due to unspecified occlusion or stenosis of bilateral anterior cerebral arteries |
| I63529 | ICD10 | Cerebral infarction due to unspecified occlusion or stenosis of unspecified anterior cerebral artery |
| I63531 | ICD10 | Cerebral infarction due to unspecified occlusion or stenosis of right posterior cerebral artery |
| I63532 | ICD10 | Cerebral infarction due to unspecified occlusion or stenosis of left posterior cerebral artery |
| I63533 | ICD10 | Cerebral infarction due to unspecified occlusion or stenosis of bilateral posterior cerebral arteries |
| I63539 | ICD10 | Cerebral infarction due to unspecified occlusion or stenosis of unspecified posterior cerebral artery |
| I63541 | ICD10 | Cerebral infarction due to unspecified occlusion or stenosis of right cerebellar artery |
| I63542 | ICD10 | Cerebral infarction due to unspecified occlusion or stenosis of left cerebellar artery |
| I63543 | ICD10 | Cerebral infarction due to unspecified occlusion or stenosis of bilateral cerebellar arteries |
| I63549 | ICD10 | Cerebral infarction due to unspecified occlusion or stenosis of unspecified cerebellar artery |
| I6359 | ICD10 | Cerebral infarction due to unspecified occlusion or stenosis of other cerebral artery |
| I636 | ICD10 | Cerebral infarction due to cerebral venous thrombosis, nonpyogenic |
| I6381 | ICD10 | Other cerebral infarction due to occlusion or stenosis of small artery |
| I6389 | ICD10 | Other cerebral infarction |
| I639 | ICD10 | Cerebral infarction, unspecified |
| I6501 | ICD10 | Occlusion and stenosis of right vertebral artery |
| I6502 | ICD10 | Occlusion and stenosis of left vertebral artery |
| I6503 | ICD10 | Occlusion and stenosis of bilateral vertebral arteries |
| I6509 | ICD10 | Occlusion and stenosis of unspecified vertebral artery |
| I651 | ICD10 | Occlusion and stenosis of basilar artery |
| I6521 | ICD10 | Occlusion and stenosis of right carotid artery |
| I6522 | ICD10 | Occlusion and stenosis of left carotid artery |
| I6523 | ICD10 | Occlusion and stenosis of bilateral carotid arteries |
| I6529 | ICD10 | Occlusion and stenosis of unspecified carotid artery |
| I658 | ICD10 | Occlusion and stenosis of other precerebral arteries |
| I659 | ICD10 | Occlusion and stenosis of unspecified precerebral artery |
| I6601 | ICD10 | Occlusion and stenosis of right middle cerebral artery |
| I6602 | ICD10 | Occlusion and stenosis of left middle cerebral artery |
| I6603 | ICD10 | Occlusion and stenosis of bilateral middle cerebral arteries |
| I6609 | ICD10 | Occlusion and stenosis of unspecified middle cerebral artery |
| I6611 | ICD10 | Occlusion and stenosis of right anterior cerebral artery |
| I6612 | ICD10 | Occlusion and stenosis of left anterior cerebral artery |
| I6613 | ICD10 | Occlusion and stenosis of bilateral anterior cerebral arteries |
| I6619 | ICD10 | Occlusion and stenosis of unspecified anterior cerebral artery |
| I6621 | ICD10 | Occlusion and stenosis of right posterior cerebral artery |
| I6622 | ICD10 | Occlusion and stenosis of left posterior cerebral artery |
| I6623 | ICD10 | Occlusion and stenosis of bilateral posterior cerebral arteries |
| I6629 | ICD10 | Occlusion and stenosis of unspecified posterior cerebral artery |
| I663 | ICD10 | Occlusion and stenosis of cerebellar arteries |
| I668 | ICD10 | Occlusion and stenosis of other cerebral arteries |
| I669 | ICD10 | Occlusion and stenosis of unspecified cerebral artery |
| I670 | ICD10 | Dissection of cerebral arteries, nonruptured |
| I676 | ICD10 | Nonpyogenic thrombosis of intracranial venous system |
| I677 | ICD10 | Cerebral arteritis, not elsewhere classified |
| I6781 | ICD10 | Acute cerebrovascular insufficiency |
| I6782 | ICD10 | Cerebral ischemia |
| I6783 | ICD10 | Posterior reversible encephalopathy syndrome |
| I67841 | ICD10 | Reversible cerebrovascular vasoconstriction syndrome |
| I67848 | ICD10 | Other cerebrovascular vasospasm and vasoconstriction |
| I67850 | ICD10 | Cerebral autosomal dominant arteriopathy with subcortical infarcts and leukoencephalopathy |
| I67858 | ICD10 | Other hereditary cerebrovascular disease |
| I6789 | ICD10 | Other cerebrovascular disease |
| I679 | ICD10 | Cerebrovascular disease, unspecified |
| I680 | ICD10 | Cerebral amyloid angiopathy |
| I682 | ICD10 | Cerebral arteritis in other diseases classified elsewhere |
| I688 | ICD10 | Other cerebrovascular disorders in diseases classified elsewhere |
| I6900 | ICD10 | Unspecified sequelae of nontraumatic subarachnoid hemorrhage |
| I69010 | ICD10 | Attention and concentration deficit following nontraumatic subarachnoid hemorrhage |
| I69011 | ICD10 | Memory deficit following nontraumatic subarachnoid hemorrhage |
| I69012 | ICD10 | Visuospatial deficit and spatial neglect following nontraumatic subarachnoid hemorrhage |
| I69013 | ICD10 | Psychomotor deficit following nontraumatic subarachnoid hemorrhage |
| I69014 | ICD10 | Frontal lobe and executive function deficit following nontraumatic subarachnoid hemorrhage |
| I69015 | ICD10 | Cognitive social or emotional deficit following nontraumatic subarachnoid hemorrhage |
| I69018 | ICD10 | Other symptoms and signs involving cognitive functions following nontraumatic subarachnoid hemorrhage |
| I69019 | ICD10 | Unspecified symptoms and signs involving cognitive functions following nontraumatic subarachnoid hemorrhage |
| I69020 | ICD10 | Aphasia following nontraumatic subarachnoid hemorrhage |
| I69021 | ICD10 | Dysphasia following nontraumatic subarachnoid hemorrhage |
| I69022 | ICD10 | Dysarthria following nontraumatic subarachnoid hemorrhage |
| I69023 | ICD10 | Fluency disorder following nontraumatic subarachnoid hemorrhage |
| I69028 | ICD10 | Other speech and language deficits following nontraumatic subarachnoid hemorrhage |
| I69031 | ICD10 | Monoplegia of upper limb following nontraumatic subarachnoid hemorrhage affecting right dominant side |
| I69032 | ICD10 | Monoplegia of upper limb following nontraumatic subarachnoid hemorrhage affecting left dominant side |
| I69033 | ICD10 | Monoplegia of upper limb following nontraumatic subarachnoid hemorrhage affecting right non-dominant side |
| I69034 | ICD10 | Monoplegia of upper limb following nontraumatic subarachnoid hemorrhage affecting left non-dominant side |
| I69039 | ICD10 | Monoplegia of upper limb following nontraumatic subarachnoid hemorrhage affecting unspecified side |
| I69041 | ICD10 | Monoplegia of lower limb following nontraumatic subarachnoid hemorrhage affecting right dominant side |
| I69042 | ICD10 | Monoplegia of lower limb following nontraumatic subarachnoid hemorrhage affecting left dominant side |
| I69043 | ICD10 | Monoplegia of lower limb following nontraumatic subarachnoid hemorrhage affecting right non-dominant side |
| I69044 | ICD10 | Monoplegia of lower limb following nontraumatic subarachnoid hemorrhage affecting left non-dominant side |
| I69049 | ICD10 | Monoplegia of lower limb following nontraumatic subarachnoid hemorrhage affecting unspecified side |
| I69051 | ICD10 | Hemiplegia and hemiparesis following nontraumatic subarachnoid hemorrhage affecting right dominant side |
| I69052 | ICD10 | Hemiplegia and hemiparesis following nontraumatic subarachnoid hemorrhage affecting left dominant side |
| I69053 | ICD10 | Hemiplegia and hemiparesis following nontraumatic subarachnoid hemorrhage affecting right non-dominant side |
| I69054 | ICD10 | Hemiplegia and hemiparesis following nontraumatic subarachnoid hemorrhage affecting left non-dominant side |
| I69059 | ICD10 | Hemiplegia and hemiparesis following nontraumatic subarachnoid hemorrhage affecting unspecified side |
| I69061 | ICD10 | Other paralytic syndrome following nontraumatic subarachnoid hemorrhage affecting right dominant side |
| I69062 | ICD10 | Other paralytic syndrome following nontraumatic subarachnoid hemorrhage affecting left dominant side |
| I69063 | ICD10 | Other paralytic syndrome following nontraumatic subarachnoid hemorrhage affecting right non-dominant side |
| I69064 | ICD10 | Other paralytic syndrome following nontraumatic subarachnoid hemorrhage affecting left non-dominant side |
| I69065 | ICD10 | Other paralytic syndrome following nontraumatic subarachnoid hemorrhage, bilateral |
| I69069 | ICD10 | Other paralytic syndrome following nontraumatic subarachnoid hemorrhage affecting unspecified side |
| I69090 | ICD10 | Apraxia following nontraumatic subarachnoid hemorrhage |
| I69091 | ICD10 | Dysphagia following nontraumatic subarachnoid hemorrhage |
| I69092 | ICD10 | Facial weakness following nontraumatic subarachnoid hemorrhage |
| I69093 | ICD10 | Ataxia following nontraumatic subarachnoid hemorrhage |
| I69098 | ICD10 | Other sequelae following nontraumatic subarachnoid hemorrhage |
| I6910 | ICD10 | Unspecified sequelae of nontraumatic intracerebral hemorrhage |
| I69110 | ICD10 | Attention and concentration deficit following nontraumatic intracerebral hemorrhage |
| I69111 | ICD10 | Memory deficit following nontraumatic intracerebral hemorrhage |
| I69112 | ICD10 | Visuospatial deficit and spatial neglect following nontraumatic intracerebral hemorrhage |
| I69113 | ICD10 | Psychomotor deficit following nontraumatic intracerebral hemorrhage |
| I69114 | ICD10 | Frontal lobe and executive function deficit following nontraumatic intracerebral hemorrhage |
| I69115 | ICD10 | Cognitive social or emotional deficit following nontraumatic intracerebral hemorrhage |
| I69118 | ICD10 | Other symptoms and signs involving cognitive functions following nontraumatic intracerebral hemorrhage |
| I69119 | ICD10 | Unspecified symptoms and signs involving cognitive functions following nontraumatic intracerebral hemorrhage |
| I69120 | ICD10 | Aphasia following nontraumatic intracerebral hemorrhage |
| I69121 | ICD10 | Dysphasia following nontraumatic intracerebral hemorrhage |
| I69122 | ICD10 | Dysarthria following nontraumatic intracerebral hemorrhage |
| I69123 | ICD10 | Fluency disorder following nontraumatic intracerebral hemorrhage |
| I69128 | ICD10 | Other speech and language deficits following nontraumatic intracerebral hemorrhage |
| I69311 | ICD10 | Memory deficit following cerebral infarction |
| I69312 | ICD10 | Visuospatial deficit and spatial neglect following cerebral infarction |
| I69313 | ICD10 | Psychomotor deficit following cerebral infarction |
| I69314 | ICD10 | Frontal lobe and executive function deficit following cerebral infarction |
| I69315 | ICD10 | Cognitive social or emotional deficit following cerebral infarction |
| I69318 | ICD10 | Other symptoms and signs involving cognitive functions following cerebral infarction |
| I69319 | ICD10 | Unspecified symptoms and signs involving cognitive functions following cerebral infarction |
