# Supplementary material for: Propofol provides a significant survival advantage in sepsis-associated encephalopathy: A retrospective cohort study investigating one-year all-cause mortality
Source: PLoS One. 2026 Feb 5;21(2):e0340371. doi: 10.1371/journal.pone.0340371 (PMC12875438; doi:10.1371/journal.pone.0340371)
Supplement: S3 Table — (DOCX) [file pone.0340371.s003.docx]

Supporting Information

# **S3 Table.** Exclude patients with meningitis and encephalitis disease from the MIMIC-IV database according to ICD-codes.

| ICD-code | ICD | Description |
| --- | --- | --- |
| 1300 | ICD9 | Tuberculous meningitis, unspecified |
| 1301 | ICD9 | Tuberculous meningitis, bacteriological or histological examination not done |
| 468 | ICD9 | Other specified slow virus infection of central nervous system |
| 469 | ICD9 | Unspecified slow virus infection of central nervous system |
| 470 | ICD9 | Meningitis due to coxsackie virus |
| 471 | ICD9 | Meningitis due to echo virus |
| 478 | ICD9 | Other specified viral meningitis |
| 479 | ICD9 | Unspecified viral meningitis |
| 491 | ICD9 | Meningitis due to adenovirus |
| 498 | ICD9 | Other specified non-arthropod-borne viral diseases of central nervous system |
| 499 | ICD9 | Unspecified non-arthropod-borne viral diseases of central nervous system |
| 520 | ICD9 | Postvaricella encephalitis |
| 530 | ICD9 | Herpes zoster with meningitis |
| 5319 | ICD9 | Herpes zoster with other nervous system complications |
| 5379 | ICD9 | Herpes zoster with other specified complications |
| 5472 | ICD9 | Herpes simplex meningitis |
| 550 | ICD9 | Postmeasles encephalitis |
| 360 | ICD9 | Meningococcal meningitis |
| 361 | ICD9 | Meningococcal encephalitis |
| 362 | ICD9 | Meningococcemia |
| 363 | ICD9 | Waterhouse-Friderichsen syndrome, meningococcal |
| 1302 | ICD9 | Tuberculous meningitis, bacteriological or histological examination unknown (at present) |
| 1303 | ICD9 | Tuberculous meningitis, tubercle bacilli found (in sputum) by microscopy |
| 1304 | ICD9 | Tuberculous meningitis, tubercle bacilli not found (in sputum) by microscopy, but found by bacterial culture |
| 1305 | ICD9 | Tuberculous meningitis, tubercle bacilli not found by bacteriological examination, but tuberculosis confirmed histologically |
| 1306 | ICD9 | Tuberculous meningitis, tubercle bacilli not found by bacteriological or histological examination, but tuberculosis confirmed by other methods [inoculation of animals] |
| 1310 | ICD9 | Tuberculoma of meninges, unspecified |
| 1311 | ICD9 | Tuberculoma of meninges, bacteriological or histological examination not done |
| 1312 | ICD9 | Tuberculoma of meninges, bacteriological or histological examination unknown (at present) |
| 1313 | ICD9 | Tuberculoma of meninges, tubercle bacilli found (in sputum) by microscopy |
| 1314 | ICD9 | Tuberculoma of meninges, tubercle bacilli not found (in sputum) by microscopy, but found by bacterial culture |
| 1315 | ICD9 | Tuberculoma of meninges, tubercle bacilli not found by bacteriological examination, but tuberculosis confirmed histologically |
| 1316 | ICD9 | Tuberculoma of meninges, tubercle bacilli not found by bacteriological or histological examination, but tuberculosis confirmed by other methods [inoculation of animals] |
| 1321 | ICD9 | Tuberculoma of brain, bacteriological or histological examination not done |
| 1322 | ICD9 | Tuberculoma of brain, bacteriological or histological examination unknown (at present) |
| 1323 | ICD9 | Tuberculoma of brain, tubercle bacilli found (in sputum) by microscopy |
| 1324 | ICD9 | Tuberculoma of brain, tubercle bacilli not found (in sputum) by microscopy, but found by bacterial culture |
| 1326 | ICD9 | Tuberculoma of brain, tubercle bacilli not found by bacteriological or histological examination, but tuberculosis confirmed by other |
| 1331 | ICD9 | Tuberculous abscess of brain, bacteriological or histological examination not done |
| 1332 | ICD9 | Tuberculous abscess of brain, bacteriological or histological examination unknown (at present) |
| 1333 | ICD9 | Tuberculous abscess of brain, tubercle bacilli found (in sputum) by microscopy |
| 1334 | ICD9 | Tuberculous abscess of brain, tubercle bacilli not found (in sputum) by microscopy, but found by bacterial culture |
| 1335 | ICD9 | Tuberculous abscess of brain, tubercle bacilli not found by bacteriological examination, but tuberculosis confirmed histologically |
| 1336 | ICD9 | Tuberculous abscess of brain, tubercle bacilli not found by bacteriological or histological examination, but tuberculosis confirmed by other methods [inoculation of animals] |
| 1360 | ICD9 | Tuberculous encephalitis or myelitis, unspecified |
| 1361 | ICD9 | Tuberculous encephalitis or myelitis, bacteriological or histological examination not done |
| 1362 | ICD9 | Tuberculous encephalitis or myelitis, bacteriological or histological examination unknown (at present) |
| 1363 | ICD9 | Tuberculous encephalitis or myelitis, tubercle bacilli found (in sputum) by microscopy |
| 1364 | ICD9 | Tuberculous encephalitis or myelitis, tubercle bacilli not found (in sputum) by microscopy, but found by bacterial culture |
| 1365 | ICD9 | Tuberculous encephalitis or myelitis, tubercle bacilli not found by bacteriological examination, but tuberculosis confirmed histologically |
| 1366 | ICD9 | Tuberculous encephalitis or myelitis, tubercle bacilli not found by bacteriological or histological examination, but tuberculosis confirmed by other methods [inoculation of animals] |
| 1380 | ICD9 | Other specified tuberculosis of central nervous system, unspecified |
| 1381 | ICD9 | Other specified tuberculosis of central nervous system, bacteriological or histological examination not done |
| 1382 | ICD9 | Other specified tuberculosis of central nervous system, bacteriological or histological examination unknown (at present) |
| 1383 | ICD9 | Other specified tuberculosis of central nervous system, tubercle bacilli found (in sputum) by microscopy |
| 1384 | ICD9 | Other specified tuberculosis of central nervous system, tubercle bacilli not found (in sputum) by microscopy, but found by bacterial culture |
| 1385 | ICD9 | Other specified tuberculosis of central nervous system, tubercle bacilli not found by bacteriological examination, but tuberculosis confirmed histologically |
| 1386 | ICD9 | Other specified tuberculosis of central nervous system, tubercle bacilli not found by bacteriological or histological examination, but tuberculosis confirmed by other methods [inoculation of animals] |
| 1390 | ICD9 | Unspecified tuberculosis of central nervous system, unspecified |
| 1391 | ICD9 | Unspecified tuberculosis of central nervous system, bacteriological or histological examination not done |
| 1392 | ICD9 | Unspecified tuberculosis of central nervous system, bacteriological or histological examination unknown (at present) |
| 1393 | ICD9 | Unspecified tuberculosis of central nervous system, tubercle bacilli found (in sputum) by microscopy |
| 1394 | ICD9 | Unspecified tuberculosis of central nervous system, tubercle bacilli not found (in sputum) by microscopy, but found by bacterial culture |
| 1395 | ICD9 | Unspecified tuberculosis of central nervous system, tubercle bacilli not found by bacteriological examination, but tuberculosis confirmed histologically |
| 1396 | ICD9 | Unspecified tuberculosis of central nervous system, tubercle bacilli not found by bacteriological or histological examination, but tuberculosis confirmed by other methods [inoculation of animals] |
| 1142 | ICD9 | Coccidioidal meningitis |
| 621 | ICD9 | Western equine encephalitis |
| 622 | ICD9 | Eastern equine encephalitis |
| 623 | ICD9 | St. Louis encephalitis |
| 624 | ICD9 | Australian encephalitis |
| 625 | ICD9 | California virus encephalitis |
| 632 | ICD9 | Central european encephalitis |
| 638 | ICD9 | Other specified tick-borne viral encephalitis |
| 3222 | ICD9 | Chronic meningitis |
| 3212 | ICD9 | Meningitis due to viruses not elsewhere classified |
| 3201 | ICD9 | Pneumococcal meningitis |
| A1782 | ICD10 | Tuberculous meningoencephalitis |
| A3212 | ICD10 | Listerial meningoencephalitis |
| A3981 | ICD10 | Meningococcal encephalitis |
| A4282 | ICD10 | Actinomycotic encephalitis |
| A5042 | ICD10 | Late congenital syphilitic encephalitis |
| A5214 | ICD10 | Late syphilitic encephalitis |
| A811 | ICD10 | Subacute sclerosing panencephalitis |
| A830 | ICD10 | Japanese encephalitis |
| A831 | ICD10 | Western equine encephalitis |
| A832 | ICD10 | Eastern equine encephalitis |
| A833 | ICD10 | St Louis encephalitis |
| A834 | ICD10 | Australian encephalitis |
| A835 | ICD10 | California encephalitis |
| A838 | ICD10 | Other mosquito-borne viral encephalitis |
| A839 | ICD10 | Mosquito-borne viral encephalitis, unspecified |
| A840 | ICD10 | Far Eastern tick-borne encephalitis [Russian spring-summer encephalitis] |
| A841 | ICD10 | Central European tick-borne encephalitis |
| A848 | ICD10 | Other tick-borne viral encephalitis |
| A849 | ICD10 | Tick-borne viral encephalitis, unspecified |
| A850 | ICD10 | Enteroviral encephalitis |
| A851 | ICD10 | Adenoviral encephalitis |
| A852 | ICD10 | Arthropod-borne viral encephalitis, unspecified |
| A858 | ICD10 | Other specified viral encephalitis |
| A86 | ICD10 | Unspecified viral encephalitis |
| A870 | ICD10 | Enteroviral meningitis |
| A871 | ICD10 | Adenoviral meningitis |
| A872 | ICD10 | Lymphocytic choriomeningitis |
| A878 | ICD10 | Other viral meningitis |
| A879 | ICD10 | Viral meningitis, unspecified |
| A880 | ICD10 | Enteroviral exanthematous fever [Boston exanthem] |
| A888 | ICD10 | Other specified viral infections of central nervous system |
| A89 | ICD10 | Unspecified viral infection of central nervous system |
| A90 | ICD10 | Dengue fever [classical dengue] |
| B004 | ICD10 | Herpesviral encephalitis |
| B0111 | ICD10 | Varicella encephalitis and encephalomyelitis |
| B020 | ICD10 | Zoster encephalitis |
| B050 | ICD10 | Measles complicated by encephalitis |
| B0601 | ICD10 | Rubella encephalitis |
| B1001 | ICD10 | Human herpesvirus 6 encephalitis |
| B1009 | ICD10 | Other human herpesvirus encephalitis |
| B262 | ICD10 | Mumps encephalitis |
| B4081 | ICD10 | Blastomycotic meningoencephalitis |
| B5742 | ICD10 | Meningoencephalitis in Chagas' disease |
| B582 | ICD10 | Toxoplasma meningoencephalitis |
| B6011 | ICD10 | Meningoencephalitis due to Acanthamoeba (culbertsoni) |
| B941 | ICD10 | Sequelae of viral encephalitis |
| G0400 | ICD10 | Acute disseminated encephalitis and encephalomyelitis, unspecified |
| G0401 | ICD10 | Postinfectious acute disseminated encephalitis and encephalomyelitis (postinfectious ADEM) |
| G0402 | ICD10 | Postimmunization acute disseminated encephalitis, myelitis and encephalomyelitis |
| G042 | ICD10 | Bacterial meningoencephalitis and meningomyelitis, not elsewhere classified |
| G0481 | ICD10 | Other encephalitis and encephalomyelitis |
| G0490 | ICD10 | Encephalitis and encephalomyelitis, unspecified |
| G361 | ICD10 | Acute and subacute hemorrhagic leukoencephalitis [Hurst] |
| A1782 | ICD10 | Tuberculous meningoencephalitis |
| A3212 | ICD10 | Listerial meningoencephalitis |
| A5141 | ICD10 | Secondary syphilitic meningitis |
| A5213 | ICD10 | Late syphilitic meningitis |
| A5481 | ICD10 | Gonococcal meningitis |
| A6921 | ICD10 | Meningitis due to Lyme disease |
| A870 | ICD10 | Enteroviral meningitis |
| A871 | ICD10 | Adenoviral meningitis |
| A872 | ICD10 | Lymphocytic choriomeningitis |
| A878 | ICD10 | Other viral meningitis |
| A879 | ICD10 | Viral meningitis, unspecified |
| A880 | ICD10 | Enteroviral exanthematous fever [Boston exanthem] |
| A888 | ICD10 | Other specified viral infections of central nervous system |
| A89 | ICD10 | Unspecified viral infection of central nervous system |
| B003 | ICD10 | Herpesviral meningitis |
| B010 | ICD10 | Varicella meningitis |
| B021 | ICD10 | Zoster meningitis |
| B051 | ICD10 | Measles complicated by meningitis |
| B0602 | ICD10 | Rubella meningitis |
| B261 | ICD10 | Mumps meningitis |
| B2702 | ICD10 | Gammaherpesviral mononucleosis with meningitis |
| B2712 | ICD10 | Cytomegaloviral mononucleosis with meningitis |
| B2782 | ICD10 | Other infectious mononucleosis with meningitis |
| B2792 | ICD10 | Infectious mononucleosis, unspecified with meningitis |
| B384 | ICD10 | Coccidioidomycosis meningitis |
| B5741 | ICD10 | Meningitis in Chagas' disease |
| D8681 | ICD10 | Sarcoid meningitis |
| G000 | ICD10 | Hemophilus meningitis |
| G001 | ICD10 | Pneumococcal meningitis |
| G002 | ICD10 | Streptococcal meningitis |
| G003 | ICD10 | Staphylococcal meningitis |
| G008 | ICD10 | Other bacterial meningitis |
| G009 | ICD10 | Bacterial meningitis, unspecified |
| G01 | ICD10 | Meningitis in bacterial diseases classified elsewhere |
| G02 | ICD10 | Meningitis in other infectious and parasitic diseases classified elsewhere |
| G030 | ICD10 | Nonpyogenic meningitis |
| G031 | ICD10 | Chronic meningitis |
| G032 | ICD10 | Benign recurrent meningitis [Mollaret] |
| G038 | ICD10 | Meningitis due to other specified causes |
| G039 | ICD10 | Meningitis, unspecified |
