# Supplementary material for: Propofol provides a significant survival advantage in sepsis-associated encephalopathy: A retrospective cohort study investigating one-year all-cause mortality
Source: PLoS One. 2026 Feb 5;21(2):e0340371. doi: 10.1371/journal.pone.0340371 (PMC12875438; doi:10.1371/journal.pone.0340371)
Supplement: S4 Table — (DOCX) [file pone.0340371.s004.docx]

Supporting Information

# **S4 Table.** Exclude patients with epilepsy disease from the MIMIC-IV database according to ICDcodes

| ICD-code | ICD | Description |
| --- | --- | --- |
| 34591 | ICD9 | Epilepsy, unspecified, with intractable epilepsy |
| 34570 | ICD9 | Epilepsia partialis continua, without mention of intractable epilepsy |
| 34571 | ICD9 | Epilepsia partialis continua, with intractable epilepsy |
| 34580 | ICD9 | Other forms of epilepsy and recurrent seizures, without mention of intractable epilepsy |
| 34581 | ICD9 | Other forms of epilepsy and recurrent seizures, with intractable epilepsy |
| 34590 | ICD9 | Epilepsy, unspecified, without mention of intractable epilepsy |
| 34591 | ICD9 | Epilepsy, unspecified, with intractable epilepsy |
| 64940 | ICD9 | Epilepsy complicating pregnancy, childbirth, or the puerperium, unspecified as to episode of care or not applicable |
| 64941 | ICD9 | Epilepsy complicating pregnancy, childbirth, or the puerperium, delivered, with or without mention of antepartum condition |
| 64942 | ICD9 | Epilepsy complicating pregnancy, childbirth, or the puerperium, delivered, with mention of postpartum complication |
| 64943 | ICD9 | Epilepsy complicating pregnancy, childbirth, or the puerperium, antepartum condition or complication |
| 64944 | ICD9 | Epilepsy complicating pregnancy, childbirth, or the puerperium, postpartum condition or complication |
| G40001 | ICD10 | Localization-related (focal) (partial) idiopathic epilepsy and epileptic syndromes with seizures of localized onset, not intractable, with status epilepticus |
| G40009 | ICD10 | Localization-related (focal) (partial) idiopathic epilepsy and epileptic syndromes with seizures of localized onset, not intractable, without status epilepticus |
| G40011 | ICD10 | Localization-related (focal) (partial) idiopathic epilepsy and epileptic syndromes with seizures of localized onset, intractable, with status epilepticus |
| G40019 | ICD10 | Localization-related (focal) (partial) idiopathic epilepsy and epileptic syndromes with seizures of localized onset, intractable, without status epilepticus |
| G40101 | ICD10 | Localization-related (focal) (partial) symptomatic epilepsy and epileptic syndromes with simple partial seizures, not intractable, with status epilepticus |
| G40109 | ICD10 | Localization-related (focal) (partial) symptomatic epilepsy and epileptic syndromes with simple partial seizures, not intractable, without status epilepticus |
| G40111 | ICD10 | Localization-related (focal) (partial) symptomatic epilepsy and epileptic syndromes with simple partial seizures, intractable, with status epilepticus |
| G40119 | ICD10 | Localization-related (focal) (partial) symptomatic epilepsy and epileptic syndromes with simple partial seizures, intractable, without status epilepticus |
| G40201 | ICD10 | Localization-related (focal) (partial) symptomatic epilepsy and epileptic syndromes with complex partial seizures, not intractable, with status epilepticus |
| G40209 | ICD10 | Localization-related (focal) (partial) symptomatic epilepsy and epileptic syndromes with complex partial seizures, not intractable, without status epilepticus |
| G40211 | ICD10 | Localization-related (focal) (partial) symptomatic epilepsy and epileptic syndromes with complex partial seizures, intractable, with status epilepticus |
| G40219 | ICD10 | Localization-related (focal) (partial) symptomatic epilepsy and epileptic syndromes with complex partial seizures, intractable, without status epilepticus |
| G40301 | ICD10 | Generalized idiopathic epilepsy and epileptic syndromes, not intractable, with status epilepticus |
| G40309 | ICD10 | Generalized idiopathic epilepsy and epileptic syndromes, not intractable, without status epilepticus |
| G40311 | ICD10 | Generalized idiopathic epilepsy and epileptic syndromes, intractable, with status epilepticus |
| G40319 | ICD10 | Generalized idiopathic epilepsy and epileptic syndromes, intractable, without status epilepticus |
| G40401 | ICD10 | Other generalized epilepsy and epileptic syndromes, not intractable, with status epilepticus |
| G40409 | ICD10 | Other generalized epilepsy and epileptic syndromes, not intractable, without status epilepticus |
| G40411 | ICD10 | Other generalized epilepsy and epileptic syndromes, intractable, with status epilepticus |
| G40419 | ICD10 | Other generalized epilepsy and epileptic syndromes, intractable, without status epilepticus |
| G40501 | ICD10 | Epileptic seizures related to external causes, not intractable, with status epilepticus |
| G40509 | ICD10 | Epileptic seizures related to external causes, not intractable, without status epilepticus |
| G40801 | ICD10 | Other epilepsy, not intractable, with status epilepticus |
| G40802 | ICD10 | Other epilepsy, not intractable, without status epilepticus |
| G40803 | ICD10 | Other epilepsy, intractable, with status epilepticus |
| G40804 | ICD10 | Other epilepsy, intractable, without status epilepticus |
| G40811 | ICD10 | Lennox-Gastaut syndrome, not intractable, with status epilepticus |
| G40812 | ICD10 | Lennox-Gastaut syndrome, not intractable, without status epilepticus |
| G40813 | ICD10 | Lennox-Gastaut syndrome, intractable, with status epilepticus |
| G40821 | ICD10 | Epileptic spasms, not intractable, with status epilepticus |
| G40822 | ICD10 | Epileptic spasms, not intractable, without status epilepticus |
| G40823 | ICD10 | Epileptic spasms, intractable, with status epilepticus |
| G40824 | ICD10 | Epileptic spasms, intractable, without status epilepticus |
| G4089 | ICD10 | Other seizures |
| G40901 | ICD10 | Epilepsy, unspecified, not intractable, with status epilepticus |
| G40909 | ICD10 | Epilepsy, unspecified, not intractable, without status epilepticus |
| G40911 | ICD10 | Epilepsy, unspecified, intractable, with status epilepticus |
| G40919 | ICD10 | Epilepsy, unspecified, intractable, without status epilepticus |
| G40A01 | ICD10 | Absence epileptic syndrome, not intractable, with status epilepticus |
| G40A09 | ICD10 | Absence epileptic syndrome, not intractable, without status epilepticus |
| G40A11 | ICD10 | Absence epileptic syndrome, intractable, with status epilepticus |
| G40A19 | ICD10 | Absence epileptic syndrome, intractable, without status epilepticus |
| G40B01 | ICD10 | Juvenile myoclonic epilepsy, not intractable, with status epilepticus |
| G40B09 | ICD10 | Juvenile myoclonic epilepsy, not intractable, without status epilepticus |
| G40B11 | ICD10 | Juvenile myoclonic epilepsy, intractable, with status epilepticus |
| G40B19 | ICD10 | Juvenile myoclonic epilepsy, intractable, without status epilepticus |
| G40A01 | ICD10 | Absence epileptic syndrome, not intractable, with status epilepticus |
| G40A09 | ICD10 | Absence epileptic syndrome, not intractable, without status epilepticus |
| G40A11 | ICD10 | Absence epileptic syndrome, intractable, with status epilepticus |
| G40A19 | ICD10 | Absence epileptic syndrome, intractable, without status epilepticus |
| G40B01 | ICD10 | Juvenile myoclonic epilepsy, not intractable, with status epilepticus |
| G40B09 | ICD10 | Juvenile myoclonic epilepsy, not intractable, without status epilepticus |
| G40B11 | ICD10 | Juvenile myoclonic epilepsy, intractable, with status epilepticus |
| G40B19 | ICD10 | Juvenile myoclonic epilepsy, intractable, without status epilepticus |
