# Supplementary material for: Propofol provides a significant survival advantage in sepsis-associated encephalopathy: A retrospective cohort study investigating one-year all-cause mortality
Source: PLoS One. 2026 Feb 5;21(2):e0340371. doi: 10.1371/journal.pone.0340371 (PMC12875438; doi:10.1371/journal.pone.0340371)
Supplement: S5 Table — (DOCX) [file pone.0340371.s005.docx]

Supporting Information

# **S5 Table.** Exclude patients with other cerebrovascular disease from the MIMIC-IV database according to ICD-codes

| ICD-code | ICD | Description |
| --- | --- | --- |
| 3312 | 9 | Senile degeneration of brain |
| 3313 | 9 | Communicating hydrocephalus |
| 3314 | 9 | Obstructive hydrocephalus |
| 33189 | 9 | Other cerebral degeneration |
| 3319 | 9 | Cerebral degeneration, unspecified |
| 3301 | 9 | Cerebral lipidoses |
| 3302 | 9 | Cerebral degeneration in generalized lipidoses |
| 3303 | 9 | Cerebral degeneration of childhood in other diseases classified elsewhere |
| 3308 | 9 | Other specified cerebral degenerations in childhood |
| 3309 | 9 | Unspecified cerebral degeneration in childhood |
| 3310 | 9 | Alzheimer's disease |
| 3317 | 9 | Cerebral degeneration in diseases classified elsewhere |
| 33189 | 9 | Other cerebral degeneration |
| 3319 | 9 | Cerebral degeneration, unspecified |
| 4378 | 9 | Other ill-defined cerebrovascular disease |
| 4379 | 9 | Unspecified cerebrovascular disease |
| 74100 | 9 | Spina bifida with hydrocephalus, unspecified region |
| 74101 | 9 | Spina bifida with hydrocephalus, cervical region |
| 74102 | 9 | Spina bifida with hydrocephalus, dorsal (thoracic) region |
| 74103 | 9 | Spina bifida with hydrocephalus, lumbar region |
| 74190 | 9 | Spina bifida without mention of hydrocephalus, unspecified region |
| 74191 | 9 | Spina bifida without mention of hydrocephalus, cervical region |
| 74192 | 9 | Spina bifida without mention of hydrocephalus, dorsal (thoracic) region |
| 74193 | 9 | Spina bifida without mention of hydrocephalus, lumbar region |
| 7423 | 9 | Coxsackie myocarditis |
| 4380 | 9 | Late effects of cerebrovascular disease, cognitive deficits |
| 43810 | 9 | Late effects of cerebrovascular disease, speech and language deficit, unspecified |
| 43811 | 9 | Late effects of cerebrovascular disease, aphasia |
| 43812 | 9 | Late effects of cerebrovascular disease, dysphasia |
| 43813 | 9 | Late effects of cerebrovascular disease, dysarthria |
| 43814 | 9 | Late effects of cerebrovascular disease, fluency disorder |
| 43819 | 9 | Late effects of cerebrovascular disease, other speech and language deficits |
| 43820 | 9 | Late effects of cerebrovascular disease, hemiplegia affecting unspecified side |
| 43821 | 9 | Late effects of cerebrovascular disease, hemiplegia affecting dominant side |
| 43822 | 9 | Late effects of cerebrovascular disease, hemiplegia affecting nondominant side |
| 43830 | 9 | Late effects of cerebrovascular disease, monoplegia of upper limb affecting unspecified side |
| 43831 | 9 | Late effects of cerebrovascular disease, monoplegia of upper limb affecting dominant side |
| 43832 | 9 | Late effects of cerebrovascular disease, monoplegia of upper limb affecting nondominant side |
| 43840 | 9 | Late effects of cerebrovascular disease, monoplegia of lower limb affecting unspecified side |
| 43841 | 9 | Late effects of cerebrovascular disease, monoplegia of lower limb affecting dominant side |
| 43842 | 9 | Late effects of cerebrovascular disease, monoplegia of lower limb affecting nondominant side |
| 43850 | 9 | Late effects of cerebrovascular disease, other paralytic syndrome affecting unspecified side |
| 43851 | 9 | Late effects of cerebrovascular disease, other paralytic syndrome affecting dominant side |
| 43852 | 9 | Late effects of cerebrovascular disease, other paralytic syndrome affecting nondominant side |
| 43853 | 9 | Late effects of cerebrovascular disease, other paralytic syndrome, bilateral |
| 4386 | 9 | Late effects of cerebrovascular disease, alterations of sensations |
| 4387 | 9 | Late effects of cerebrovascular disease, disturbances of vision |
| 43881 | 9 | Other late effects of cerebrovascular disease, apraxia |
| 43882 | 9 | Other late effects of cerebrovascular disease, dysphagia |
| 43883 | 9 | Other late effects of cerebrovascular disease, facial weakness |
| 43884 | 9 | Other late effects of cerebrovascular disease, ataxia |
| 43885 | 9 | Other late effects of cerebrovascular disease, vertigo |
| 43889 | 9 | Other late effects of cerebrovascular disease |
| 4380 | 9 | Unspecified cerebrovascular disease |
| 43810 | 9 | Late effects of cerebrovascular disease, speech and language deficit, unspecified |
| G311 | 10 | Senile degeneration of brain, not elsewhere classified |
| G910 | 10 | Communicating hydrocephalus |
| G911 | 10 | Obstructive hydrocephalus |
| G912 | 10 | (Idiopathic) normal pressure hydrocephalus |
| G913 | 10 | Post-traumatic hydrocephalus, unspecified |
| G914 | 10 | Hydrocephalus in diseases classified elsewhere |
| Q038 | 10 | Other congenital hydrocephalus |
| Q039 | 10 | Congenital hydrocephalus, unspecified |
| Q050 | 10 | Cervical spina bifida with hydrocephalus |
| Q051 | 10 | Thoracic spina bifida with hydrocephalus |
| Q052 | 10 | Lumbar spina bifida with hydrocephalus |
| Q053 | 10 | Sacral spina bifida with hydrocephalus |
| Q054 | 10 | Unspecified spina bifida with hydrocephalus |
| Q055 | 10 | Cervical spina bifida without hydrocephalus |
| Q056 | 10 | Thoracic spina bifida without hydrocephalus |
| Q057 | 10 | Lumbar spina bifida without hydrocephalus |
| Q058 | 10 | Sacral spina bifida without hydrocephalus |
| Q050 | 10 | Cervical spina bifida with hydrocephalus |
| Q0700 | 10 | Arnold-Chiari syndrome without spina bifida or hydrocephalus |
| Q0701 | 10 | Arnold-Chiari syndrome with spina bifida |
| Q0702 | 10 | Arnold-Chiari syndrome with hydrocephalus |
| Q0703 | 10 | Arnold-Chiari syndrome with spina bifida and hydrocephalus |
| Q078 | 10 | Other specified congenital malformations of nervous system |
| Q079 | 10 | Congenital malformation of nervous system, unspecified |
| A066 | 10 | Amebic brain abscess |
| A5482 | 10 | Gonococcal brain abscess |
| B431 | 10 | Pheomycotic brain abscess |
| C710 | 10 | Malignant neoplasm of cerebrum, except lobes and ventricles |
| C711 | 10 | Malignant neoplasm of frontal lobe |
| C712 | 10 | Malignant neoplasm of temporal lobe |
| C713 | 10 | Malignant neoplasm of parietal lobe |
| C714 | 10 | Malignant neoplasm of occipital lobe |
| C715 | 10 | Malignant neoplasm of cerebral ventricle |
| C716 | 10 | Malignant neoplasm of cerebellum |
| C717 | 10 | Malignant neoplasm of brain stem |
| C718 | 10 | Malignant neoplasm of overlapping sites of brain |
| C719 | 10 | Malignant neoplasm of brain, unspecified |
| C729 | 10 | Malignant neoplasm of central nervous system, unspecified |
| C7932 | 10 | Secondary malignant neoplasm of cerebral meninges |
| C7932 | 10 | Secondary malignant neoplasm of cerebral meninges |
| C7940 | 10 | Secondary malignant neoplasm of unspecified part of nervous system |
| C7949 | 10 | Secondary malignant neoplasm of other parts of nervous system |
| D320 | 10 | Benign neoplasm of cerebral meninges |
| D321 | 10 | Benign neoplasm of spinal meninges |
| D329 | 10 | Benign neoplasm of meninges, unspecified |
| D330 | 10 | Benign neoplasm of brain, supratentorial |
| D331 | 10 | Benign neoplasm of brain, infratentorial |
| D332 | 10 | Benign neoplasm of brain, unspecified |
| D333 | 10 | Benign neoplasm of cranial nerves |
| D337 | 10 | Benign neoplasm of other specified parts of central nervous system |
| D339 | 10 | Benign neoplasm of central nervous system, unspecified |
| D420 | 10 | Neoplasm of uncertain behavior of cerebral meninges |
| D430 | 10 | Neoplasm of uncertain behavior of brain, supratentorial |
| D431 | 10 | Neoplasm of uncertain behavior of brain, infratentorial |
| D432 | 10 | Neoplasm of uncertain behavior of brain, unspecified |
| D433 | 10 | Neoplasm of uncertain behavior of cranial nerves |
| D438 | 10 | Neoplasm of uncertain behavior of other specified parts of central nervous system |
| D439 | 10 | Neoplasm of uncertain behavior of central nervous system, unspecified |
| D496 | 10 | Neoplasm of unspecified behavior of brain |
| V1085 | 9 | Personal history of malignant neoplasm of brain |
| V1086 | 9 | Personal history of malignant neoplasm of other parts of nervous system |
| V1241 | 9 | Personal history of benign neoplasm of the brain |
| V1242 | 9 | Personal history of infections of the central nervous system |
| Z1282 | 10 | Encounter for screening for malignant neoplasm of nervous system |
| Z85841 | 10 | Personal history of malignant neoplasm of brain |
| Z8603 | 10 | Personal history of neoplasm of uncertain behavior |
