# Supplementary material for: Propofol provides a significant survival advantage in sepsis-associated encephalopathy: A retrospective cohort study investigating one-year all-cause mortality
Source: PLoS One. 2026 Feb 5;21(2):e0340371. doi: 10.1371/journal.pone.0340371 (PMC12875438; doi:10.1371/journal.pone.0340371)
Supplement: S6 Table — (DOCX) [file pone.0340371.s006.docx]

Supporting Information

# S6 Table. Exclude patients with mental disorders and and neurological disease from the MIMIC-IV database according to ICD-codes

| ICD-code | ICD | Description |
| --- | --- | --- |
| 29634 | 9 | Major depressive affective disorder, recurrent episode, severe, specified as with psychotic behavior |
| 29635 | 9 | Major depressive affective disorder, recurrent episode, in partial or unspecified remission |
| 29636 | 9 | Major depressive affective disorder, recurrent episode, in full remission |
| 29640 | 9 | Bipolar I disorder, most recent episode (or current) manic, unspecified |
| 29641 | 9 | Bipolar I disorder, most recent episode (or current) manic, mild |
| 29642 | 9 | Bipolar I disorder, most recent episode (or current) manic, moderate |
| 29643 | 9 | Bipolar I disorder, most recent episode (or current) manic, severe, without mention of psychotic behavior |
| 29644 | 9 | Bipolar I disorder, most recent episode (or current) manic, severe, specified as with psychotic behavior |
| 29645 | 9 | Bipolar I disorder, most recent episode (or current) manic, in partial or unspecified remission |
| 29646 | 9 | Bipolar I disorder, most recent episode (or current) manic, in full remission |
| 29650 | 9 | Bipolar I disorder, most recent episode (or current) depressed, unspecified |
| 29651 | 9 | Bipolar I disorder, most recent episode (or current) depressed, mild |
| 29652 | 9 | Bipolar I disorder, most recent episode (or current) depressed, moderate |
| 29653 | 9 | Bipolar I disorder, most recent episode (or current) depressed, severe, without mention of psychotic behavior |
| 29654 | 9 | Bipolar I disorder, most recent episode (or current) depressed, severe, specified as with psychotic behavior |
| 29655 | 9 | Bipolar I disorder, most recent episode (or current) depressed, in partial or unspecified remission |
| 29656 | 9 | Bipolar I disorder, most recent episode (or current) depressed, in full remission |
| 29660 | 9 | Bipolar I disorder, most recent episode (or current) mixed, unspecified |
| 29661 | 9 | Bipolar I disorder, most recent episode (or current) mixed, mild |
| 29662 | 9 | Bipolar I disorder, most recent episode (or current) mixed, moderate |
| 29620 | 9 | Major depressive affective disorder, single episode, unspecified |
| 29621 | 9 | Major depressive affective disorder, single episode, mild |
| 29622 | 9 | Major depressive affective disorder, single episode, moderate |
| 29623 | 9 | Major depressive affective disorder, single episode, severe, without mention of psychotic behavior |
| 29624 | 9 | Major depressive affective disorder, single episode, severe, specified as with psychotic behavior |
| 29625 | 9 | Major depressive affective disorder, single episode, in partial or unspecified remission |
| 29626 | 9 | Major depressive affective disorder, single episode, in full remission |
| 29630 | 9 | Major depressive affective disorder, recurrent episode, unspecified |
| 29631 | 9 | Major depressive affective disorder, recurrent episode, mild |
| 29632 | 9 | Major depressive affective disorder, recurrent episode, moderate |
| 29633 | 9 | Major depressive affective disorder, recurrent episode, severe, without mention of psychotic behavior |
| 29634 | 9 | Major depressive affective disorder, recurrent episode, severe, specified as with psychotic behavior |
| 29635 | 9 | Major depressive affective disorder, recurrent episode, in partial or unspecified remission |
| 29636 | 9 | Major depressive affective disorder, recurrent episode, in full remission |
| 29663 | 9 | Bipolar I disorder, most recent episode (or current) mixed, severe, without mention of psychotic behavior |
| 29664 | 9 | Bipolar I disorder, most recent episode (or current) mixed, severe, specified as with psychotic behavior |
| 29665 | 9 | Bipolar I disorder, most recent episode (or current) mixed, in partial or unspecified remission |
| 29666 | 9 | Bipolar I disorder, most recent episode (or current) mixed, in full remission |
| 2967 | 9 | Bipolar I disorder, most recent episode (or current) unspecified |
| 29680 | 9 | Bipolar disorder, unspecified |
| 29681 | 9 | Atypical manic disorder |
| 29682 | 9 | Atypical depressive disorder |
| 29689 | 9 | Other bipolar disorders |
| 30289 | 9 | Other specified psychosexual disorders |
| 3029 | 9 | Unspecified psychosexual disorder |
| 29682 | 9 | Atypical depressive disorder |
| 2971 | 9 | Delusional disorder |
| 2972 | 9 | Paraphrenia |
| 2980 | 9 | Depressive type psychosis |
| 2981 | 9 | Excitative type psychosis |
| 2982 | 9 | Reactive confusion |
| 2983 | 9 | Acute paranoid reaction |
| 2984 | 9 | Psychogenic paranoid psychosis |
| 2988 | 9 | Other and unspecified reactive psychosis |
| 2989 | 9 | Unspecified psychosis |
| 30111 | 9 | Chronic hypomanic personality disorder |
| 30020 | 9 | Phobia, unspecified |
| 30021 | 9 | Agoraphobia with panic disorder |
| 30022 | 9 | Agoraphobia without mention of panic attacks |
| 30023 | 9 | Social phobia |
| 30029 | 9 | Other isolated or specific phobias |
| 30112 | 9 | Chronic depressive personality disorder |
| 30113 | 9 | Cyclothymic disorder |
| 30120 | 9 | Schizoid personality disorder, unspecified |
| 29381 | 9 | Psychotic disorder with delusions in conditions classified elsewhere |
| 29410 | 9 | Dementia in conditions classified elsewhere without behavioral disturbance |
| 29411 | 9 | Dementia in conditions classified elsewhere with behavioral disturbance |
| 29420 | 9 | Dementia, unspecified, without behavioral disturbance |
| 29421 | 9 | Dementia, unspecified, with behavioral disturbance |
| 2949 | 9 | Unspecified persistent mental disorders due to conditions classified elsewhere |
| 29500 | 9 | Simple type schizophrenia, unspecified |
| 29501 | 9 | Simple type schizophrenia, subchronic |
| 29502 | 9 | Simple type schizophrenia, chronic |
| 29503 | 9 | Simple type schizophrenia, subchronic with acute exacerbation |
| 29504 | 9 | Simple type schizophrenia, chronic with acute exacerbation |
| 29505 | 9 | Simple type schizophrenia, in remission |
| 29510 | 9 | Disorganized type schizophrenia, unspecified |
| 29511 | 9 | Disorganized type schizophrenia, subchronic |
| 29512 | 9 | Disorganized type schizophrenia, chronic |
| 29513 | 9 | Disorganized type schizophrenia, subchronic with acute exacerbation |
| 29514 | 9 | Disorganized type schizophrenia, chronic with acute exacerbation |
| 29515 | 9 | Disorganized type schizophrenia, in remission |
| 29520 | 9 | Catatonic type schizophrenia, unspecified |
| 29521 | 9 | Catatonic type schizophrenia, subchronic |
| 29522 | 9 | Catatonic type schizophrenia, chronic |
| 29523 | 9 | Catatonic type schizophrenia, subchronic with acute exacerbation |
| 29524 | 9 | Catatonic type schizophrenia, chronic with acute exacerbation |
| 29525 | 9 | Catatonic type schizophrenia, in remission |
| 29530 | 9 | Paranoid type schizophrenia, unspecified |
| 29531 | 9 | Paranoid type schizophrenia, subchronic |
| 29532 | 9 | Paranoid type schizophrenia, chronic |
| 29533 | 9 | Paranoid type schizophrenia, subchronic with acute exacerbation |
| 29534 | 9 | Paranoid type schizophrenia, chronic with acute exacerbation |
| 29535 | 9 | Paranoid type schizophrenia, in remission |
| 29540 | 9 | Schizophreniform disorder, unspecified |
| 29541 | 9 | Schizophreniform disorder, subchronic |
| 29542 | 9 | Schizophreniform disorder, chronic |
| 29543 | 9 | Schizophreniform disorder, subchronic with acute exacerbation |
| 29544 | 9 | Schizophreniform disorder, chronic with acute exacerbation |
| 29545 | 9 | Schizophreniform disorder, in remission |
| 29550 | 9 | Latent schizophrenia, unspecified |
| 29551 | 9 | Latent schizophrenia, unspecified |
| 29552 | 9 | Latent schizophrenia, chronic |
| 29553 | 9 | Latent schizophrenia, subchronic with acute exacerbation |
| 29554 | 9 | Latent schizophrenia, chronic with acute exacerbation |
| 29555 | 9 | Latent schizophrenia, in remission |
| 29560 | 9 | Schizophrenic disorders, residual type, unspecified |
| 29561 | 9 | Schizophrenic disorders, residual type, subchronic |
| 29562 | 9 | Schizophrenic disorders, residual type, chronic |
| 29563 | 9 | Schizophrenic disorders, residual type, subchronic with acute exacerbation |
| 29564 | 9 | Schizophrenic disorders, residual type, chronic with acute exacerbation |
| 29565 | 9 | Schizophrenic disorders, residual type, in remission |
| 29570 | 9 | Schizoaffective disorder, unspecified |
| 29571 | 9 | Schizoaffective disorder, subchronic |
| 29572 | 9 | Schizoaffective disorder, chronic |
| 29573 | 9 | Schizoaffective disorder, subchronic with acute exacerbation |
| 29574 | 9 | Schizoaffective disorder, chronic with acute exacerbation |
| 29580 | 9 | Other specified types of schizophrenia, unspecified |
| 29581 | 9 | Other specified types of schizophrenia, subchronic |
| 29582 | 9 | Other specified types of schizophrenia, chronic |
| 29583 | 9 | Other specified types of schizophrenia, subchronic with acute exacerbation |
| 29584 | 9 | Other specified types of schizophrenia, chronic with acute exacerbation |
| 29585 | 9 | Other specified types of schizophrenia, in remission |
| 29590 | 9 | Unspecified schizophrenia, unspecified |
| 29591 | 9 | Unspecified schizophrenia, subchronic |
| 29592 | 9 | Unspecified schizophrenia, chronic |
| 29593 | 9 | Unspecified schizophrenia, subchronic with acute exacerbation |
| 29594 | 9 | Unspecified schizophrenia, chronic with acute exacerbation |
| 29595 | 9 | Unspecified schizophrenia, in remission |
| 29600 | 9 | Bipolar I disorder, single manic episode, unspecified |
| 29601 | 9 | Bipolar I disorder, single manic episode, mild |
| 29602 | 9 | Bipolar I disorder, single manic episode, moderate |
| 29603 | 9 | Bipolar I disorder, single manic episode, severe, without mention of psychotic behavior |
| 29604 | 9 | Bipolar I disorder, single manic episode, severe, specified as with psychotic behavior |
| 29605 | 9 | Bipolar I disorder, single manic episode, in partial or unspecified remission |
| 29606 | 9 | Bipolar I disorder, single manic episode, in full remission |
| 29610 | 9 | Manic affective disorder, recurrent episode, unspecified |
| 29611 | 9 | Manic affective disorder, recurrent episode, mild |
| 29612 | 9 | Manic affective disorder, recurrent episode, moderate |
| 29613 | 9 | Manic affective disorder, recurrent episode, severe, without mention of psychotic behavior |
| 29614 | 9 | Manic affective disorder, recurrent episode, severe, specified as with psychotic behavior |
| 29615 | 9 | Manic affective disorder, recurrent episode, in partial or unspecified remission |
| 29616 | 9 | Manic affective disorder, recurrent episode, in full remission |
| 29620 | 9 | Major depressive affective disorder, single episode, unspecified |
| 29621 | 9 | Major depressive affective disorder, single episode, mild |
| 29622 | 9 | Major depressive affective disorder, single episode, moderate |
| 29623 | 9 | Major depressive affective disorder, single episode, severe, without mention of psychotic behavior |
| 29624 | 9 | Major depressive affective disorder, single episode, severe, specified as with psychotic behavior |
| 29625 | 9 | Major depressive affective disorder, single episode, in partial or unspecified remission |
| 29626 | 9 | Major depressive affective disorder, single episode, in full remission |
| 29630 | 9 | Major depressive affective disorder, recurrent episode, unspecified |
| 29631 | 9 | Major depressive affective disorder, recurrent episode, mild |
| 29632 | 9 | Major depressive affective disorder, recurrent episode, moderate |
| 29633 | 9 | Major depressive affective disorder, recurrent episode, severe, without mention of psychotic behavior |
| 33182 | 9 | Dementia with lewy bodies |
| 3310 | 9 | Alzheimer's disease |
| 33119 | 9 | Other frontotemporal dementia |
| 64842 | 9 | Mental disorders of mother, delivered, with mention of postpartum complication |
| 64843 | 9 | Mental disorders of mother, antepartum condition or complication |
| 64844 | 9 | Mental disorders of mother, postpartum condition or complication |
| 3181 | 9 | Severe intellectual disabilities |
| 3182 | 9 | Profound intellectual disabilities |
| 33182 | 9 | Dementia with lewy bodies |
| F0150 | 10 | Vascular dementia without behavioral disturbance |
| F0151 | 10 | Vascular dementia with behavioral disturbance |
| F0280 | 10 | Dementia in other diseases classified elsewhere without behavioral disturbance |
| F0281 | 10 | Dementia in other diseases classified elsewhere with behavioral disturbance |
| F0390 | 10 | Unspecified dementia without behavioral disturbance |
| F0391 | 10 | Unspecified dementia with behavioral disturbance |
| F04 | 10 | Amnestic disorder due to known physiological condition |
| F05 | 10 | Delirium due to known physiological condition |
| F060 | 10 | Psychotic disorder with hallucinations due to known physiological condition |
| F061 | 10 | Catatonic disorder due to known physiological condition |
| F062 | 10 | Psychotic disorder with delusions due to known physiological condition |
| F0630 | 10 | Mood disorder due to known physiological condition, unspecified |
| F0631 | 10 | Mood disorder due to known physiological condition with depressive features |
| F0632 | 10 | Mood disorder due to known physiological condition with major depressive-like episode |
| F0633 | 10 | Mood disorder due to known physiological condition with manic features |
| F0634 | 10 | Mood disorder due to known physiological condition with mixed features |
| F064 | 10 | Anxiety disorder due to known physiological condition |
| F068 | 10 | Other specified mental disorders due to known physiological condition |
| F070 | 10 | Personality change due to known physiological condition |
| F0781 | 10 | Postconcussional syndrome |
| F0789 | 10 | Other personality and behavioral disorders due to known physiological condition |
| F079 | 10 | Unspecified personality and behavioral disorder due to known physiological condition |
| F09 | 10 | Unspecified mental disorder due to known physiological condition |
| F200 | 10 | Paranoid schizophrenia |
| F201 | 10 | Disorganized schizophrenia |
| F202 | 10 | Catatonic schizophrenia |
| F203 | 10 | Undifferentiated schizophrenia |
| F205 | 10 | Residual schizophrenia |
| F2081 | 10 | Schizophreniform disorder |
| F2089 | 10 | Other schizophrenia |
| F209 | 10 | Schizophrenia, unspecified |
| F21 | 10 | Schizotypal disorder |
| F22 | 10 | Delusional disorders |
| F23 | 10 | Brief psychotic disorder |
| F24 | 10 | Shared psychotic disorder |
| F250 | 10 | Schizoaffective disorder, bipolar type |
| F251 | 10 | Schizoaffective disorder, depressive type |
| F258 | 10 | Other schizoaffective disorders |
| F259 | 10 | Schizoaffective disorder, unspecified |
| F28 | 10 | Other psychotic disorder not due to a substance or known physiological condition |
| F29 | 10 | Unspecified psychosis not due to a substance or known physiological condition |
| F3010 | 10 | Manic episode without psychotic symptoms, unspecified |
| F3011 | 10 | Manic episode without psychotic symptoms, mild |
| F3012 | 10 | Manic episode without psychotic symptoms, moderate |
| F3013 | 10 | Manic episode, severe, without psychotic symptoms |
| F302 | 10 | Manic episode, severe with psychotic symptoms |
| F303 | 10 | Manic episode in partial remission |
| F304 | 10 | Manic episode in full remission |
| F308 | 10 | Other manic episodes |
| F309 | 10 | Manic episode, unspecified |
| F310 | 10 | Bipolar disorder, current episode hypomanic |
| F3110 | 10 | Bipolar disorder, current episode manic without psychotic features, unspecified |
| F3111 | 10 | Bipolar disorder, current episode manic without psychotic features, mild |
| F3112 | 10 | Bipolar disorder, current episode manic without psychotic features, moderate |
| F3113 | 10 | Bipolar disorder, current episode manic without psychotic features, severe |
| F312 | 10 | Bipolar disorder, current episode manic severe with psychotic features |
| F3130 | 10 | Bipolar disorder, current episode depressed, mild or moderate severity, unspecified |
| F3131 | 10 | Bipolar disorder, current episode depressed, mild |
| F3132 | 10 | Bipolar disorder, current episode depressed, moderate |
| F314 | 10 | Bipolar disorder, current episode depressed, severe, without psychotic features |
| F315 | 10 | Bipolar disorder, current episode depressed, severe, with psychotic features |
| F3160 | 10 | Bipolar disorder, current episode mixed, unspecified |
| F3161 | 10 | Bipolar disorder, current episode mixed, mild |
| F3162 | 10 | Bipolar disorder, current episode mixed, moderate |
| F3163 | 10 | Bipolar disorder, current episode mixed, severe, without psychotic features |
| F3164 | 10 | Bipolar disorder, current episode mixed, severe, with psychotic features |
| F3170 | 10 | Bipolar disorder, currently in remission, most recent episode unspecified |
| F3171 | 10 | Bipolar disorder, in partial remission, most recent episode hypomanic |
| F3172 | 10 | Bipolar disorder, in full remission, most recent episode hypomanic |
| F3173 | 10 | Bipolar disorder, in partial remission, most recent episode manic |
| F3174 | 10 | Bipolar disorder, in full remission, most recent episode manic |
| F3175 | 10 | Bipolar disorder, in partial remission, most recent episode depressed |
| F3176 | 10 | Bipolar disorder, in full remission, most recent episode depressed |
| F3177 | 10 | Bipolar disorder, in partial remission, most recent episode mixed |
| F3178 | 10 | Bipolar disorder, in full remission, most recent episode mixed |
| F3181 | 10 | Bipolar II disorder |
| F3189 | 10 | Other bipolar disorder |
| F319 | 10 | Bipolar disorder, unspecified |
| F320 | 10 | Major depressive disorder, single episode, mild |
| F321 | 10 | Major depressive disorder, single episode, moderate |
| F322 | 10 | Major depressive disorder, single episode, severe without psychotic features |
| F323 | 10 | Major depressive disorder, single episode, severe with psychotic features |
| F324 | 10 | Major depressive disorder, single episode, in partial remission |
| F325 | 10 | Major depressive disorder, single episode, in full remission |
| F3281 | 10 | Premenstrual dysphoric disorder |
| F3289 | 10 | Other specified depressive episodes |
| F329 | 10 | Major depressive disorder, single episode, unspecified |
| F330 | 10 | Major depressive disorder, recurrent, mild |
| F331 | 10 | Major depressive disorder, recurrent, moderate |
| F332 | 10 | Major depressive disorder, recurrent severe without psychotic features |
| F333 | 10 | Major depressive disorder, recurrent, severe with psychotic symptoms |
| F3340 | 10 | Major depressive disorder, recurrent, in remission, unspecified |
| F3341 | 10 | Major depressive disorder, recurrent, in partial remission |
| F3342 | 10 | Major depressive disorder, recurrent, in full remission |
| F338 | 10 | Other recurrent depressive disorders |
| F340 | 10 | Cyclothymic disorder |
| F341 | 10 | Dysthymic disorder |
| F3481 | 10 | Disruptive mood dysregulation disorder |
| F3489 | 10 | Other specified persistent mood disorders |
| F349 | 10 | Persistent mood [affective] disorder, unspecified |
| F39 | 10 | Unspecified mood [affective] disorder |
| F4000 | 10 | Agoraphobia, unspecified |
| F4001 | 10 | Agoraphobia with panic disorder |
| F4002 | 10 | Agoraphobia without panic disorder |
| F4010 | 10 | Social phobia, unspecified |
| F4011 | 10 | Social phobia, generalized |
| F40248 | 10 | Other situational type phobia |
| F40290 | 10 | Androphobia |
| F40291 | 10 | Gynephobia |
| F40298 | 10 | Other specified phobia |
| F408 | 10 | Other phobic anxiety disorders |
| F409 | 10 | Phobic anxiety disorder, unspecified |
| F410 | 10 | Panic disorder [episodic paroxysmal anxiety] |
| F411 | 10 | Generalized anxiety disorder |
| F413 | 10 | Other mixed anxiety disorders |
| F418 | 10 | Other specified anxiety disorders |
| F419 | 10 | Anxiety disorder, unspecified |
| F422 | 10 | Mixed obsessional thoughts and acts |
| F423 | 10 | Hoarding disorder |
| F424 | 10 | Excoriation (skin-picking) disorder |
| F428 | 10 | Other obsessive-compulsive disorder |
| F429 | 10 | Obsessive-compulsive disorder, unspecified |
| F430 | 10 | Acute stress reaction |
| F4310 | 10 | Post-traumatic stress disorder, unspecified |
| F4311 | 10 | Post-traumatic stress disorder, acute |
| F4312 | 10 | Post-traumatic stress disorder, chronic |
| F4320 | 10 | Adjustment disorder, unspecified |
| F4321 | 10 | Adjustment disorder with depressed mood |
| F4322 | 10 | Adjustment disorder with anxiety |
| F4323 | 10 | Adjustment disorder with mixed anxiety and depressed mood |
| F4324 | 10 | Adjustment disorder with disturbance of conduct |
| F4325 | 10 | Adjustment disorder with mixed disturbance of emotions and conduct |
| F4329 | 10 | Adjustment disorder with other symptoms |
| F70 | 10 | Mild intellectual disabilities |
| F71 | 10 | Moderate intellectual disabilities |
| F72 | 10 | Severe intellectual disabilities |
| F73 | 10 | Profound intellectual disabilities |
| F78 | 10 | Other intellectual disabilities |
| F79 | 10 | Unspecified intellectual disabilities |
| F800 | 10 | Phonological disorder |
| F801 | 10 | Expressive language disorder |
| F802 | 10 | Mixed receptive-expressive language disorder |
| F804 | 10 | Speech and language development delay due to hearing loss |
| F8081 | 10 | Childhood onset fluency disorder |
| F8082 | 10 | Social pragmatic communication disorder |
| F8089 | 10 | Other developmental disorders of speech and language |
| F809 | 10 | Developmental disorder of speech and language, unspecified |
| F812 | 10 | Mathematics disorder |
| F8181 | 10 | Disorder of written expression |
| G300 | 10 | Alzheimer's disease with early onset |
| G301 | 10 | Alzheimer's disease with late onset |
| G308 | 10 | Other Alzheimer's disease |
| G309 | 10 | Alzheimer's disease, unspecified |
| G3101 | 10 | Pick's disease |
| G3109 | 10 | Other frontotemporal dementia |
| G311 | 10 | Senile degeneration of brain, not elsewhere classified |
| G312 | 10 | Degeneration of nervous system due to alcohol |
| G3101 | 10 | Pick's disease |
| G3109 | 10 | Other frontotemporal dementia |
| G311 | 10 | Senile degeneration of brain, not elsewhere classified |
| G312 | 10 | Degeneration of nervous system due to alcohol |
| G3181 | 10 | Alpers disease |
| G3182 | 10 | Leigh's disease |
| G3183 | 10 | Dementia with Lewy bodies |
| G360 | 10 | Neuromyelitis optica [Devic] |
| G361 | 10 | Acute and subacute hemorrhagic leukoencephalitis [Hurst] |
| G368 | 10 | Other specified acute disseminated demyelination |
| G369 | 10 | Acute disseminated demyelination, unspecified |
| G370 | 10 | Diffuse sclerosis of central nervous system |
| G371 | 10 | Central demyelination of corpus callosum |
| G372 | 10 | Central pontine myelinolysis |
| G373 | 10 | Acute transverse myelitis in demyelinating disease of central nervous system |
| G374 | 10 | Subacute necrotizing myelitis of central nervous system |
| G375 | 10 | Concentric sclerosis [Balo] of central nervous system |
| G378 | 10 | Other specified demyelinating diseases of central nervous system |
| G379 | 10 | Demyelinating disease of central nervous system, unspecified |
