# Supplementary material for: Propofol provides a significant survival advantage in sepsis-associated encephalopathy: A retrospective cohort study investigating one-year all-cause mortality
Source: PLoS One. 2026 Feb 5;21(2):e0340371. doi: 10.1371/journal.pone.0340371 (PMC12875438; doi:10.1371/journal.pone.0340371)
Supplement: S7 Table — (DOCX) [file pone.0340371.s007.docx]

Supporting Information

# **S7 Table.** Exclude patients with alcoholic intoxication or or drug abuse from the MIMIC-IV database according to ICD-codes

| ICD-code | ICD | Description |
| --- | --- | --- |
| 30303 | 9 | Acute alcoholic intoxication in alcoholism, in remission |
| 30390 | 9 | Other and unspecified alcohol dependence, unspecified |
| 30391 | 9 | Other and unspecified alcohol dependence, continuous |
| 30392 | 9 | Other and unspecified alcohol dependence, episodic |
| 30393 | 9 | Other and unspecified alcohol dependence, in remission |
| 30400 | 9 | Opioid type dependence, unspecified |
| 30401 | 9 | Opioid type dependence, continuous |
| 30300 | 9 | Acute alcoholic intoxication in alcoholism, unspecified |
| 30301 | 9 | Acute alcoholic intoxication in alcoholism, continuous |
| 30302 | 9 | Acute alcoholic intoxication in alcoholism, episodic |
| 30402 | 9 | Opioid type dependence, episodic |
| 30403 | 9 | Opioid type dependence, in remission |
| 30410 | 9 | Sedative, hypnotic or anxiolytic dependence, unspecified |
| 30411 | 9 | Sedative, hypnotic or anxiolytic dependence, continuous |
| 30412 | 9 | Sedative, hypnotic or anxiolytic dependence, episodic |
| 30413 | 9 | Sedative, hypnotic or anxiolytic dependence, in remission |
| 30420 | 9 | Cocaine dependence, unspecified |
| 30421 | 9 | Cocaine dependence, continuous |
| 30422 | 9 | Cocaine dependence, episodic |
| 30423 | 9 | Cocaine dependence, in remission |
| 30430 | 9 | Cannabis dependence, unspecified |
| 30431 | 9 | Cannabis dependence, continuous |
| 30432 | 9 | Cannabis dependence, episodic |
| 30433 | 9 | Cannabis dependence, in remission |
| 30440 | 9 | Amphetamine and other psychostimulant dependence, unspecified |
| 30441 | 9 | Amphetamine and other psychostimulant dependence, continuous |
| 30442 | 9 | Amphetamine and other psychostimulant dependence, episodic |
| 30443 | 9 | Amphetamine and other psychostimulant dependence, in remission |
| 30450 | 9 | Hallucinogen dependence, unspecified |
| 30451 | 9 | Hallucinogen dependence, continuous |
| 30452 | 9 | Hallucinogen dependence, episodic |
| 30453 | 9 | Hallucinogen dependence, in remission |
| 30460 | 9 | Other specified drug dependence, unspecified |
| Z7141 | 10 | Alcohol abuse counseling and surveillance of alcoholic |
| Z811 | 10 | Family history of alcohol abuse and dependence |
| 2910 | 9 | Alcohol withdrawal delirium |
| 2911 | 9 | Alcohol-induced persisting amnestic disorder |
| 2912 | 9 | Alcohol-induced persisting dementia |
| 2913 | 9 | Alcohol-induced psychotic disorder with hallucinations |
| 2914 | 9 | Idiosyncratic alcohol intoxication |
| 2915 | 9 | Alcohol-induced psychotic disorder with delusions |
| 29181 | 9 | Alcohol withdrawal |
| 29182 | 9 | Alcohol induced sleep disorders |
| 29189 | 9 | Other alcohol-induced mental disorders |
| 2919 | 9 | Unspecified alcohol-induced mental disorders |
| 3051 | 9 | Tobacco use disorder |
| 30520 | 9 | Cannabis abuse, unspecified |
| 30521 | 9 | Cannabis abuse, continuous |
| 30522 | 9 | Cannabis abuse, episodic |
| 30523 | 9 | Cannabis abuse, in remission |
| 30530 | 9 | Hallucinogen abuse, unspecified |
| 30531 | 9 | Hallucinogen abuse, continuous |
| 30532 | 9 | Hallucinogen abuse, episodic |
| 30533 | 9 | Hallucinogen abuse, in remission |
| 30540 | 9 | Sedative, hypnotic or anxiolytic abuse, unspecified |
| 30541 | 9 | Sedative, hypnotic or anxiolytic abuse, continuous |
| 30542 | 9 | Sedative, hypnotic or anxiolytic abuse, episodic |
| 30543 | 9 | Sedative, hypnotic or anxiolytic abuse, in remission |
| 30550 | 9 | Opioid abuse, unspecified |
| 30551 | 9 | Opioid abuse, continuous |
| 30552 | 9 | Opioid abuse, episodic |
| 30553 | 9 | Opioid abuse, in remission |
| 30560 | 9 | Cocaine abuse, unspecified |
| 30561 | 9 | Cocaine abuse, continuous |
| 30562 | 9 | Cocaine abuse, episodic |
| 30563 | 9 | Cocaine abuse, in remission |
| 30570 | 9 | Amphetamine or related acting sympathomimetic abuse, unspecified |
| 30571 | 9 | Amphetamine or related acting sympathomimetic abuse, continuous |
| 30572 | 9 | Amphetamine or related acting sympathomimetic abuse, episodic |
| 30573 | 9 | Amphetamine or related acting sympathomimetic abuse, in remission |
| 30580 | 9 | Antidepressant type abuse, unspecified |
| 30581 | 9 | Antidepressant type abuse, continuous |
| 30582 | 9 | Antidepressant type abuse, episodic |
| 30583 | 9 | Antidepressant type abuse, in remission |
| 64900 | 9 | Tobacco use disorder complicating pregnancy, childbirth, or the puerperium, unspecified as to episode of care or not applicable |
| 64901 | 9 | Tobacco use disorder complicating pregnancy, childbirth, or the puerperium, delivered, with or without mention of antepartum condition |
| 64902 | 9 | Tobacco use disorder complicating pregnancy, childbirth, or the puerperium, delivered, with mention of postpartum complication |
| 64903 | 9 | Tobacco use disorder complicating pregnancy, childbirth, or the puerperium, antepartum condition or complication |
| 64904 | 9 | Tobacco use disorder complicating pregnancy, childbirth, or the puerperium, postpartum condition or complication |
| F1010 | 10 | Alcohol abuse, uncomplicated |
| F1011 | 10 | Alcohol abuse, in remission |
| F10120 | 10 | Alcohol abuse with intoxication, uncomplicated |
| F10121 | 10 | Alcohol abuse with intoxication delirium |
| F10129 | 10 | Alcohol abuse with intoxication, unspecified |
| F1014 | 10 | Alcohol abuse with alcohol-induced mood disorder |
| F10150 | 10 | Alcohol abuse with alcohol-induced psychotic disorder with delusions |
| F10151 | 10 | Alcohol abuse with alcohol-induced psychotic disorder with hallucinations |
| F10159 | 10 | Alcohol abuse with alcohol-induced psychotic disorder, unspecified |
| F10180 | 10 | Alcohol abuse with alcohol-induced anxiety disorder |
| F10181 | 10 | Alcohol abuse with alcohol-induced sexual dysfunction |
| F10182 | 10 | Alcohol abuse with alcohol-induced sleep disorder |
| F10188 | 10 | Alcohol abuse with other alcohol-induced disorder |
| F1019 | 10 | Alcohol abuse with unspecified alcohol-induced disorder |
| F1020 | 10 | Alcohol dependence, uncomplicated |
| F1021 | 10 | Alcohol dependence, in remission |
| F10220 | 10 | Alcohol dependence with intoxication, uncomplicated |
| F10221 | 10 | Alcohol dependence with intoxication delirium |
| F10229 | 10 | Alcohol dependence with intoxication, unspecified |
| F10230 | 10 | Alcohol dependence with withdrawal, uncomplicated |
| F10231 | 10 | Alcohol dependence with withdrawal delirium |
| F10232 | 10 | Alcohol dependence with withdrawal with perceptual disturbance |
| F10239 | 10 | Alcohol dependence with withdrawal, unspecified |
| F1024 | 10 | Alcohol dependence with alcohol-induced mood disorder |
| F10250 | 10 | Alcohol dependence with alcohol-induced psychotic disorder with delusions |
| F10251 | 10 | Alcohol dependence with alcohol-induced psychotic disorder with hallucinations |
| F10259 | 10 | Alcohol dependence with alcohol-induced psychotic disorder, unspecified |
| F1026 | 10 | Alcohol dependence with alcohol-induced persisting amnestic disorder |
| F1027 | 10 | Alcohol dependence with alcohol-induced persisting dementia |
| F10280 | 10 | Alcohol dependence with alcohol-induced anxiety disorder |
| F10281 | 10 | Alcohol dependence with alcohol-induced sexual dysfunction |
| F10282 | 10 | Alcohol dependence with alcohol-induced sleep disorder |
| F10288 | 10 | Alcohol dependence with other alcohol-induced disorder |
| F1029 | 10 | Alcohol dependence with unspecified alcohol-induced disorder |
| F10920 | 10 | Alcohol use, unspecified with intoxication, uncomplicated |
| F10921 | 10 | Alcohol use, unspecified with intoxication delirium |
| F10929 | 10 | Alcohol use, unspecified with intoxication, unspecified |
| F1094 | 10 | Alcohol use, unspecified with alcohol-induced mood disorder |
| F10950 | 10 | Alcohol use, unspecified with alcohol-induced psychotic disorder with delusions |
| F10951 | 10 | Alcohol use, unspecified with alcohol-induced psychotic disorder with hallucinations |
| F10959 | 10 | Alcohol use, unspecified with alcohol-induced psychotic disorder, unspecified |
| F1096 | 10 | Alcohol use, unspecified with alcohol-induced persisting amnestic disorder |
| F1097 | 10 | Alcohol use, unspecified with alcohol-induced persisting dementia |
| F10980 | 10 | Alcohol use, unspecified with alcohol-induced anxiety disorder |
| F10981 | 10 | Alcohol use, unspecified with alcohol-induced sexual dysfunction |
| F10982 | 10 | Alcohol use, unspecified with alcohol-induced sleep disorder |
| F10988 | 10 | Alcohol use, unspecified with other alcohol-induced disorder |
| F1099 | 10 | Alcohol use, unspecified with unspecified alcohol-induced disorder |
| F1110 | 10 | Opioid abuse, uncomplicated |
| F1111 | 10 | Opioid abuse, in remission |
| F11120 | 10 | Opioid abuse with intoxication, uncomplicated |
| F11121 | 10 | Opioid abuse with intoxication delirium |
| F11122 | 10 | Opioid abuse with intoxication with perceptual disturbance |
| F11129 | 10 | Opioid abuse with intoxication, unspecified |
| F1114 | 10 | Opioid abuse with opioid-induced mood disorder |
| F11150 | 10 | Opioid abuse with opioid-induced psychotic disorder with delusions |
| F11151 | 10 | Opioid abuse with opioid-induced psychotic disorder with hallucinations |
| F11159 | 10 | Opioid abuse with opioid-induced psychotic disorder, unspecified |
| F11181 | 10 | Opioid abuse with opioid-induced sexual dysfunction |
| F11182 | 10 | Opioid abuse with opioid-induced sleep disorder |
| F11188 | 10 | Opioid abuse with other opioid-induced disorder |
| F1119 | 10 | Opioid abuse with unspecified opioid-induced disorder |
| F1120 | 10 | Opioid dependence, uncomplicated |
| F1121 | 10 | Opioid dependence, in remission |
| F11220 | 10 | Opioid dependence with intoxication, uncomplicated |
| F11221 | 10 | Opioid dependence with intoxication delirium |
| F11222 | 10 | Opioid dependence with intoxication with perceptual disturbance |
| F11229 | 10 | Opioid dependence with intoxication, unspecified |
| F1123 | 10 | Opioid dependence with withdrawal |
| F1124 | 10 | Opioid dependence with opioid-induced mood disorder |
| F11250 | 10 | Opioid dependence with opioid-induced psychotic disorder with delusions |
| F11251 | 10 | Opioid dependence with opioid-induced psychotic disorder with hallucinations |
| F11259 | 10 | Opioid dependence with opioid-induced psychotic disorder, unspecified |
| F11281 | 10 | Opioid dependence with opioid-induced sexual dysfunction |
| F11282 | 10 | Opioid dependence with opioid-induced sleep disorder |
| F11288 | 10 | Opioid dependence with other opioid-induced disorder |
| F1129 | 10 | Opioid dependence with unspecified opioid-induced disorder |
| F1190 | 10 | Opioid use, unspecified, uncomplicated |
| F11920 | 10 | Opioid use, unspecified with intoxication, uncomplicated |
| F11921 | 10 | Opioid use, unspecified with intoxication delirium |
| F11922 | 10 | Opioid use, unspecified with intoxication with perceptual disturbance |
| F11929 | 10 | Opioid use, unspecified with intoxication, unspecified |
| F1193 | 10 | Opioid use, unspecified with withdrawal |
| F1194 | 10 | Opioid use, unspecified with opioid-induced mood disorder |
| F11950 | 10 | Opioid use, unspecified with opioid-induced psychotic disorder with delusions |
| F11951 | 10 | Opioid use, unspecified with opioid-induced psychotic disorder with hallucinations |
| F11959 | 10 | Opioid use, unspecified with opioid-induced psychotic disorder, unspecified |
| F11981 | 10 | Opioid use, unspecified with opioid-induced sexual dysfunction |
| F11982 | 10 | Opioid use, unspecified with opioid-induced sleep disorder |
| F11988 | 10 | Opioid use, unspecified with other opioid-induced disorder |
| F1199 | 10 | Opioid use, unspecified with unspecified opioid-induced disorder |
| F1210 | 10 | Cannabis abuse, uncomplicated |
| F1211 | 10 | Cannabis abuse, in remission |
| F12120 | 10 | Cannabis abuse with intoxication, uncomplicated |
| F12121 | 10 | Cannabis abuse with intoxication delirium |
| F12122 | 10 | Cannabis abuse with intoxication with perceptual disturbance |
| F12129 | 10 | Cannabis abuse with intoxication, unspecified |
| F12150 | 10 | Cannabis abuse with psychotic disorder with delusions |
| F12151 | 10 | Cannabis abuse with psychotic disorder with hallucinations |
| F12159 | 10 | Cannabis abuse with psychotic disorder, unspecified |
| F12180 | 10 | Cannabis abuse with cannabis-induced anxiety disorder |
| F12188 | 10 | Cannabis abuse with other cannabis-induced disorder |
| F1219 | 10 | Cannabis abuse with unspecified cannabis-induced disorder |
| F1220 | 10 | Cannabis dependence, uncomplicated |
| F1221 | 10 | Cannabis dependence, in remission |
| F12220 | 10 | Cannabis dependence with intoxication, uncomplicated |
| F12221 | 10 | Cannabis dependence with intoxication delirium |
| F12222 | 10 | Cannabis dependence with intoxication with perceptual disturbance |
| F12229 | 10 | Cannabis dependence with intoxication, unspecified |
| F1223 | 10 | Cannabis dependence with withdrawal |
| F12250 | 10 | Cannabis dependence with psychotic disorder with delusions |
| F12251 | 10 | Cannabis dependence with psychotic disorder with hallucinations |
| F12259 | 10 | Cannabis dependence with psychotic disorder, unspecified |
| F12280 | 10 | Cannabis dependence with cannabis-induced anxiety disorder |
| F12288 | 10 | Cannabis dependence with other cannabis-induced disorder |
| F1229 | 10 | Cannabis dependence with unspecified cannabis-induced disorder |
| F1290 | 10 | Cannabis use, unspecified, uncomplicated |
| F12920 | 10 | Cannabis use, unspecified with intoxication, uncomplicated |
| F12921 | 10 | Cannabis use, unspecified with intoxication delirium |
| F12922 | 10 | Cannabis use, unspecified with intoxication with perceptual disturbance |
| F12929 | 10 | Cannabis use, unspecified with intoxication, unspecified |
| F1293 | 10 | Cannabis use, unspecified with withdrawal |
| F12950 | 10 | Cannabis use, unspecified with psychotic disorder with delusions |
| F12951 | 10 | Cannabis use, unspecified with psychotic disorder with hallucinations |
| F12959 | 10 | Cannabis use, unspecified with psychotic disorder, unspecified |
| F12980 | 10 | Cannabis use, unspecified with anxiety disorder |
| F12988 | 10 | Cannabis use, unspecified with other cannabis-induced disorder |
| F1299 | 10 | Cannabis use, unspecified with unspecified cannabis-induced disorder |
| F1310 | 10 | Sedative, hypnotic or anxiolytic abuse, uncomplicated |
| F1311 | 10 | Sedative, hypnotic or anxiolytic abuse, in remission |
| F13120 | 10 | Sedative, hypnotic or anxiolytic abuse with intoxication, uncomplicated |
| F13121 | 10 | Sedative, hypnotic or anxiolytic abuse with intoxication delirium |
| F13129 | 10 | Sedative, hypnotic or anxiolytic abuse with intoxication, unspecified |
| F1314 | 10 | Sedative, hypnotic or anxiolytic abuse with sedative, hypnotic or anxiolytic-induced mood disorder |
| F13150 | 10 | Sedative, hypnotic or anxiolytic abuse with sedative, hypnotic or anxiolytic-induced psychotic disorder with delusions |
| F13151 | 10 | Sedative, hypnotic or anxiolytic abuse with sedative, hypnotic or anxiolytic-induced psychotic disorder with hallucinations |
| F13159 | 10 | Sedative, hypnotic or anxiolytic abuse with sedative, hypnotic or anxiolytic-induced psychotic disorder, unspecified |
| F13180 | 10 | Sedative, hypnotic or anxiolytic abuse with sedative, hypnotic or anxiolytic-induced anxiety disorder |
| F13181 | 10 | Sedative, hypnotic or anxiolytic abuse with sedative, hypnotic or anxiolytic-induced sexual dysfunction |
| F13182 | 10 | Sedative, hypnotic or anxiolytic abuse with sedative, hypnotic or anxiolytic-induced sleep disorder |
| F13188 | 10 | Sedative, hypnotic or anxiolytic abuse with other sedative, hypnotic or anxiolytic-induced disorder |
| F1319 | 10 | Sedative, hypnotic or anxiolytic abuse with unspecified sedative, hypnotic or anxiolytic-induced disorder |
| F1320 | 10 | Sedative, hypnotic or anxiolytic dependence, uncomplicated |
| F1321 | 10 | Sedative, hypnotic or anxiolytic dependence, in remission |
| F13220 | 10 | Sedative, hypnotic or anxiolytic dependence with intoxication, uncomplicated |
| F13221 | 10 | Sedative, hypnotic or anxiolytic dependence with intoxication delirium |
| F13229 | 10 | Sedative, hypnotic or anxiolytic dependence with intoxication, unspecified |
| F13230 | 10 | Sedative, hypnotic or anxiolytic dependence with withdrawal, uncomplicated |
| F13231 | 10 | Sedative, hypnotic or anxiolytic dependence with withdrawal delirium |
| F13232 | 10 | Sedative, hypnotic or anxiolytic dependence with withdrawal with perceptual disturbance |
| F13239 | 10 | Sedative, hypnotic or anxiolytic dependence with withdrawal, unspecified |
| F1324 | 10 | Sedative, hypnotic or anxiolytic dependence with sedative, hypnotic or anxiolytic-induced mood disorder |
| F13250 | 10 | Sedative, hypnotic or anxiolytic dependence with sedative, hypnotic or anxiolytic-induced psychotic disorder with delusions |
| F13251 | 10 | Sedative, hypnotic or anxiolytic dependence with sedative, hypnotic or anxiolytic-induced psychotic disorder with hallucinations |
| F13259 | 10 | Sedative, hypnotic or anxiolytic dependence with sedative, hypnotic or anxiolytic-induced psychotic disorder, unspecified |
| F1326 | 10 | Sedative, hypnotic or anxiolytic dependence with sedative, hypnotic or anxiolytic-induced persisting amnestic disorder |
| F1327 | 10 | Sedative, hypnotic or anxiolytic dependence with sedative, hypnotic or anxiolytic-induced persisting dementia |
| F13280 | 10 | Sedative, hypnotic or anxiolytic dependence with sedative, hypnotic or anxiolytic-induced anxiety disorder |
| F13281 | 10 | Sedative, hypnotic or anxiolytic dependence with sedative, hypnotic or anxiolytic-induced sexual dysfunction |
| F13282 | 10 | Sedative, hypnotic or anxiolytic dependence with sedative, hypnotic or anxiolytic-induced sleep disorder |
| F13288 | 10 | Sedative, hypnotic or anxiolytic dependence with other sedative, hypnotic or anxiolytic-induced disorder |
| F1329 | 10 | Sedative, hypnotic or anxiolytic dependence with unspecified sedative, hypnotic or anxiolytic-induced disorder |
| F1390 | 10 | Sedative, hypnotic, or anxiolytic use, unspecified, uncomplicated |
| F13920 | 10 | Sedative, hypnotic or anxiolytic use, unspecified with intoxication, uncomplicated |
| F13921 | 10 | Sedative, hypnotic or anxiolytic use, unspecified with intoxication delirium |
| F13929 | 10 | Sedative, hypnotic or anxiolytic use, unspecified with intoxication, unspecified |
| F13930 | 10 | Sedative, hypnotic or anxiolytic use, unspecified with withdrawal, uncomplicated |
| F13931 | 10 | Sedative, hypnotic or anxiolytic use, unspecified with withdrawal delirium |
| F13932 | 10 | Sedative, hypnotic or anxiolytic use, unspecified with withdrawal with perceptual disturbances |
| F15221 | 10 | Other stimulant dependence with intoxication delirium |
| F13939 | 10 | Sedative, hypnotic or anxiolytic use, unspecified with withdrawal, unspecified |
| F1394 | 10 | Sedative, hypnotic or anxiolytic use, unspecified with sedative, hypnotic or anxiolytic-induced mood disorder |
| F13950 | 10 | Sedative, hypnotic or anxiolytic use, unspecified with sedative, hypnotic or anxiolytic-induced psychotic disorder with delusions |
| F13951 | 10 | Sedative, hypnotic or anxiolytic use, unspecified with sedative, hypnotic or anxiolytic-induced psychotic disorder with hallucinations |
| F13959 | 10 | Sedative, hypnotic or anxiolytic use, unspecified with sedative, hypnotic or anxiolytic-induced psychotic disorder, unspecified |
| F1396 | 10 | Sedative, hypnotic or anxiolytic use, unspecified with sedative, hypnotic or anxiolytic-induced persisting amnestic disorder |
| F1397 | 10 | Sedative, hypnotic or anxiolytic use, unspecified with sedative, hypnotic or anxiolytic-induced persisting dementia |
| F13980 | 10 | Sedative, hypnotic or anxiolytic use, unspecified with sedative, hypnotic or anxiolytic-induced anxiety disorder |
| F13981 | 10 | Sedative, hypnotic or anxiolytic use, unspecified with sedative, hypnotic or anxiolytic-induced sexual dysfunction |
| F13982 | 10 | Sedative, hypnotic or anxiolytic use, unspecified with sedative, hypnotic or anxiolytic-induced sleep disorder |
| F13988 | 10 | Sedative, hypnotic or anxiolytic use, unspecified with other sedative, hypnotic or anxiolytic-induced disorder |
| F1399 | 10 | Sedative, hypnotic or anxiolytic use, unspecified with unspecified sedative, hypnotic or anxiolytic-induced disorder |
| F1410 | 10 | Cocaine abuse, uncomplicated |
| F1411 | 10 | Cocaine abuse, in remission |
| F14120 | 10 | Cocaine abuse with intoxication, uncomplicated |
| F14121 | 10 | Cocaine abuse with intoxication with delirium |
| F14122 | 10 | Cocaine abuse with intoxication with perceptual disturbance |
| F14129 | 10 | Cocaine abuse with intoxication, unspecified |
| F1414 | 10 | Cocaine abuse with cocaine-induced mood disorder |
| F14150 | 10 | Cocaine abuse with cocaine-induced psychotic disorder with delusions |
| F14151 | 10 | Cocaine abuse with cocaine-induced psychotic disorder with hallucinations |
| F14159 | 10 | Cocaine abuse with cocaine-induced psychotic disorder, unspecified |
| F14180 | 10 | Cocaine abuse with cocaine-induced anxiety disorder |
| F14181 | 10 | Cocaine abuse with cocaine-induced sexual dysfunction |
| F14182 | 10 | Cocaine abuse with cocaine-induced sleep disorder |
| F14188 | 10 | Cocaine abuse with other cocaine-induced disorder |
| F1419 | 10 | Cocaine abuse with unspecified cocaine-induced disorder |
| F1420 | 10 | Cocaine dependence, uncomplicated |
| F1421 | 10 | Cocaine dependence, in remission |
| F14220 | 10 | Cocaine dependence with intoxication, uncomplicated |
| F14221 | 10 | Cocaine dependence with intoxication delirium |
| F14222 | 10 | Cocaine dependence with intoxication with perceptual disturbance |
| F14229 | 10 | Cocaine dependence with intoxication, unspecified |
| F1423 | 10 | Cocaine dependence with withdrawal |
| F1424 | 10 | Cocaine dependence with cocaine-induced mood disorder |
| F14250 | 10 | Cocaine dependence with cocaine-induced psychotic disorder with delusions |
| F14251 | 10 | Cocaine dependence with cocaine-induced psychotic disorder with hallucinations |
| F14259 | 10 | Cocaine dependence with cocaine-induced psychotic disorder, unspecified |
| F14280 | 10 | Cocaine dependence with cocaine-induced anxiety disorder |
| F14281 | 10 | Cocaine dependence with cocaine-induced sexual dysfunction |
| F14282 | 10 | Cocaine dependence with cocaine-induced sleep disorder |
| F14288 | 10 | Cocaine dependence with other cocaine-induced disorder |
| F1429 | 10 | Cocaine dependence with unspecified cocaine-induced disorder |
| F1490 | 10 | Cocaine use, unspecified, uncomplicated |
| F14920 | 10 | Cocaine use, unspecified with intoxication, uncomplicated |
| F14921 | 10 | Cocaine use, unspecified with intoxication delirium |
| F14922 | 10 | Cocaine use, unspecified with intoxication with perceptual disturbance |
| F14929 | 10 | Cocaine use, unspecified with intoxication, unspecified |
| F1494 | 10 | Cocaine use, unspecified with cocaine-induced mood disorder |
| F14950 | 10 | Cocaine use, unspecified with cocaine-induced psychotic disorder with delusions |
| F14951 | 10 | Cocaine use, unspecified with cocaine-induced psychotic disorder with hallucinations |
| F14959 | 10 | Cocaine use, unspecified with cocaine-induced psychotic disorder, unspecified |
| F14980 | 10 | Cocaine use, unspecified with cocaine-induced anxiety disorder |
| F14981 | 10 | Cocaine use, unspecified with cocaine-induced sexual dysfunction |
| F14982 | 10 | Cocaine use, unspecified with cocaine-induced sleep disorder |
| F14988 | 10 | Cocaine use, unspecified with other cocaine-induced disorder |
| F1499 | 10 | Cocaine use, unspecified with unspecified cocaine-induced disorder |
| F1510 | 10 | Other stimulant abuse, uncomplicated |
| F1511 | 10 | Other stimulant abuse, in remission |
| F15120 | 10 | Other stimulant abuse with intoxication, uncomplicated |
| F15121 | 10 | Other stimulant abuse with intoxication delirium |
| F15122 | 10 | Other stimulant abuse with intoxication with perceptual disturbance |
| F15129 | 10 | Other stimulant abuse with intoxication, unspecified |
| F1514 | 10 | Other stimulant abuse with stimulant-induced mood disorder |
| F15150 | 10 | Other stimulant abuse with stimulant-induced psychotic disorder with delusions |
| F15151 | 10 | Other stimulant abuse with stimulant-induced psychotic disorder with hallucinations |
| F15159 | 10 | Other stimulant abuse with stimulant-induced psychotic disorder, unspecified |
| F15180 | 10 | Other stimulant abuse with stimulant-induced anxiety disorder |
| F15181 | 10 | Other stimulant abuse with stimulant-induced sexual dysfunction |
| F15182 | 10 | Other stimulant abuse with stimulant-induced sleep disorder |
| F15188 | 10 | Other stimulant abuse with other stimulant-induced disorder |
| F1519 | 10 | Other stimulant abuse with unspecified stimulant-induced disorder |
| F1520 | 10 | Other stimulant dependence, uncomplicated |
| F1521 | 10 | Other stimulant dependence, in remission |
| F15220 | 10 | Other stimulant dependence with intoxication, uncomplicated |
| F15222 | 10 | Other stimulant dependence with intoxication with perceptual disturbance |
| F15229 | 10 | Other stimulant dependence with intoxication, unspecified |
| F1523 | 10 | Other stimulant dependence with withdrawal |
| F1524 | 10 | Other stimulant dependence with stimulant-induced mood disorder |
| F15250 | 10 | Other stimulant dependence with stimulant-induced psychotic disorder with delusions |
| F15251 | 10 | Other stimulant dependence with stimulant-induced psychotic disorder with hallucinations |
| F15259 | 10 | Other stimulant dependence with stimulant-induced psychotic disorder, unspecified |
| F15280 | 10 | Other stimulant dependence with stimulant-induced anxiety disorder |
| F15281 | 10 | Other stimulant dependence with stimulant-induced sexual dysfunction |
| F15282 | 10 | Other stimulant dependence with stimulant-induced sleep disorder |
| F15288 | 10 | Other stimulant dependence with other stimulant-induced disorder |
| F1529 | 10 | Other stimulant dependence with unspecified stimulant-induced disorder |
| F1590 | 10 | Other stimulant use, unspecified, uncomplicated |
| F15920 | 10 | Other stimulant use, unspecified with intoxication, uncomplicated |
| F15921 | 10 | Other stimulant use, unspecified with intoxication delirium |
| F15922 | 10 | Other stimulant use, unspecified with intoxication with perceptual disturbance |
| F15929 | 10 | Other stimulant use, unspecified with intoxication, unspecified |
| F1593 | 10 | Other stimulant use, unspecified with withdrawal |
| F1594 | 10 | Other stimulant use, unspecified with stimulant-induced mood disorder |
| F15950 | 10 | Other stimulant use, unspecified with stimulant-induced psychotic disorder with delusions |
| F15951 | 10 | Other stimulant use, unspecified with stimulant-induced psychotic disorder with hallucinations |
| F15959 | 10 | Other stimulant use, unspecified with stimulant-induced psychotic disorder, unspecified |
| F15980 | 10 | Other stimulant use, unspecified with stimulant-induced anxiety disorder |
| F15981 | 10 | Other stimulant use, unspecified with stimulant-induced sexual dysfunction |
| F15982 | 10 | Other stimulant use, unspecified with stimulant-induced sleep disorder |
| F15988 | 10 | Other stimulant use, unspecified with other stimulant-induced disorder |
| F1599 | 10 | Other stimulant use, unspecified with unspecified stimulant-induced disorder |
| F1511 | 10 | Other stimulant abuse, in remission |
| F15120 | 10 | Other stimulant abuse with intoxication, uncomplicated |
| F15121 | 10 | Other stimulant abuse with intoxication delirium |
| F15122 | 10 | Other stimulant abuse with intoxication with perceptual disturbance |
| F15129 | 10 | Other stimulant abuse with intoxication, unspecified |
| F1514 | 10 | Other stimulant abuse with stimulant-induced mood disorder |
| F15150 | 10 | Other stimulant abuse with stimulant-induced psychotic disorder with delusions |
| F15151 | 10 | Other stimulant abuse with stimulant-induced psychotic disorder with hallucinations |
| F15159 | 10 | Other stimulant abuse with stimulant-induced psychotic disorder, unspecified |
| F15180 | 10 | Other stimulant abuse with stimulant-induced anxiety disorder |
| F15181 | 10 | Other stimulant abuse with stimulant-induced sexual dysfunction |
| F15182 | 10 | Other stimulant abuse with stimulant-induced sleep disorder |
| F15188 | 10 | Other stimulant abuse with other stimulant-induced disorder |
| F1519 | 10 | Other stimulant abuse with unspecified stimulant-induced disorder |
| F1520 | 10 | Other stimulant dependence, uncomplicated |
| F1521 | 10 | Other stimulant dependence, in remission |
| F15220 | 10 | Other stimulant dependence with intoxication, uncomplicated |
| F15222 | 10 | Other stimulant dependence with intoxication with perceptual disturbance |
| F15229 | 10 | Other stimulant dependence with intoxication, unspecified |
| F1523 | 10 | Other stimulant dependence with withdrawal |
| F1524 | 10 | Other stimulant dependence with stimulant-induced mood disorder |
| F15250 | 10 | Other stimulant dependence with stimulant-induced psychotic disorder with delusions |
| F15251 | 10 | Other stimulant dependence with stimulant-induced psychotic disorder with hallucinations |
| F15259 | 10 | Other stimulant dependence with stimulant-induced psychotic disorder, unspecified |
| F15280 | 10 | Other stimulant dependence with stimulant-induced anxiety disorder |
| F15281 | 10 | Other stimulant dependence with stimulant-induced sexual dysfunction |
| F15282 | 10 | Other stimulant dependence with stimulant-induced sleep disorder |
| F15288 | 10 | Other stimulant dependence with other stimulant-induced disorder |
| F1529 | 10 | Other stimulant dependence with unspecified stimulant-induced disorder |
| F1590 | 10 | Other stimulant use, unspecified, uncomplicated |
| F15920 | 10 | Other stimulant use, unspecified with intoxication, uncomplicated |
| F15921 | 10 | Other stimulant use, unspecified with intoxication delirium |
| F15922 | 10 | Other stimulant use, unspecified with intoxication with perceptual disturbance |
| F15929 | 10 | Other stimulant use, unspecified with intoxication, unspecified |
| F1593 | 10 | Other stimulant use, unspecified with withdrawal |
| F1594 | 10 | Other stimulant use, unspecified with stimulant-induced mood disorder |
| F15950 | 10 | Other stimulant use, unspecified with stimulant-induced psychotic disorder with delusions |
| F15951 | 10 | Other stimulant use, unspecified with stimulant-induced psychotic disorder with hallucinations |
| F15959 | 10 | Other stimulant use, unspecified with stimulant-induced psychotic disorder, unspecified |
| F15980 | 10 | Other stimulant use, unspecified with stimulant-induced anxiety disorder |
| F15981 | 10 | Other stimulant use, unspecified with stimulant-induced sexual dysfunction |
| F15982 | 10 | Other stimulant use, unspecified with stimulant-induced sleep disorder |
| F15988 | 10 | Other stimulant use, unspecified with other stimulant-induced disorder |
| F1599 | 10 | Other stimulant use, unspecified with unspecified stimulant-induced disorder |
| F1610 | 10 | Hallucinogen abuse, uncomplicated |
| F1611 | 10 | Hallucinogen abuse, in remission |
| F16120 | 10 | Hallucinogen abuse with intoxication, uncomplicated |
| F16121 | 10 | Hallucinogen abuse with intoxication with delirium |
| F16122 | 10 | Hallucinogen abuse with intoxication with perceptual disturbance |
| F16129 | 10 | Hallucinogen abuse with intoxication, unspecified |
| F1614 | 10 | Hallucinogen abuse with hallucinogen-induced mood disorder |
| F16150 | 10 | Hallucinogen abuse with hallucinogen-induced psychotic disorder with delusions |
| F16151 | 10 | Hallucinogen abuse with hallucinogen-induced psychotic disorder with hallucinations |
| F16159 | 10 | Hallucinogen abuse with hallucinogen-induced psychotic disorder, unspecified |
| F16180 | 10 | Hallucinogen abuse with hallucinogen-induced anxiety disorder |
| F16183 | 10 | Hallucinogen abuse with hallucinogen persisting perception disorder (flashbacks) |
| F16188 | 10 | Hallucinogen abuse with other hallucinogen-induced disorder |
| F1619 | 10 | Hallucinogen abuse with unspecified hallucinogen-induced disorder |
| F1620 | 10 | Hallucinogen dependence, uncomplicated |
| F1621 | 10 | Hallucinogen dependence, in remission |
| F16220 | 10 | Hallucinogen dependence with intoxication, uncomplicated |
| F16221 | 10 | Hallucinogen dependence with intoxication with delirium |
| F16229 | 10 | Hallucinogen dependence with intoxication, unspecified |
| F1624 | 10 | Hallucinogen dependence with hallucinogen-induced mood disorder |
| F16250 | 10 | Hallucinogen dependence with hallucinogen-induced psychotic disorder with delusions |
| F16251 | 10 | Hallucinogen dependence with hallucinogen-induced psychotic disorder with hallucinations |
| F16259 | 10 | Hallucinogen dependence with hallucinogen-induced psychotic disorder, unspecified |
| F16280 | 10 | Hallucinogen dependence with hallucinogen-induced anxiety disorder |
| F16283 | 10 | Hallucinogen dependence with hallucinogen persisting perception disorder (flashbacks) |
| F16288 | 10 | Hallucinogen dependence with other hallucinogen-induced disorder |
| F1629 | 10 | Hallucinogen dependence with unspecified hallucinogen-induced disorder |
| F1690 | 10 | Hallucinogen use, unspecified, uncomplicated |
| F16920 | 10 | Hallucinogen use, unspecified with intoxication, uncomplicated |
| F16921 | 10 | Hallucinogen use, unspecified with intoxication with delirium |
| F16929 | 10 | Hallucinogen use, unspecified with intoxication, unspecified |
| F1694 | 10 | Hallucinogen use, unspecified with hallucinogen-induced mood disorder |
| F16950 | 10 | Hallucinogen use, unspecified with hallucinogen-induced psychotic disorder with delusions |
| F16951 | 10 | Hallucinogen use, unspecified with hallucinogen-induced psychotic disorder with hallucinations |
| F16959 | 10 | Hallucinogen use, unspecified with hallucinogen-induced psychotic disorder, unspecified |
| F16980 | 10 | Hallucinogen use, unspecified with hallucinogen-induced anxiety disorder |
| F16983 | 10 | Hallucinogen use, unspecified with hallucinogen persisting perception disorder (flashbacks) |
| F16988 | 10 | Hallucinogen use, unspecified with other hallucinogen-induced disorder |
| F1699 | 10 | Hallucinogen use, unspecified with unspecified hallucinogen-induced disorder |
| F17200 | 10 | Nicotine dependence, unspecified, uncomplicated |
| F17201 | 10 | Nicotine dependence, unspecified, in remission |
| F17203 | 10 | Nicotine dependence unspecified, with withdrawal |
| F17208 | 10 | Nicotine dependence, unspecified, with other nicotine-induced disorders |
| F17209 | 10 | Nicotine dependence, unspecified, with unspecified nicotine-induced disorders |
| F17210 | 10 | Nicotine dependence, cigarettes, uncomplicated |
| F17211 | 10 | Nicotine dependence, cigarettes, in remission |
| F17213 | 10 | Nicotine dependence, cigarettes, with withdrawal |
| F17218 | 10 | Nicotine dependence, cigarettes, with other nicotine-induced disorders |
| F17219 | 10 | Nicotine dependence, cigarettes, with unspecified nicotine-induced disorders |
| F17220 | 10 | Nicotine dependence, chewing tobacco, uncomplicated |
| F17221 | 10 | Nicotine dependence, chewing tobacco, in remission |
| F17223 | 10 | Nicotine dependence, chewing tobacco, with withdrawal |
| F17228 | 10 | Nicotine dependence, chewing tobacco, with other nicotine-induced disorders |
| F17229 | 10 | Nicotine dependence, chewing tobacco, with unspecified nicotine-induced disorders |
| F17290 | 10 | Nicotine dependence, other tobacco product, uncomplicated |
| F17291 | 10 | Nicotine dependence, other tobacco product, in remission |
| F17293 | 10 | Nicotine dependence, other tobacco product, with withdrawal |
| F17298 | 10 | Nicotine dependence, other tobacco product, with other nicotine-induced disorders |
| F17299 | 10 | Nicotine dependence, other tobacco product, with unspecified nicotine-induced disorders |
| F1910 | 10 | Other psychoactive substance abuse, uncomplicated |
| F1911 | 10 | Other psychoactive substance abuse, in remission |
| F19120 | 10 | Other psychoactive substance abuse with intoxication, uncomplicated |
| F19121 | 10 | Other psychoactive substance abuse with intoxication delirium |
| F19122 | 10 | Other psychoactive substance abuse with intoxication with perceptual disturbances |
| F19129 | 10 | Other psychoactive substance abuse with intoxication, unspecified |
| F1914 | 10 | Other psychoactive substance abuse with psychoactive substance-induced mood disorder |
| F19150 | 10 | Other psychoactive substance abuse with psychoactive substance-induced psychotic disorder with delusions |
| F19151 | 10 | Other psychoactive substance abuse with psychoactive substance-induced psychotic disorder with hallucinations |
| F19159 | 10 | Other psychoactive substance abuse with psychoactive substance-induced psychotic disorder, unspecified |
| F1916 | 10 | Other psychoactive substance abuse with psychoactive substance-induced persisting amnestic disorder |
| F1917 | 10 | Other psychoactive substance abuse with psychoactive substance-induced persisting dementia |
| F19180 | 10 | Other psychoactive substance abuse with psychoactive substance-induced anxiety disorder |
| F19181 | 10 | Other psychoactive substance abuse with psychoactive substance-induced sexual dysfunction |
| F19182 | 10 | Other psychoactive substance abuse with psychoactive substance-induced sleep disorder |
| F19188 | 10 | Other psychoactive substance abuse with other psychoactive substance-induced disorder |
| F1919 | 10 | Other psychoactive substance abuse with unspecified psychoactive substance-induced disorder |
| F1920 | 10 | Other psychoactive substance dependence, uncomplicated |
| F1921 | 10 | Other psychoactive substance dependence, in remission |
| F19220 | 10 | Other psychoactive substance dependence with intoxication, uncomplicated |
| F19221 | 10 | Other psychoactive substance dependence with intoxication delirium |
| F19222 | 10 | Other psychoactive substance dependence with intoxication with perceptual disturbance |
| F19229 | 10 | Other psychoactive substance dependence with intoxication, unspecified |
| F19230 | 10 | Other psychoactive substance dependence with withdrawal, uncomplicated |
| F19231 | 10 | Other psychoactive substance dependence with withdrawal delirium |
| F19232 | 10 | Other psychoactive substance dependence with withdrawal with perceptual disturbance |
| F458 | 10 | Other somatoform disorders |
| F19239 | 10 | Other psychoactive substance dependence with withdrawal, unspecified |
| F1924 | 10 | Other psychoactive substance dependence with psychoactive substance-induced mood disorder |
| F19250 | 10 | Other psychoactive substance dependence with psychoactive substance-induced psychotic disorder with delusions |
| F19251 | 10 | Other psychoactive substance dependence with psychoactive substance-induced psychotic disorder with hallucinations |
| F19259 | 10 | Other psychoactive substance dependence with psychoactive substance-induced psychotic disorder, unspecified |
| F1926 | 10 | Other psychoactive substance dependence with psychoactive substance-induced persisting amnestic disorder |
| F1927 | 10 | Other psychoactive substance dependence with psychoactive substance-induced persisting dementia |
| F19280 | 10 | Other psychoactive substance dependence with psychoactive substance-induced anxiety disorder |
| F19281 | 10 | Other psychoactive substance dependence with psychoactive substance-induced sexual dysfunction |
| F19282 | 10 | Other psychoactive substance dependence with psychoactive substance-induced sleep disorder |
| F19288 | 10 | Other psychoactive substance dependence with other psychoactive substance-induced disorder |
| F1929 | 10 | Other psychoactive substance dependence with unspecified psychoactive substance-induced disorder |
| F1990 | 10 | Other psychoactive substance use, unspecified, uncomplicated |
| F19920 | 10 | Other psychoactive substance use, unspecified with intoxication, uncomplicated |
| F19921 | 10 | Other psychoactive substance use, unspecified with intoxication with delirium |
| F19922 | 10 | Other psychoactive substance use, unspecified with intoxication with perceptual disturbance |
| F19929 | 10 | Other psychoactive substance use, unspecified with intoxication, unspecified |
| F19930 | 10 | Other psychoactive substance use, unspecified with withdrawal, uncomplicated |
| F19931 | 10 | Other psychoactive substance use, unspecified with withdrawal delirium |
| F19932 | 10 | Other psychoactive substance use, unspecified with withdrawal with perceptual disturbance |
| F19939 | 10 | Other psychoactive substance use, unspecified with withdrawal, unspecified |
| F1994 | 10 | Other psychoactive substance use, unspecified with psychoactive substance-induced mood disorder |
| F19950 | 10 | Other psychoactive substance use, unspecified with psychoactive substance-induced psychotic disorder with delusions |
| F19951 | 10 | Other psychoactive substance use, unspecified with psychoactive substance-induced psychotic disorder with hallucinations |
| F19959 | 10 | Other psychoactive substance use, unspecified with psychoactive substance-induced psychotic disorder, unspecified |
| F1996 | 10 | Other psychoactive substance use, unspecified with psychoactive substance-induced persisting amnestic disorder |
| F1997 | 10 | Other psychoactive substance use, unspecified with psychoactive substance-induced persisting dementia |
| F19980 | 10 | Other psychoactive substance use, unspecified with psychoactive substance-induced anxiety disorder |
| F19981 | 10 | Other psychoactive substance use, unspecified with psychoactive substance-induced sexual dysfunction |
| F19982 | 10 | Other psychoactive substance use, unspecified with psychoactive substance-induced sleep disorder |
| F19988 | 10 | Other psychoactive substance use, unspecified with other psychoactive substance-induced disorder |
| F1999 | 10 | Other psychoactive substance use, unspecified with unspecified psychoactive substance-induced disorder |
