# Supplementary material for: Propofol provides a significant survival advantage in sepsis-associated encephalopathy: A retrospective cohort study investigating one-year all-cause mortality
Source: PLoS One. 2026 Feb 5;21(2):e0340371. doi: 10.1371/journal.pone.0340371 (PMC12875438; doi:10.1371/journal.pone.0340371)
Supplement: S8 Table — (DOCX) [file pone.0340371.s008.docx]

Supporting Information

# **S8 Table.** Exclude patients with metabolic encephalopathy, hepatic encephalopathy, hypertensive encephalopathy, diabetes with coma, disorders of urea cycle, hypernatremia, wrnicke's encephalopathy from the MIMIC-IV database according to ICD-codes

| ICD-code | ICD | Description |
| --- | --- | --- |
| 34831 | 9 | Metabolic encephalopathy |
| 5722 | 9 | Hepatic encephalopathy |
| 700 | 9 | Viral hepatitis A with hepatic coma |
| 7020 | 9 | Viral hepatitis B with hepatic coma, acute or unspecified, without mention of hepatitis delta |
| 7021 | 9 | Viral hepatitis B with hepatic coma, acute or unspecified, with hepatitis delta |
| 7022 | 9 | Chronic viral hepatitis B with hepatic coma without hepatitis delta |
| 7023 | 9 | Chronic viral hepatitis B with hepatic coma with hepatitis delta |
| 7041 | 9 | Acute hepatitis C with hepatic coma |
| 7042 | 9 | Hepatitis delta without mention of active hepatitis B disease with hepatic coma |
| 7043 | 9 | Hepatitis E with hepatic coma |
| 7044 | 9 | Chronic hepatitis C with hepatic coma |
| 7049 | 9 | Other specified viral hepatitis with hepatic coma |
| 7052 | 9 | Hepatitis delta without mention of active hepatitis B disease or hepatic coma |
| 706 | 9 | Unspecified viral hepatitis with hepatic coma |
| 7071 | 9 | Unspecified viral hepatitis C with hepatic coma |
| 2706 | 9 | Disorders of urea cycle metabolism |
| 2510 | 9 | Hypoglycemic coma |
| 4372 | 9 | Hypertensive encephalopathy |
| G9341 | 10 | Metabolic encephalopathy |
| B150 | 10 | Hepatitis A with hepatic coma |
| B159 | 10 | Hepatitis A without hepatic coma |
| B160 | 10 | Acute hepatitis B with delta-agent with hepatic coma |
| B161 | 10 | Acute hepatitis B with delta-agent without hepatic coma |
| B162 | 10 | Acute hepatitis B without delta-agent with hepatic coma |
| B169 | 10 | Acute hepatitis B without delta-agent and without hepatic coma |
| B170 | 10 | Acute delta-(super) infection of hepatitis B carrier |
| B1710 | 10 | Acute hepatitis C without hepatic coma |
| B1711 | 10 | Acute hepatitis C with hepatic coma |
| B190 | 10 | Unspecified viral hepatitis with hepatic coma |
| B1910 | 10 | Unspecified viral hepatitis B without hepatic coma |
| B1911 | 10 | Unspecified viral hepatitis B with hepatic coma |
| B1920 | 10 | Unspecified viral hepatitis C without hepatic coma |
| B1921 | 10 | Unspecified viral hepatitis C with hepatic coma |
| B199 | 10 | Unspecified viral hepatitis without hepatic coma |
| E7220 | 10 | Disorder of urea cycle metabolism, unspecified |
| E7229 | 10 | Other disorders of urea cycle metabolism |
| E15 | 10 | Nondiabetic hypoglycemic coma |
| E160 | 10 | Drug-induced hypoglycemia without coma |
| E161 | 10 | Other hypoglycemia |
| E162 | 10 | Hypoglycemia, unspecified |
| E512 | 10 | Wernicke's encephalopathy |
| G92 | 10 | Toxic encephalopathy |
| G9341 | 10 | Metabolic encephalopathy |
| I674 | 10 | Hypertensive encephalopathy |
| P9160 | 10 | Hypoxic ischemic encephalopathy [HIE], unspecified |
| P9161 | 10 | Mild hypoxic ischemic encephalopathy [HIE] |
| P9162 | 10 | Moderate hypoxic ischemic encephalopathy [HIE] |
| P9163 | 10 | Severe hypoxic ischemic encephalopathy [HIE] |
