# Supplementary material for: Propofol provides a significant survival advantage in sepsis-associated encephalopathy: A retrospective cohort study investigating one-year all-cause mortality
Source: PLoS One. 2026 Feb 5;21(2):e0340371. doi: 10.1371/journal.pone.0340371 (PMC12875438; doi:10.1371/journal.pone.0340371)
Supplement: S9 Table — (DOCX) [file pone.0340371.s009.docx]

Supporting Information

**S9 Table. Assessing proportional hazards**

| Sedatives | Chi-square | df | *P*-value |
| --- | --- | --- | --- |
| Dexmedetomidine | 8.589 | 1.0 | 0.003 |
| Propofol+Midazolam+Dexmedetomidine | 3.666 | 1.0 | 0.056 |
