# Supplementary material for: Propofol provides a significant survival advantage in sepsis-associated encephalopathy: A retrospective cohort study investigating one-year all-cause mortality
Source: PLoS One. 2026 Feb 5;21(2):e0340371. doi: 10.1371/journal.pone.0340371 (PMC12875438; doi:10.1371/journal.pone.0340371)
Supplement: S10 Table — (DOCX) [file pone.0340371.s010.docx]

Supporting Information

**S10 Table. Comparing restricted mean survival time of Dexmedetomidine with Non-sedative.**

| Restricted mean survival time, days | Estimate with 95%CI | p |
| --- | --- | --- |
| Dexmedetomidine - (Non-sedative) | 68.678 (4.692~132.664) | 0.035 |
| Dexmedetomidine / (Non-sedative) | 1.307 (1.048~1.628) | 0.017 |
