# Supplementary material for: Propofol provides a significant survival advantage in sepsis-associated encephalopathy: A retrospective cohort study investigating one-year all-cause mortality
Source: PLoS One. 2026 Feb 5;21(2):e0340371. doi: 10.1371/journal.pone.0340371 (PMC12875438; doi:10.1371/journal.pone.0340371)
Supplement: S11 Table — (DOCX) [file pone.0340371.s011.docx]

Supporting Information

**S11 Table. Vasopressor and Sedative in the Original and Matched Cohorts.**

| **Variables** | **Original cohort** | | | | **Matched cohort** | | | |
| --- | --- | --- | --- | --- | --- | --- | --- | --- |
|  | **Total (n = 4618)** | **Sedative use (n=3343)** | **Non-sedative use (n=1275)** | ***P*-value** | **Total (n = 1022)** | **Sedative use (n=511)** | **Non-sedative use (n=511)** | ***P*-value** |
| Receiving ≥2 types of vasoactive agents, n | 782 (16.9) | 692 (20.7) | 90 (7.1) | < 0.001 | 101 ( 9.9) | 49 (9.6) | 52 (10.2) | 0.753 |
| Norepinephrine equivalent dose, μg | 164.7 (0.0, 4272.6) | 686.7 (0.0, 5541.7) | 0.0 (0.0, 189.7) | < 0.001 | 0.0 (0.0, 2304.8) | 0.0 (0.0, 2207.1) | 0.0 (0.0, 2778.1) | 0.917 |
| Total dose of propofol, mg | 1000.0 (0.0, 1200.0) | 1000.0 (1000.0, 1200.0) | 0.0 (0.0, 0.0) | < 0.001 | 0.0 (0.0, 0.0) | 0.0 (0.0, 25.0) | 0.0 (0.0, 0.0) | < 0.001 |
| Duration of propofol, hrs | 9.0 (0.0, 20.5) | 17.2 (7.0, 21.9) | 0.0 (0.0, 0.0) | < 0.001 | 0.0 (0.0, 14.6) | 13.0 (0.9, 21.4) | 0.0 (0.0, 0.0) | < 0.001 |
| Total dose of midazolam, mg | 0.0 (0.0, 0.0) | 0.0 (0.0, 0.5) | 0.0 (0.0, 0.0) | < 0.001 | 0.0 (0.0, 0.0) | 0.0 (0.0, 1.0) | 0.0 (0.0, 0.0) | < 0.001 |
| Duration of midazolam, hrs | 0.0 (0.0, 0.0) | 0.0 (0.0, 0.0) | 0.0 (0.0, 0.0) | < 0.001 | 0.0 (0.0, 0.0) | 0.0 (0.0, 7.8) | 0.0 (0.0, 0.0) | < 0.001 |
| Total dose of dexmedetomidine, mg | 0.0 (0.0, 0.0) | 0.0 (0.0, 0.0) | 0.0 (0.0, 0.0) | < 0.001 | 0.0 (0.0, 0.0) | 0.0 (0.0, 0.0) | 0.0 (0.0, 0.0) | 0.025 |
| Duration of dexmedetomidine, hrs | 0.0 (0.0, 0.0) | 0.0 (0.0, 0.0) | 0.0 (0.0, 0.0) | < 0.001 | 0.0 (0.0, 0.0) | 0.0 (0.0, 0.0) | 0.0 (0.0, 0.0) | < 0.001 |
| Duration of sedation, hrs | 15.7 (0.0, 21.6) | 19.9 (12.8, 22.3) | 0.0 (0.0, 0.0) | < 0.001 | 1.4 (0.0, 19.0) | 18.1 (10.6, 22.0) | 0.0 (0.0, 0.0) | < 0.001 |

**Notes:** Data are presented as mean±SD, median (Q1–Q3), or N (%). Norepinephrine equivalent doses were derived from standard conversion formulas incorporating all vasopressor agents, following the approach described by Goradia et al. (2020), Vasopressor dose equivalence: A scoping review and suggested formula. Sedative doses indicate the total amount administered during the first 24 hours after ICU admission. Sedation durations indicate the total sedation hours during the first 24 hours after ICU admission.
