# Supplementary material for: Propofol provides a significant survival advantage in sepsis-associated encephalopathy: A retrospective cohort study investigating one-year all-cause mortality
Source: PLoS One. 2026 Feb 5;21(2):e0340371. doi: 10.1371/journal.pone.0340371 (PMC12875438; doi:10.1371/journal.pone.0340371)
Supplement: S12 Table — (DOCX) [file pone.0340371.s012.docx]

Supporting Information

**S12 Table. Baseline characteristics stratified by day-1 ventilation status in the original and matched cohorts.**

| **Variables** | **Original cohort** | | | | | | | |
| --- | --- | --- | --- | --- | --- | --- | --- | --- |
|  | **Ventilation=No** | | | | **Ventilation=Yes** | | | |
|  | **Total (n = 1991)** | **Non-sedative use (n=1002)** | **Sedative use (n=989)** | ***P*-value** | **Total (n = 2627)** | **Non-sedative use (n=273)** | **Sedative use (n=2354)** | ***P*-value** |
| **Demographic characteristics** |  |  |  |  |  |  |  |  |
| Male | 1184 (59.5) | 519 (51.8) | 665 (67.2) | < 0.001 | 1578 (60.1) | 160 (58.6) | 1418 (60.2) | 0.603 |
| Age, years | 66.6 ± 14.7 | 67.7 ± 14.9 | 65.6 ± 14.5 | 0.002 | 66.8 ± 14.8 | 69.3 ± 13.7 | 66.6 ± 14.9 | 0.003 |
| White | 1431 (71.9) | 719 (71.8) | 712 (72) | 0.907 | 1845 (70.2) | 190 (69.6) | 1655 (70.3) | 0.808 |
| **Vital signs, Mean ± SD** |  |  |  |  |  |  |  |  |
| Heart rate, beats per minute | 87.7 ± 15.6 | 90.7 ± 16.9 | 84.6 ± 13.4 | < 0.001 | 86.1 ± 14.9 | 88.6 ± 16.4 | 85.8 ± 14.6 | 0.003 |
| Systolic blood pressure, mmHg | 113.9 ± 14.5 | 114.3 ± 17.2 | 113.5 ± 11.2 | 0.193 | 113.1 ± 11.7 | 115.0 ± 14.4 | 112.9 ± 11.3 | 0.005 |
| Diastolic blood pressure, mmHg | 60.0 ± 9.7 | 61.0 ± 10.9 | 58.9 ± 8.1 | < 0.001 | 58.9 ± 8.9 | 61.3 ± 10.9 | 58.6 ± 8.6 | < 0.001 |
| Mean blood pressure, mmHg | 75.6 ± 9.6 | 75.7 ± 11.2 | 75.4 ± 7.6 | 0.491 | 75.2 ± 8.3 | 75.8 ± 10.2 | 75.1 ± 8.0 | 0.213 |
| Respiratory rate, breaths per minute | 19.4 ± 4.3 | 20.5 ± 4.7 | 18.3 ± 3.4 | < 0.001 | 19.0 ± 3.9 | 21.8 ± 4.8 | 18.7 ± 3.6 | < 0.001 |
| Body temperature, ℃ | 36.9 ± 0.5 | 36.8 ± 0.5 | 36.9 ± 0.6 | < 0.001 | 36.9 ± 0.6 | 36.9 ± 0.5 | 36.9 ± 0.6 | 0.933 |
| SpO_2_, % | 97.0 ± 1.9 | 96.4 ± 2.0 | 97.7 ± 1.5 | < 0.001 | 97.5 ± 1.9 | 95.8 ± 2.5 | 97.7 ± 1.7 | < 0.001 |
| **Laboratory tests** |  |  |  |  |  |  |  |  |
| BUN, mg/dL | 21.0 (15.0, 36.0) | 26.0 (17.0, 46.0) | 18.0 (14.0, 25.0) | < 0.001 | 20.0 (15.0, 33.0) | 28.5 (18.0, 49.0) | 20.0 (14.0, 31.0) | < 0.001 |
| Creatinine, mg/dL | 1.0 (0.8, 1.6) | 1.2 (0.8, 2.1) | 1.0 (0.8, 1.2) | < 0.001 | 1.0 (0.8, 1.6) | 1.2 (0.8, 2.0) | 1.0 (0.8, 1.6) | 0.015 |
| Glucose, mg/dL | 129.8 ± 25.7 | 132.6 ± 25.7 | 126.9 ± 25.3 | < 0.001 | 129.5 ± 25.5 | 129.9 ± 25.9 | 129.4 ± 25.5 | 0.759 |
| PaO_2_, mmHg | 107.2 ± 57.9 | 100.2 ± 59.8 | 114.3 ± 55.0 | < 0.001 | 99.2 ± 50.2 | 80.2 ± 45.8 | 101.4 ± 50.2 | < 0.001 |
| PaCO_2_, mmHg | 44.3 ± 9.9 | 41.6 ± 10.9 | 47.0 ± 8.0 | < 0.001 | 48.6 ± 9.3 | 47.2 ± 11.9 | 48.8 ± 9.0 | 0.01 |
| Lactate, mmol/L | 2.0 (1.4, 2.8) | 1.6 (1.2, 2.5) | 2.2 (1.6, 3.1) | < 0.001 | 2.4 (1.6, 3.6) | 1.6 (1.1, 2.6) | 2.5 (1.7, 3.7) | < 0.001 |
| Sodium, mmol/L | 136.6 ± 4.2 | 136.1 ± 4.9 | 137.0 ± 3.2 | < 0.001 | 137.1 ± 3.9 | 136.4 ± 5.0 | 137.2 ± 3.7 | < 0.001 |
| Potassium, mmol/L | 4.6 ± 0.7 | 4.6 ± 0.9 | 4.5 ± 0.6 | 0.131 | 4.6 ± 0.8 | 4.6 ± 0.9 | 4.6 ± 0.8 | 0.21 |
| Hemoglobin, g/dL | 9.7 ± 1.9 | 9.7 ± 2.0 | 9.6 ± 1.8 | 0.355 | 9.3 ± 1.9 | 9.5 ± 2.0 | 9.3 ± 1.8 | 0.092 |
| Platelets, x10^9^/L | 187.0 ± 107.7 | 203.1 ± 115.2 | 170.7 ± 97.1 | < 0.001 | 169.0 ± 101.6 | 219.5 ± 133.7 | 163.1 ± 95.5 | < 0.001 |
| WBC, x10^9^/L | 15.3 ± 9.9 | 14.8 ± 9.4 | 15.8 ± 10.3 | 0.019 | 15.7 ± 7.9 | 14.1 ± 7.9 | 15.9 ± 7.9 | < 0.001 |
| INR | 1.6 ± 1.0 | 1.7 ± 1.1 | 1.5 ± 0.9 | < 0.001 | 1.6 ± 1.0 | 2.0 ± 1.7 | 1.6 ± 0.9 | < 0.001 |
| PT, seconds | 17.6 ± 9.7 | 18.8 ± 11.4 | 16.5 ± 7.7 | < 0.001 | 18.0 ± 10.5 | 20.9 ± 16.5 | 17.7 ± 9.6 | < 0.001 |
| PTT, seconds | 42.3 ± 26.9 | 45.1 ± 30.1 | 39.7 ± 23.2 | < 0.001 | 45.5 ± 28.9 | 46.6 ± 32.0 | 45.4 ± 28.6 | 0.534 |
| **Site of infection, n (%)** |  |  |  |  |  |  |  |  |
| Intestinal infection | 24 ( 1.2) | 15 (1.5) | 9 (0.9) | 0.23 | 45 ( 1.7) | 2 (0.7) | 43 (1.8) | 0.318 |
| Catheter infection | 38 ( 1.9) | 28 (2.8) | 10 (1) | 0.004 | 37 ( 1.4) | 5 (1.8) | 32 (1.4) | 0.582 |
| Skin and soft tissue infection | 3 ( 0.2) | 2 (0.2) | 1 (0.1) | 1 | 1 ( 0.0) | 1 (0.4) | 0 (0) | 0.104 |
| Urinary infection | 219 (11.0) | 140 (14) | 79 (8) | < 0.001 | 271 (10.3) | 43 (15.8) | 228 (9.7) | 0.002 |
| Pulmonary infection | 374 (18.8) | 218 (21.8) | 156 (15.8) | < 0.001 | 516 (19.6) | 83 (30.4) | 433 (18.4) | < 0.001 |
| **Scores** |  |  |  |  |  |  |  |  |
| Charlson comorbidity index | 5.7 ± 2.9 | 6.5 ± 3.0 | 4.9 ± 2.5 | < 0.001 | 5.6 ± 2.7 | 6.6 ± 2.8 | 5.4 ± 2.6 | < 0.001 |
| SOFA | 3.6 ± 1.9 | 3.4 ± 1.8 | 3.8 ± 1.9 | < 0.001 | 4.1 ± 2.2 | 3.6 ± 2.0 | 4.1 ± 2.2 | < 0.001 |
| SAPSII | 37.5 ± 13.4 | 38.1 ± 12.7 | 36.9 ± 14.0 | 0.045 | 44.2 ± 14.0 | 40.6 ± 13.7 | 44.7 ± 14.0 | < 0.001 |
| **Treatments, n (%)** |  |  |  |  |  |  |  |  |
| First day vasopressor | 893 (44.9) | 266 (26.5) | 627 (63.4) | < 0.001 | 1694 (64.5) | 80 (29.3) | 1614 (68.6) | < 0.001 |
| First day renal replacement therapy | 99 ( 5.0) | 71 (7.1) | 28 (2.8) | < 0.001 | 146 ( 5.6) | 27 (9.9) | 119 (5.1) | < 0.001 |

| **Variables** | **Matched cohort** | | | | | | | |
| --- | --- | --- | --- | --- | --- | --- | --- | --- |
|  | **Ventilation=No** | | | | **Ventilation=Yes** | | | |
|  | **Total (n = 649)** | **Non-sedative use (n=342)** | **Sedative use (n=307)** | ***P*-value** | **Total (n = 373)** | **Non-sedative use (n=169)** | **Sedative use (n=204)** | ***P*-value** |
| **Demographic characteristics** |  |  |  |  |  |  |  |  |
| Male | 373 (57.5) | 193 (56.4) | 180 (58.6) | 0.572 | 200 (53.6) | 96 (56.8) | 104 (51) | 0.262 |
| Age, years | 65.9 ± 14.7 | 65.6 ± 15.0 | 66.1 ± 14.3 | 0.654 | 68.6 ± 15.0 | 68.9 ± 14.4 | 68.4 ± 15.6 | 0.761 |
| White | 456 (70.3) | 240 (70.2) | 216 (70.4) | 0.959 | 264 (70.8) | 121 (71.6) | 143 (70.1) | 0.751 |
| **Vital signs, Mean ± SD** |  |  |  |  |  |  |  |  |
| Heart rate, beats per minute | 87.0 ± 15.5 | 87.6 ± 16.2 | 86.4 ± 14.7 | 0.318 | 89.1 ± 17.0 | 87.5 ± 16.1 | 90.3 ± 17.6 | 0.111 |
| Systolic blood pressure, mmHg | 114.7 ± 15.1 | 115.0 ± 16.8 | 114.3 ± 12.9 | 0.579 | 114.0 ± 13.7 | 113.6 ± 13.1 | 114.3 ± 14.3 | 0.621 |
| Diastolic blood pressure, mmHg | 59.8 ± 9.7 | 60.0 ± 10.4 | 59.6 ± 9.0 | 0.635 | 60.5 ± 10.0 | 59.5 ± 10.3 | 61.4 ± 9.7 | 0.073 |
| Mean blood pressure, mmHg | 75.9 ± 10.0 | 76.1 ± 11.1 | 75.7 ± 8.6 | 0.567 | 75.5 ± 9.6 | 74.7 ± 9.7 | 76.2 ± 9.5 | 0.133 |
| Respiratory rate, breaths per minute | 18.9 ± 3.9 | 18.8 ± 3.9 | 19.0 ± 3.9 | 0.49 | 20.7 ± 4.1 | 20.8 ± 4.3 | 20.6 ± 4.0 | 0.762 |
| Body temperature, ℃ | 36.9 ± 0.5 | 36.9 ± 0.5 | 36.9 ± 0.6 | 0.77 | 36.9 ± 0.6 | 36.9 ± 0.5 | 37.0 ± 0.6 | 0.648 |
| SpO_2_, % | 97.2 ± 1.7 | 97.1 ± 1.6 | 97.2 ± 1.9 | 0.906 | 96.6 ± 2.3 | 96.6 ± 2.1 | 96.6 ± 2.5 | 0.875 |
| **Laboratory tests** |  |  |  |  |  |  |  |  |
| BUN, mg/dL | 21.0 (15.0, 36.0) | 21.0 (14.0, 38.8) | 20.0 (15.0, 31.0) | 0.41 | 26.0 (17.0, 48.0) | 29.0 (17.0, 48.0) | 25.5 (17.0, 48.0) | 0.61 |
| Creatinine, mg/dL | 1.0 (0.8, 1.6) | 1.1 (0.8, 1.8) | 1.0 (0.8, 1.5) | 0.194 | 1.2 (0.8, 2.1) | 1.2 (0.8, 1.9) | 1.1 (0.7, 2.2) | 0.784 |
| Glucose, mg/dL | 134.1 ± 25.1 | 135.5 ± 24.3 | 132.4 ± 26.0 | 0.114 | 133.1 ± 26.0 | 129.9 ± 25.9 | 135.8 ± 25.8 | 0.029 |
| PaO_2_, mmHg | 114.7 ± 70.5 | 118.3 ± 71.5 | 110.7 ± 69.2 | 0.17 | 95.0 ± 58.9 | 87.7 ± 48.6 | 101.2 ± 65.7 | 0.027 |
| PaCO_2_, mmHg | 44.1 ± 10.0 | 43.3 ± 10.8 | 45.0 ± 9.1 | 0.038 | 47.5 ± 11.3 | 49.5 ± 12.3 | 46.0 ± 10.2 | 0.003 |
| Lactate, mmol/L | 1.8 (1.3, 2.7) | 1.8 (1.2, 2.7) | 1.9 (1.3, 2.7) | 0.383 | 1.7 (1.2, 2.6) | 1.7 (1.2, 2.6) | 1.6 (1.2, 2.4) | 0.554 |
| Sodium, mmol/L | 136.8 ± 4.0 | 136.7 ± 4.0 | 136.9 ± 4.0 | 0.386 | 136.6 ± 4.9 | 136.4 ± 5.1 | 136.7 ± 4.7 | 0.612 |
| Potassium, mmol/L | 4.5 ± 0.7 | 4.5 ± 0.7 | 4.5 ± 0.6 | 0.837 | 4.6 ± 0.8 | 4.7 ± 0.8 | 4.6 ± 0.8 | 0.589 |
| Hemoglobin, g/dL | 9.7 ± 2.0 | 9.7 ± 2.0 | 9.6 ± 1.9 | 0.395 | 9.4 ± 1.9 | 9.2 ± 1.9 | 9.5 ± 1.9 | 0.163 |
| Platelets, x10^9^/L | 195.0 ± 117.1 | 194.5 ± 111.4 | 195.6 ± 123.3 | 0.903 | 203.6 ± 120.7 | 204.8 ± 121.0 | 202.7 ± 120.7 | 0.862 |
| WBC, x10^9^/L | 14.0 (9.9, 18.4) | 14.2 (9.5, 19.1) | 13.9 (10.2, 17.6) | 0.93 | 15.2 ± 9.9 | 14.6 ± 8.5 | 15.7 ± 10.9 | 0.328 |
| INR | 1.6 ± 1.0 | 1.5 ± 0.6 | 1.6 ± 1.2 | 0.371 | 1.4 (1.2, 1.8) | 1.4 (1.2, 1.9) | 1.4 (1.2, 1.7) | 0.801 |
| PT, seconds | 16.9 ± 8.7 | 16.6 ± 6.1 | 17.2 ± 10.7 | 0.365 | 15.2 (13.4, 19.7) | 15.1 (13.3, 20.9) | 15.2 (13.6, 19.0) | 0.754 |
| PTT, seconds | 32.7 (28.1, 42.3) | 32.7 (28.1, 43.0) | 32.7 (28.0, 42.1) | 0.773 | 34.5 (29.0, 45.5) | 35.1 (29.7, 48.3) | 33.6 (28.7, 43.4) | 0.141 |
| **Site of infection, n (%)** |  |  |  |  |  |  |  |  |
| Intestinal infection | 10 ( 1.5) | 6 (1.8) | 4 (1.3) | 0.756 | 5 ( 1.3) | 2 (1.2) | 3 (1.5) | 1 |
| Catheter infection | 15 ( 2.3) | 8 (2.3) | 7 (2.3) | 0.96 | 3 ( 0.8) | 2 (1.2) | 1 (0.5) | 0.592 |
| Skin and soft tissue infection | 1 ( 0.2) | 0 (0) | 1 (0.3) | 0.473 | 1 ( 0.3) | 1 (0.6) | 0 (0) | 0.453 |
| Urinary infection | 65 (10.0) | 34 (9.9) | 31 (10.1) | 0.947 | 52 (13.9) | 21 (12.4) | 31 (15.2) | 0.442 |
| Pulmonary infection | 114 (17.6) | 51 (14.9) | 63 (20.5) | 0.061 | 112 (30.0) | 55 (32.5) | 57 (27.9) | 0.334 |
| **Scores** |  |  |  |  |  |  |  |  |
| Charlson comorbidity index | 5.7 ± 2.9 | 5.8 ± 3.0 | 5.5 ± 2.7 | 0.316 | 6.4 ± 2.9 | 6.1 ± 2.7 | 6.6 ± 3.0 | 0.093 |
| SOFA | 3.5 ± 1.9 | 3.4 ± 1.8 | 3.7 ± 1.9 | 0.138 | 3.8 ± 2.0 | 3.9 ± 2.2 | 3.6 ± 1.8 | 0.098 |
| SAPSII | 36.4 ± 13.1 | 36.7 ± 12.8 | 36.1 ± 13.4 | 0.57 | 44.7 ± 14.4 | 43.0 ± 14.2 | 46.0 ± 14.4 | 0.042 |
| **Treatments, n (%)** |  |  |  |  |  |  |  |  |
| First day vasopressor | 248 (38.2) | 126 (36.8) | 122 (39.7) | 0.448 | 152 (40.8) | 72 (42.6) | 80 (39.2) | 0.507 |
| First day renal replacement therapy | 37 ( 5.7) | 21 (6.1) | 16 (5.2) | 0.61 | 39 (10.5) | 19 (11.2) | 20 (9.8) | 0.651 |

**Notes:** Data are presented as mean ± SD, median (Q1–Q3), or n (%), as appropriate. Ventilation indicates high-flow nasal cannula (HFNC), non-invasive ventilation (NIV), invasive mechanical ventilation (IMV), or tracheostomy ventilation on the first ICU day; RRT indicates renal replacement therapy initiated within the first 24 hours after ICU admission. Illness severity was assessed using SOFA and SAPS II (SAPS 3 not available in MIMIC-IV). Group comparisons used t-tests or Wilcoxon tests for continuous variables and χ²/Fisher’s exact tests for categorical variables.
