# Supplementary material for: Propofol provides a significant survival advantage in sepsis-associated encephalopathy: A retrospective cohort study investigating one-year all-cause mortality
Source: PLoS One. 2026 Feb 5;21(2):e0340371. doi: 10.1371/journal.pone.0340371 (PMC12875438; doi:10.1371/journal.pone.0340371)
Supplement: S13 Table — (DOCX) [file pone.0340371.s013.docx]

Supporting Information

**S13 Table. Baseline characteristics stratified by renal replacement therapy (RRT) within the first 24 hours in the original and matched cohorts.**

| **Variables** | **Original cohort** | | | | | | | |
| --- | --- | --- | --- | --- | --- | --- | --- | --- |
|  | **RRT=No** | | | | **RRT=Yes** | | | |
|  | **Total (n = 4373)** | **Non-sedative use (n=1177)** | **Sedative use (n=3196)** | ***P*-value** | **Total (n = 245)** | **Non-sedative use (n=98)** | **Sedative use (n=147)** | ***P*-value** |
| **Demographic characteristics** |  |  |  |  |  |  |  |  |
| Male | 2617 (59.9) | 625 (53.1) | 1992 (62.3) | < 0.001 | 145 (58.9) | 54 (55.1) | 91 (61.5) | 0.319 |
| Age, years | 66.9 ± 14.7 | 68.0 ± 14.7 | 66.5 ± 14.7 | 0.004 | 64.1 ± 15.3 | 68.7 ± 13.6 | 61.0 ± 15.6 | < 0.001 |
| White | 3141 (71.8) | 852 (72.4) | 2289 (71.6) | 0.627 | 135 (54.9) | 57 (58.2) | 78 (52.7) | 0.399 |
| **Vital signs, Mean ± SD** |  |  |  |  |  |  |  |  |
| Heart rate, beats per minute | 86.7 ± 15.1 | 90.4 ± 16.9 | 85.3 ± 14.2 | < 0.001 | 88.1 ± 16.4 | 88.3 ± 15.6 | 88.0 ± 16.9 | 0.887 |
| Systolic blood pressure, mmHg | 113.5 ± 12.7 | 114.7 ± 16.3 | 113.1 ± 11.1 | < 0.001 | 113.0 ± 17.3 | 112.3 ± 19.6 | 113.5 ± 15.5 | 0.59 |
| Diastolic blood pressure, mmHg | 59.4 ± 9.1 | 61.3 ± 10.7 | 58.7 ± 8.4 | < 0.001 | 58.2 ± 11.4 | 58.4 ± 13.1 | 58.1 ± 10.0 | 0.838 |
| Mean blood pressure, mmHg | 75.4 ± 8.7 | 75.9 ± 10.8 | 75.3 ± 7.8 | 0.025 | 73.8 ± 11.6 | 73.5 ± 13.1 | 74.0 ± 10.5 | 0.75 |
| Respiratory rate, breaths per minute | 19.1 ± 4.0 | 20.9 ± 4.8 | 18.5 ± 3.5 | < 0.001 | 20.2 ± 4.1 | 20.0 ± 3.7 | 20.3 ± 4.3 | 0.619 |
| Body temperature, ℃ | 36.9 ± 0.6 | 36.8 ± 0.5 | 36.9 ± 0.6 | < 0.001 | 36.8 ± 0.6 | 36.7 ± 0.5 | 36.8 ± 0.7 | 0.619 |
| SpO_2_, % | 97.3 ± 1.9 | 96.3 ± 2.1 | 97.7 ± 1.6 | < 0.001 | 97.0 ± 2.7 | 96.3 ± 2.7 | 97.4 ± 2.6 | < 0.001 |
| **Laboratory tests** |  |  |  |  |  |  |  |  |
| BUN, mg/dL | 20.0 (15.0, 31.0) | 25.0 (16.0, 43.0) | 19.0 (14.0, 27.0) | < 0.001 | 59.9 ± 35.5 | 61.6 ± 34.3 | 58.7 ± 36.4 | 0.538 |
| Creatinine, mg/dL | 1.0 (0.8, 1.5) | 1.2 (0.8, 1.8) | 1.0 (0.8, 1.4) | < 0.001 | 5.8 ± 3.7 | 5.6 ± 4.3 | 5.8 ± 3.2 | 0.677 |
| Glucose, mg/dL | 129.5 ± 25.4 | 132.0 ± 25.7 | 128.5 ± 25.3 | < 0.001 | 132.4 ± 28.0 | 132.3 ± 27.3 | 132.5 ± 28.6 | 0.964 |
| PaO_2_, mmHg | 103.6 ± 54.3 | 97.3 ± 58.5 | 106.0 ± 52.5 | < 0.001 | 84.9 ± 39.9 | 79.5 ± 43.5 | 88.4 ± 37.0 | 0.086 |
| PaCO_2_, mmHg | 46.7 ± 9.7 | 42.5 ± 11.2 | 48.2 ± 8.6 | < 0.001 | 47.7 ± 11.4 | 47.0 ± 12.1 | 48.1 ± 10.9 | 0.429 |
| Lactate, mmol/L | 2.2 (1.5, 3.3) | 1.6 (1.1, 2.5) | 2.4 (1.7, 3.5) | < 0.001 | 2.0 (1.2, 4.1) | 1.6 (1.1, 2.7) | 2.3 (1.4, 4.7) | 0.003 |
| Sodium, mmol/L | 137.0 ± 4.0 | 136.2 ± 4.9 | 137.2 ± 3.5 | < 0.001 | 135.2 ± 4.5 | 134.9 ± 4.7 | 135.4 ± 4.3 | 0.421 |
| Potassium, mmol/L | 4.6 ± 0.8 | 4.5 ± 0.8 | 4.6 ± 0.7 | 0.043 | 5.3 ± 1.1 | 5.2 ± 1.1 | 5.3 ± 1.2 | 0.484 |
| Hemoglobin, g/dL | 9.5 ± 1.9 | 9.7 ± 1.9 | 9.4 ± 1.8 | < 0.001 | 8.9 ± 2.0 | 9.0 ± 2.1 | 8.8 ± 1.9 | 0.296 |
| Platelets, x10^9^/L | 177.7 ± 105.1 | 210.1 ± 121.4 | 165.8 ± 95.7 | < 0.001 | 159.7 ± 95.1 | 165.0 ± 83.3 | 156.2 ± 102.4 | 0.48 |
| WBC, x10^9^/L | 15.5 ± 8.8 | 14.6 ± 9.2 | 15.8 ± 8.6 | < 0.001 | 15.8 ± 8.6 | 14.7 ± 7.5 | 16.6 ± 9.2 | 0.099 |
| INR | 1.6 ± 1.0 | 1.8 ± 1.2 | 1.6 ± 0.9 | < 0.001 | 1.5 (1.3, 2.1) | 1.6 (1.3, 2.2) | 1.5 (1.3, 2.0) | 0.162 |
| PT, seconds | 17.7 ± 10.0 | 19.1 ± 12.5 | 17.2 ± 8.9 | < 0.001 | 20.8 ± 12.5 | 21.9 ± 13.7 | 20.2 ± 11.7 | 0.315 |
| PTT, seconds | 43.9 ± 28.0 | 45.5 ± 30.9 | 43.4 ± 27.0 | 0.039 | 48.0 ± 29.2 | 44.9 ± 25.6 | 49.9 ± 31.1 | 0.2 |
| **Site of infection, n (%)** |  |  |  |  |  |  |  |  |
| Intestinal infection | 66 ( 1.5) | 16 (1.4) | 50 (1.6) | 0.621 | 3 ( 1.2) | 1 (1) | 2 (1.4) | 1 |
| Catheter infection | 63 ( 1.4) | 27 (2.3) | 36 (1.1) | 0.004 | 12 ( 4.9) | 6 (6.1) | 6 (4.1) | 0.55 |
| Skin and soft tissue infection | 2 ( 0.0) | 1 (0.1) | 1 (0) | 0.466 | 2 ( 0.8) | 2 (2) | 0 (0) | 0.158 |
| Urinary infection | 461 (10.5) | 170 (14.4) | 291 (9.1) | < 0.001 | 29 (11.8) | 13 (13.3) | 16 (10.8) | 0.559 |
| Pulmonary infection | 841 (19.2) | 283 (24) | 558 (17.5) | < 0.001 | 49 (19.9) | 18 (18.4) | 31 (20.9) | 0.62 |
| **Scores** |  |  |  |  |  |  |  |  |
| Charlson comorbidity index | 5.5 ± 2.7 | 6.4 ± 3.0 | 5.2 ± 2.6 | < 0.001 | 7.0 ± 2.7 | 7.7 ± 2.2 | 6.6 ± 2.9 | 0.001 |
| SOFA | 3.7 ± 2.0 | 3.3 ± 1.7 | 3.9 ± 2.0 | < 0.001 | 5.8 ± 2.7 | 5.0 ± 2.3 | 6.3 ± 2.8 | < 0.001 |
| SAPSII | 40.7 ± 13.9 | 38.0 ± 12.8 | 41.8 ± 14.1 | < 0.001 | 51.5 ± 14.4 | 46.2 ± 12.3 | 55.0 ± 14.7 | < 0.001 |
| **Treatments, n (%)** |  |  |  |  |  |  |  |  |
| First day vasopressor | 2452 (56.1) | 307 (26.1) | 2145 (67.1) | < 0.001 | 135 (55.1) | 39 (39.8) | 96 (65.3) | < 0.001 |
| First day ventilation | 2481 (56.7) | 246 (20.9) | 2235 (69.9) | < 0.001 | 146 (59.6) | 27 (27.6) | 119 (81) | < 0.001 |

| **Variables** | **Matched cohort** | | | | | | | |
| --- | --- | --- | --- | --- | --- | --- | --- | --- |
|  | **RRT=No** | | | | **RRT=Yes** | | | |
|  | **Total (n = 946)** | **Non-sedative use (n=471)** | **Sedative use (n=475)** | ***P*-value** | **Total (n = 76)** | **Non-sedative use (n=40)** | **Sedative use (n=36)** | ***P*-value** |
| **Demographic characteristics** |  |  |  |  |  |  |  |  |
| Male | 529 (55.9) | 268 (56.9) | 261 (54.9) | 0.545 | 44 (57.9) | 21 (52.5) | 23 (63.9) | 0.315 |
| Age, years | 67.0 ± 14.9 | 66.6 ± 15.0 | 67.4 ± 14.8 | 0.412 | 65.2 ± 14.5 | 67.8 ± 13.4 | 62.4 ± 15.3 | 0.101 |
| White | 682 (72.1) | 340 (72.2) | 342 (72) | 0.949 | 38 (50.0) | 21 (52.5) | 17 (47.2) | 0.646 |
| **Vital signs, Mean ± SD** |  |  |  |  |  |  |  |  |
| Heart rate, beats per minute | 87.7 ± 16.1 | 87.7 ± 16.3 | 87.7 ± 15.9 | 0.986 | 88.5 ± 16.4 | 85.9 ± 14.9 | 91.4 ± 17.6 | 0.144 |
| Systolic blood pressure, mmHg | 114.6 ± 14.3 | 115.0 ± 15.4 | 114.2 ± 13.1 | 0.385 | 112.4 ± 17.7 | 109.1 ± 17.5 | 116.1 ± 17.5 | 0.084 |
| Diastolic blood pressure, mmHg | 60.2 ± 9.7 | 60.1 ± 10.2 | 60.3 ± 9.2 | 0.737 | 58.0 ± 11.1 | 56.2 ± 11.0 | 60.1 ± 11.0 | 0.129 |
| Mean blood pressure, mmHg | 76.0 ± 9.7 | 76.0 ± 10.5 | 75.9 ± 8.8 | 0.893 | 73.2 ± 11.2 | 71.4 ± 10.9 | 75.2 ± 11.3 | 0.132 |
| Respiratory rate, breaths per minute | 19.5 ± 4.1 | 19.5 ± 4.2 | 19.5 ± 4.0 | 0.936 | 20.7 ± 3.7 | 19.4 ± 3.0 | 22.1 ± 3.9 | 0.001 |
| Body temperature, ℃ | 36.9 ± 0.5 | 36.9 ± 0.5 | 36.9 ± 0.5 | 0.828 | 36.8 ± 0.7 | 36.8 ± 0.6 | 36.8 ± 0.9 | 0.851 |
| SpO_2_, % | 97.0 ± 1.9 | 97.0 ± 1.8 | 97.0 ± 2.0 | 0.67 | 96.5 ± 2.8 | 97.1 ± 1.9 | 95.8 ± 3.5 | 0.045 |
| **Laboratory tests** |  |  |  |  |  |  |  |  |
| BUN, mg/dL | 21.0 (15.0, 35.0) | 22.0 (15.0, 37.0) | 21.0 (15.0, 34.0) | 0.435 | 69.6 ± 43.6 | 58.7 ± 31.3 | 81.6 ± 51.8 | 0.021 |
| Creatinine, mg/dL | 1.0 (0.8, 1.6) | 1.1 (0.8, 1.6) | 1.0 (0.8, 1.5) | 0.465 | 5.7 ± 3.3 | 5.0 ± 2.7 | 6.4 ± 3.8 | 0.061 |
| Glucose, mg/dL | 133.8 ± 25.2 | 134.1 ± 24.8 | 133.5 ± 25.7 | 0.703 | 132.5 ± 28.1 | 128.3 ± 26.5 | 137.1 ± 29.4 | 0.171 |
| PaO_2_, mmHg | 109.1 ± 68.4 | 110.1 ± 67.3 | 108.1 ± 69.5 | 0.661 | 87.9 ± 44.3 | 85.6 ± 49.0 | 90.4 ± 38.9 | 0.634 |
| PaCO_2_, mmHg | 45.3 ± 10.5 | 45.1 ± 11.5 | 45.6 ± 9.4 | 0.438 | 45.8 ± 12.5 | 48.8 ± 13.6 | 42.4 ± 10.3 | 0.024 |
| Lactate, mmol/L | 1.8 (1.2, 2.6) | 1.7 (1.2, 2.6) | 1.8 (1.2, 2.6) | 0.673 | 1.9 (1.3, 3.0) | 1.9 (1.3, 3.0) | 1.8 (1.3, 3.2) | 0.564 |
| Sodium, mmol/L | 136.8 ± 4.3 | 136.7 ± 4.4 | 136.9 ± 4.2 | 0.424 | 135.4 ± 4.7 | 135.2 ± 4.6 | 135.6 ± 4.9 | 0.692 |
| Potassium, mmol/L | 4.5 ± 0.7 | 4.5 ± 0.7 | 4.5 ± 0.7 | 0.933 | 5.0 ± 0.9 | 5.1 ± 0.9 | 5.0 ± 1.0 | 0.695 |
| Hemoglobin, g/dL | 9.6 ± 1.9 | 9.6 ± 2.0 | 9.6 ± 1.9 | 0.867 | 9.0 ± 2.0 | 8.9 ± 2.1 | 9.1 ± 2.0 | 0.704 |
| Platelets, x10^9^/L | 202.0 ± 119.3 | 202.8 ± 116.0 | 201.2 ± 122.6 | 0.84 | 150.6 ± 95.3 | 140.6 ± 77.5 | 161.7 ± 111.9 | 0.339 |
| WBC, x10^9^/L | 13.5 (9.7, 18.1) | 13.3 (9.4, 18.8) | 13.6 (9.8, 17.6) | 0.872 | 16.2 ± 10.6 | 14.1 ± 7.1 | 18.4 ± 13.3 | 0.076 |
| INR | 1.3 (1.2, 1.6) | 1.3 (1.2, 1.6) | 1.3 (1.2, 1.6) | 0.278 | 1.6 (1.3, 2.1) | 1.6 (1.3, 2.4) | 1.6 (1.3, 1.8) | 0.24 |
| PT, seconds | 14.8 (13.3, 17.5) | 14.5 (13.1, 17.5) | 15.0 (13.5, 17.4) | 0.188 | 17.0 (14.3, 22.6) | 17.3 (15.2, 25.3) | 17.0 (13.6, 19.4) | 0.164 |
| PTT, seconds | 32.7 (28.4, 43.3) | 32.9 (28.6, 43.8) | 32.7 (28.3, 42.3) | 0.861 | 45.4 ± 24.5 | 45.4 ± 22.3 | 45.5 ± 27.2 | 0.993 |
| **Site of infection, n (%)** |  |  |  |  |  |  |  |  |
| Intestinal infection | 15 ( 1.6) | 8 (1.7) | 7 (1.5) | 0.782 | **NA** | **NA** | **NA** | 1 |
| Catheter infection | 14 ( 1.5) | 7 (1.5) | 7 (1.5) | 0.987 | 4 ( 5.3) | 3 (7.5) | 1 (2.8) | 0.617 |
| Skin and soft tissue infection | 1 ( 0.1) | 0 (0) | 1 (0.2) | 1 | 1 ( 1.3) | 1 (2.5) | 0 (0) | 1 |
| Urinary infection | 110 (11.6) | 51 (10.8) | 59 (12.4) | 0.445 | 7 ( 9.2) | 4 (10) | 3 (8.3) | 1 |
| Pulmonary infection | 213 (22.5) | 100 (21.2) | 113 (23.8) | 0.346 | 13 (17.1) | 6 (15) | 7 (19.4) | 0.607 |
| **Scores** |  |  |  |  |  |  |  |  |
| Charlson comorbidity index | 5.8 ± 2.9 | 5.7 ± 3.0 | 5.9 ± 2.9 | 0.399 | 7.2 ± 2.7 | 7.6 ± 2.2 | 6.9 ± 3.1 | 0.304 |
| SOFA | 3.5 ± 1.7 | 3.5 ± 1.8 | 3.5 ± 1.7 | 0.923 | 5.7 ± 2.7 | 5.5 ± 2.5 | 5.9 ± 2.9 | 0.483 |
| SAPSII | 38.4 ± 13.7 | 37.8 ± 13.3 | 39.0 ± 14.1 | 0.182 | 52.2 ± 13.0 | 50.4 ± 11.4 | 54.3 ± 14.4 | 0.184 |
| **Treatments, n (%)** |  |  |  |  |  |  |  |  |
| First day vasopressor | 360 (38.1) | 175 (37.2) | 185 (38.9) | 0.57 | 40 (52.6) | 23 (57.5) | 17 (47.2) | 0.37 |
| First day ventilation | 334 (35.3) | 150 (31.8) | 184 (38.7) | 0.027 | 39 (51.3) | 19 (47.5) | 20 (55.6) | 0.483 |

**Notes:** Data are presented as mean ± SD, median (Q1–Q3), or n (%), as appropriate. Ventilation indicates high-flow nasal cannula (HFNC), non-invasive ventilation (NIV), invasive mechanical ventilation (IMV), or tracheostomy ventilation on the first ICU day; RRT indicates renal replacement therapy initiated within the first 24 hours after ICU admission. Illness severity was assessed using SOFA and SAPS II (SAPS 3 not available in MIMIC-IV). Group comparisons used t-tests or Wilcoxon tests for continuous variables and χ²/Fisher’s exact tests for categorical variables.
