# Supplementary material for: Propofol provides a significant survival advantage in sepsis-associated encephalopathy: A retrospective cohort study investigating one-year all-cause mortality
Source: PLoS One. 2026 Feb 5;21(2):e0340371. doi: 10.1371/journal.pone.0340371 (PMC12875438; doi:10.1371/journal.pone.0340371)
Supplement: S14 Table — (DOCX) [file pone.0340371.s014.docx]

Supporting Information

**S14 Table. Respiratory parameters and ARDS severity of patients receiving mechanical ventilation (MV) within the first 24 hours after ICU admission.**

| **Variables** | **All MV patients (n = 2430)** | **Non-sedative use (n = 101)** | **Sedative use (n = 2329)** | **P-value** |
| --- | --- | --- | --- | --- |
| MIN PaO₂/FiO₂ ratio | 206.0 ± 107.4 | 202.4 ± 127.3 | 206.2 ± 106.7 | 0.766 |
| MIN PaO₂/FiO₂ ratio ≤300, n (%) | 1806 (82.9) | 60 (82.2) | 1746 (82.9) | 0.866 |
| ARDS severity (Berlin classification), n (%) |  |  |  | 0.07 |
| Mild (PaO₂/FiO₂ 200–300) | 647 (35.8) | 17 (28.3) | 630 (36.1) |  |
| Moderate (PaO₂/FiO₂ 100–200) | 820 (45.4) | 25 (41.7) | 795 (45.5) |  |
| Severe (PaO₂/FiO₂ <100) | 339 (18.8) | 18 (30) | 321 (18.4) |  |

**Notes:** Data are presented as mean ± SD, median (Q1–Q3), or n (%), as appropriate.
