# Supplementary material for: Propofol provides a significant survival advantage in sepsis-associated encephalopathy: A retrospective cohort study investigating one-year all-cause mortality
Source: PLoS One. 2026 Feb 5;21(2):e0340371. doi: 10.1371/journal.pone.0340371 (PMC12875438; doi:10.1371/journal.pone.0340371)
Supplement: S15 Table — (DOCX) [file pone.0340371.s015.docx]

Supporting Information

**S15 Table. ICU length of stay and mortality in the original and matched cohorts.**

| **Variables** | **Original cohort** | | | | **Matched cohort** | | | |
| --- | --- | --- | --- | --- | --- | --- | --- | --- |
|  | **Total (n = 4618)** | **Sedative use (n=3343)** | **Non-sedative use (n=1275)** | ***P*-value** | **Total (n =1022)** | **Sedative use (n=511)** | **Non-sedative use (n=511)** | ***P*-value** |
| ICU LOS, days | 3.4 (1.9, 6.9) | 3.4 (1.8, 7.0) | 3.2 (1.9, 6.3) | 0.417 | 3.6 (1.9, 7.1) | 4.0 (2.1, 8.0) | 3.1 (1.8, 6.1) | 0.001 |
| ICU Mortality | 479 (10.4) | 284 (8.5) | 195 (15.3) | < 0.001 | 133 (13.0) | 63 (12.3) | 70 (13.7) | 0.515 |

**Notes:** Data are presented as mean ± SD, median (Q1–Q3), or n (%), as appropriate. LOS indicates ICU length of stay.
