# Supplementary material for: Propofol provides a significant survival advantage in sepsis-associated encephalopathy: A retrospective cohort study investigating one-year all-cause mortality
Source: PLoS One. 2026 Feb 5;21(2):e0340371. doi: 10.1371/journal.pone.0340371 (PMC12875438; doi:10.1371/journal.pone.0340371)

Supporting Information

**S1 Fig. Standardized mean difference of variables before matched, after PSM and after weighted OW. (A) Sedative use; (B) Propofol.**


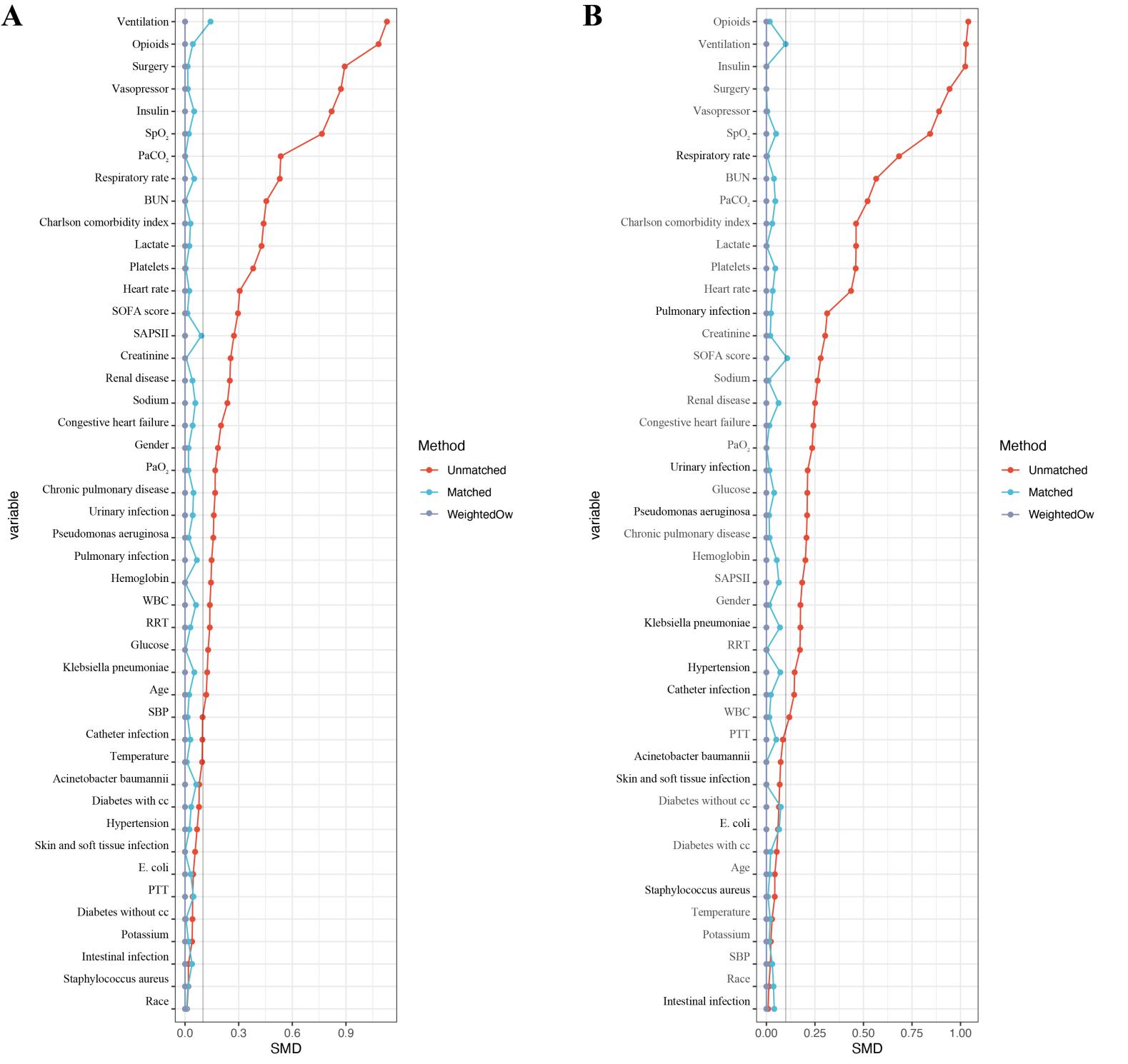

Supplement: S1 Fig — (DOCX) [file pone.0340371.s016.docx]
