# Supplementary figures and images for: Propofol provides a significant survival advantage in sepsis-associated encephalopathy: A retrospective cohort study investigating one-year all-cause mortality
Source: PLoS One. 2026 Feb 5;21(2):e0340371. doi: 10.1371/journal.pone.0340371 (PMC12875438; doi:10.1371/journal.pone.0340371)

Supporting Information

**S2 Fig. E-value of Sedative Use. (A) Original cohort; (B) Matched cohort.**


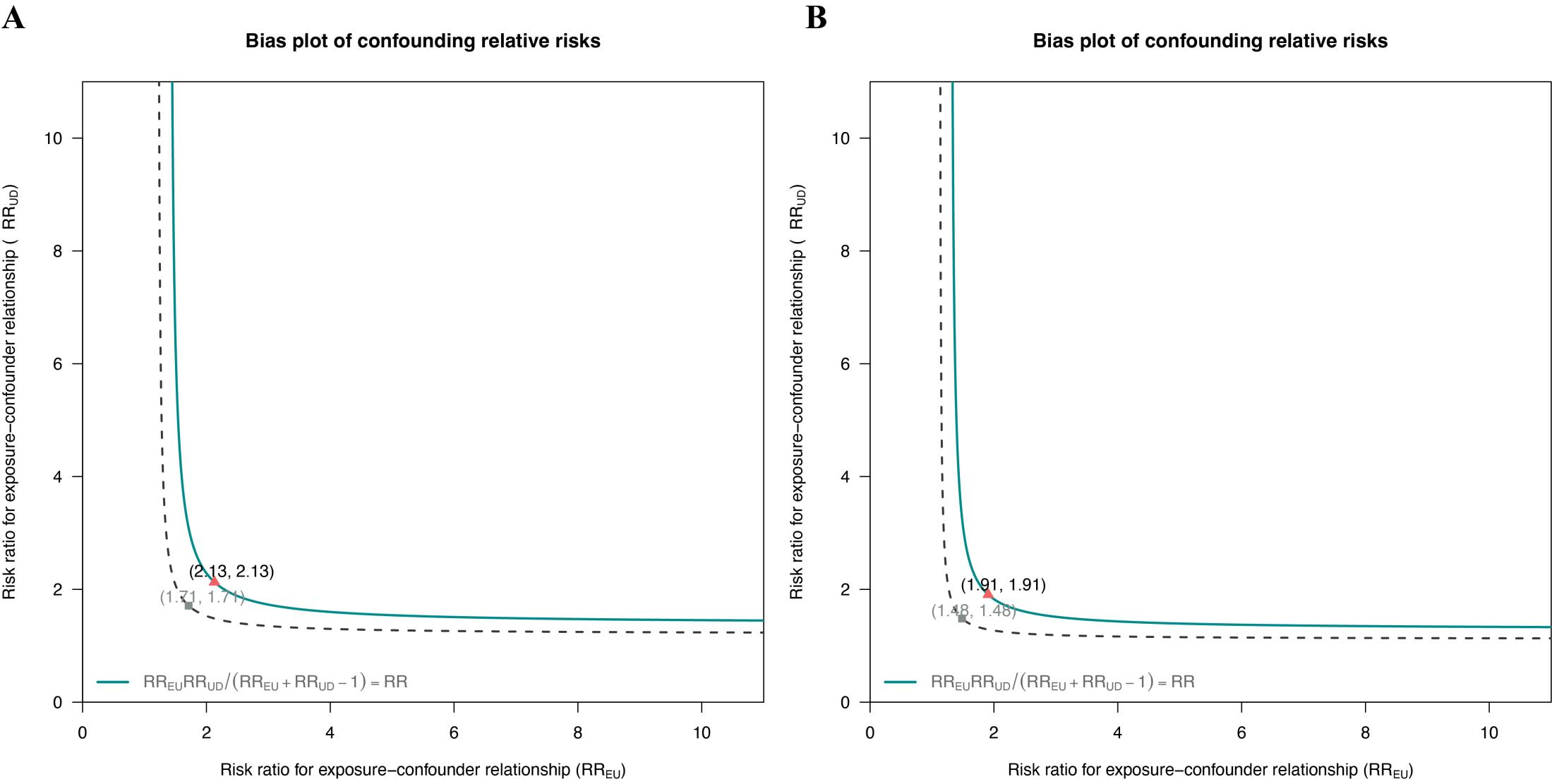

Supplement: S2 Fig — (DOCX) [file pone.0340371.s017.docx]

Supporting Information

**S3 Fig. E-value of Propofol. (A) Original cohort; (B) Matched cohort.**


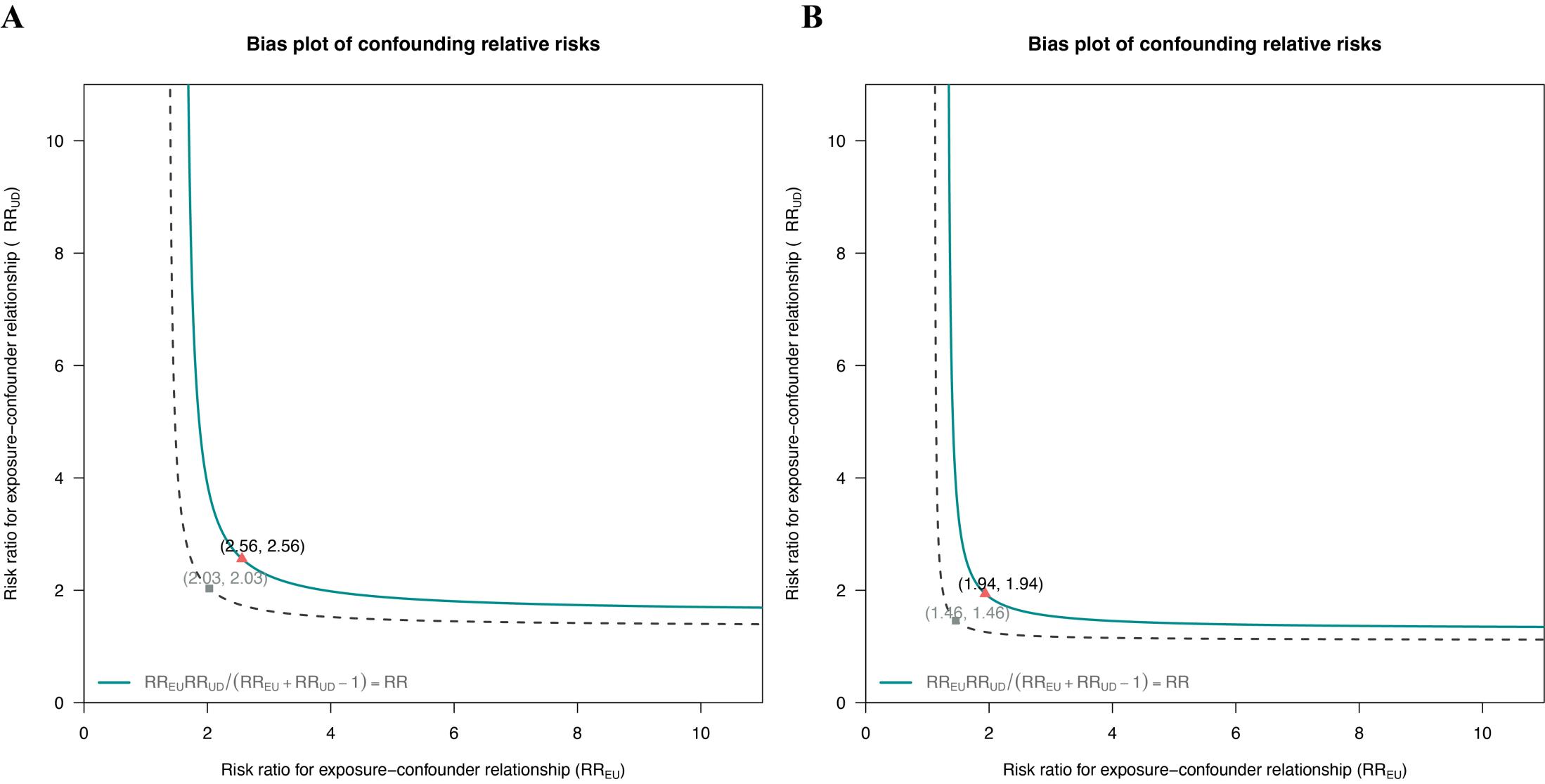

Supplement: S3 Fig — (DOCX) [file pone.0340371.s018.docx]
