# Supplementary material for: Propofol provides a significant survival advantage in sepsis-associated encephalopathy: A retrospective cohort study investigating one-year all-cause mortality
Source: PLoS One. 2026 Feb 5;21(2):e0340371. doi: 10.1371/journal.pone.0340371 (PMC12875438; doi:10.1371/journal.pone.0340371)
Supplement: S4 Fig — (DOCX) [file pone.0340371.s019.docx]

Supporting Information

**S4 Fig. Distribution of representative continuous variables between sedative-use and non–sedative-use groups. (A) Respiratory rate; (B) SAPS II; (C) Blood urea nitrogen (BUN); (D) Lactate.**


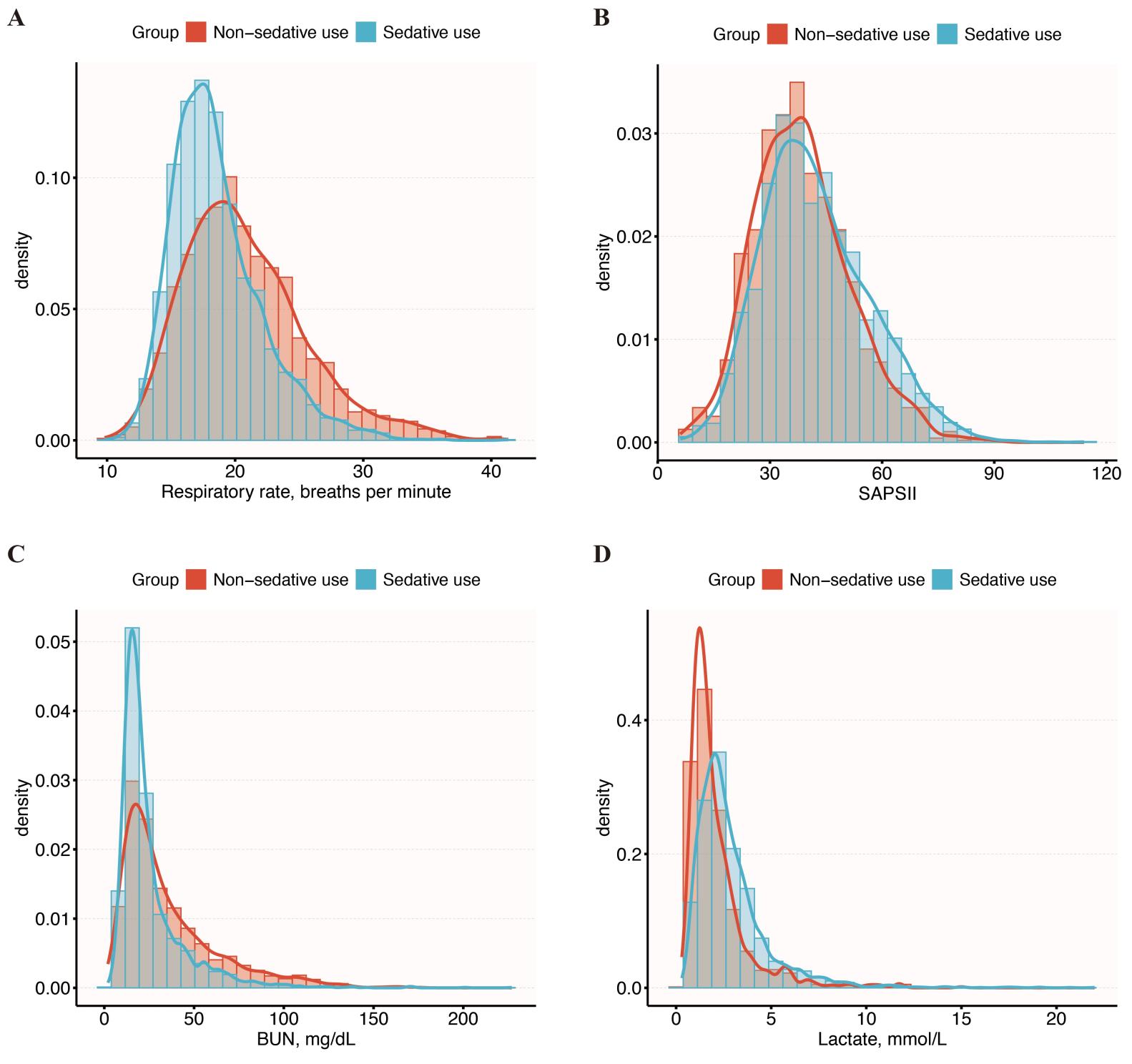


Continuous variables were visually inspected using histograms with kernel density curves to assess distributional shape and determine appropriate descriptive statistics.
